# Supplementary material for: Influence of Rotational Nucleosome Positioning on Transcription Start Site Selection in Animal Promoters
Source: PLoS Comput Biol. 2016 Oct 7;12(10):e1005144. doi: 10.1371/journal.pcbi.1005144 (PMC5055345; doi:10.1371/journal.pcbi.1005144)
Supplement: S1 Text — File containing a detailed description of the input data and the R code, properly commented, that has been used to make all the calculations and to generate all the figures. The file contains the R code for the figures and the functions used. (PDF) [file pcbi.1005144.s001.pdf]

Data Reproduction Guide

1. Introduction

Important note: All URLs in this paper are temporary. The files hyperlinked to this document will move to a permanent place once the accompanying article is accepted for publication by a journal.

The main purpose of this document is to meet high standards of “Reproducible Research” for the results reported in the accompanying article. To achieve this goal we provide stable links to archived data plus R script to reproduce all intermediate results and figures.

A secondary goal is to make it easy for colleagues to carry out similar studies. We have tried to present most procedures in R (v. 3.2.3) so that interested people do not have to download additional code or master another scripting language.

The complete R code is presented in this document along with comments. An R code file in unformatted text is also available. All results presented in the accompanying paper can be reproduced by copy-pasting the code into an R terminal window. Analyses procedures involving more than a few lines code are provided as R functions. The R code file and the functions file are provided at:

```
ftp://ccg.vital-it.ch/dreos16/data_reproduction_guide.R
ftp://ccg.vital-it.ch/dreos16/dreos16_functions.R
```

If this file is executed first

```
source(“dreos16_functions.R”)
```

then the functions presented in the final section of this guide do not need be defined by copy-pasting the code into an R window.

2 Source data – input files

The source data and input files are available at

```
ftp://ccg.vital-it.ch/dreos16/
```

2.1. Promoters, TSS coordinates

The analysis is based on promoter sets from EPDNew (1) for human (v2), mouse (v2), fly (v2), zebrafish (v1) and worm (v1). These promoter sets are provided in different formats. In the following, we will need the BED and SGA versions.

These are tab-delimited files containing the genomic coordinates of the TSS relative to the hg19, mm9, danRer7, dm3, ce6 genome assemblies (UCSC codes). These files were used to extract promoter sequences in the range -1074 to +1075 relative the transcriptions start site using the UCSC table browser (2). The resulting FASTA-formatted sequence files are available under the following filenames:

```
Hs_EPDnew_002a_hg19.seq
```

```
Mm_EPDnew_002_mm9.seq
Dr_EPDnew_001_danRer7.seq
Dm_EPDnew_002_dm3.seq
Ce_EPDnew_001_ce6.seq
```

2.2. CAGE data

2.2.1 Human

Human CAGE data were originally downloaded from the ENCODE consortium (3):

http://hgdownload.cse.ucsc.edu/goldenPath/hg19/encodeDCC/wgEncodeRikenCage

A pre-processed version of the data is available from the Mass Genome Annotation (MGA) data repository linked to the EPD (1):

http://ccg.vital-it.ch/mga/hg19/encode/GSE34448/all_cell_longPolyA.sga.gz

These file comprise CAGE tags from all libraries from long polyA cell extract in compact SGA format (see here for a format description). We have used this file to generate CAGE tag profiles with the same format as the input sequences files using the on-line tool ChIP-Extract that is part of the ChIP-Seq web server. Each row represents the CAGE tag distribution from -103 to +104 bp relative to the TSS. The file is available under the name Hs_EPDnew_002a_hg19_CAGEtags.dat.

CAGE data for the other organisms were generated using a similar pipeline. Row data used to generate them is briefly described next.

2.2.2 Mouse

Mouse CAGE data was from (4) and downloaded from:

http://fantom.gsc.riken.jp/5/datafiles/latest/basic/

the pre-processed version is available at the MGA repository:

ftp://ccg.vital-it.ch/mga/mm9/fantom5/allAdultTissues.sga.gz

that comprise all samples. Promoters CAGE tag profile file is available under the name Mm_EPDnew_002_mm9_CAGEtags.dat.

2.2.3 Zebrafish

D. rerio CAGE data was from (5) and downloaded from:

http://www.ncbi.nlm.nih.gov/sra/?term=sra055273

the pre-processed version is available at the MGA repository:

ftp://ccg.vital-it.ch/mga/danRer7/nepal13/Dr_allCageNepal13.sga.gz

that comprise all samples. Promoters CAGE tag profile file is available under the name Dr_EPDnew_001_danRer7_CAGEtags.dat.

2.2.4 Fruit fly

D. melanogaster CAGE data was from SRA ID SRP001602 and downloaded from:

http://www.ncbi.nlm.nih.gov/sra/?term=sra001602

the pre-processed version is available at the MGA repository:

ftp://ccg.vital-it.ch/mga/dm3/encode/SRP001602/Dm3_allCageSRP001602.sga.gz

that comprise all samples. Promoters CAGE tag profile file is available under the name Dm_EPDnew_002_dm3_CAGEtags.dat.

2.2.5 Worm

C. elegans CAGE data was from (6) and downloaded from:

http://www.ncbi.nlm.nih.gov/geo/query/acc.cgi?acc=GSE43087

the pre-processed version is available at the MGA repository:

ftp://ccg.vital-it.ch/mga/ce6/kruesi13/Ce6_allKruesi14GroCapSamples.sga.gz

that comprise all samples. Promoters CAGE tag profile file is available under the name Ce_EPDnew_001_ce6_CAGEtags.dat.

2.3. Nucleosome / histone data

2.3.1 Human MNase data

Human MNase-Seq data for the lymphoblastoid cell line GM18507 (7) was originally downloaded from:

http://www.ncbi.nlm.nih.gov/geo/query/acc.cgi?acc=GSM907783

A pre-processed version is available from the MGA repository at

ftp://ccg.vital-it.ch/mga/hg19/gaffney12/GSM907783.sga.gz

This files contains the mapped MNase sequence tags in SGA format. Tags were first centered using the program chipcenter from the ChIP-Seq software package version 1 which can be downloaded from:

http://sourceforge.net/projects/chip-seq

The centering process shifts the tags mapping to + and - strand of the chromosome sequence by a fixed distance upstream and downstream, respectively. We used a centering distance of 70 bp, approximately half a nucleosome length. Centering has the effect that each tag position points to the nucleosome center, and the + and - tags for the same nucleosome have maxima at the same position. MNase tag profiles for individual promoters were then generated as before using ChIP-Extract and the tag matrix file is available under the name: Hs_EPDnew_vs_GSM907783.dat

2.3.2: Mouse MNase-Seq data

Mouse MNase-Seq data was conducted on B cell (8) and was originally downloaded from:

5http://www.ncbi.nlm.nih.gov/geo/query/acc.cgi?acc=GSM1293995

A pre-processed version is available from the MGA repository at

ftp://ccg.vital-it.ch/mga/mm9/asp11/GSM721292_MB_H3K4me3.sga.gz

The ChIP-seq tag matrix file is available under the name: Mm_EPDnew_vs_GSM1293995.dat

2.3.3 Zebrafish MNase data

Zebrafish MNase-seq data was conducted on embryos in dome stage (9) and was originally downloaded from:

http://www.ncbi.nlm.nih.gov/geo/query/acc.cgi?acc=GSM1081554

A pre-processed version is available from the MGA repository at

ftp://ccg.vital-it.ch/mga/danRer7/zhang14/GSM1081554.sga.gz

The MNase-seq tag matrix file is available under the name: Dr_EPDnew_vs_GSM1081555.dat

2.3.4 Fruit fly MNase-seq data

D. melanogaster MNase-seq data was from (10) and was originally downloaded from:

http://www.ncbi.nlm.nih.gov/geo/query/acc.cgi?acc=GSM1248974

A pre-processed version is available from the MGA repository at

ftp://ccg.vital-it.ch/mga/dm3/bohla14/GSM1248974.sga.gz

The ChIP-seq tag matrix file is available under the name: Dm_EPDnew_vs_GSM1248974.dat

2.3.5 Worm H3K4me3 ChIP-seq data

C. elegans MNase-seq data was from (11) and was originally downloaded from:

http://trace.ncbi.nlm.nih.gov/Traces/sra/?study=SRP000191

A pre-processed version is available from the MGA repository at

http://ccg.vital-it.ch/mga/ce6/valouev08/MNase_sample.sga.gz

The ChIP-seq tag matrix file is available under the name: Ce_EPDnew_vs_valouev08.out

2.5 Promoter annotation files

The following are tab delimited organism-specific files in which each row corresponds to a promoter and each column to an important promoter feature (TATA-box, Initiator, CpG island (only for human and mouse)). TATA-box and Initiator motifs are based on (12). The presence of a feature in a promoter is represented as 1 whereas its absence as 0. A promoter is *with* a TATA-box if the motif is found at position −28 (± 3 bp) from the transcription start site (evaluated using FindM), *with* an Initiator motif if it is found at position 0 from the transcription start site and has a CpG island if the island cover the TSS (CpG island BED files for human and mouse were downloaded from UCSC Genome Browser).

```
Hs_promoterAnnotation.txt
Mm_promoterAnnotation.txt
Dr_promoterAnnotation.txt
Dm_promoterAnnotation.txt
Ce_promoterAnnotation.txt
```

3. Data Analysis Procedures

This Section describes all data analysis procedures necessary to reproduce the results shown in this paper from the source data described in the previous section to numerical output that directly served as input for the figures. The code for generating the Figures is provided in the next Section. Scientists only interested in reproducing the Figures can skip this section and proceed with the next.

3.1. Read promoter sequences into R and transform them into integer matrix

For all procedures, the FASTA-formatted promoter sequence files are converted into integer matrixes. Each row corresponds to a promoter sequence and each column to a position. The promoter identifier is provided by row name. The format conversion is achieved as follows: first FASTA-formatted file is read into R using the "read.fasta" function provided in the package "seqinr" (13) available from CRAN (can be easily installed using "install.packages" function). This function generates a list that is converted into a matrix. Then, each base is converted into an integer (N=0; A=1; C=2; G=3; T=4) using the function "seq.trans" (described in "Functions definition" section of this document). The following is the code to generate the promoter matrix for human (explicated in all the steps) and a condensed version for the other 4 organisms.

```
#Install.packages("seqinr")
library("seqinr")

hs.raw.sequences <- read.fasta("data/Hs_EPDnew_002a_hg19.seq")
hs.names <- names(hs.raw.sequences)
hs.sequences <- matrix(data=unlist(hs.raw.sequences),
       nrow=length(hs.raw.sequences), ncol=length(hs.raw.sequences[[1]]),
       byrow=TRUE)
rownames(hs.sequences) <- hs.names
hs.promoters <- seq.trans(hs.sequences)

# Repeat the analysis on all organisms:
organisms <- c("hs","mm","dr","dm","ce")
fasta.names <- c("data/Hs_EPDnew_002a_hg19.seq",
       "data/Mm_EPDnew_002_mm9.seq", "data/Dr_EPDnew_001_danRer7.seq",
       "data/Dm_EPDnew_002_dm3.seq", "data/Ce_EPDnew_001_ce6.seq")
names(fasta.names) <- organisms

for (J in organisms){
      result.object <- paste(J, "promoters", sep=".")
      raw.sequences <- read.fasta(fasta.names[J])
      seq.names <- names(raw.sequences)
      mat.sequences <- matrix(data=unlist(raw.sequences),
             nrow=length(raw.sequences), ncol=length(raw.sequences[[1]]),
             byrow=TRUE)
      rownames(mat.sequences) <- seq.names
      assign(result.object, seq.trans(mat.sequences))
}

colnames(hs.promoters) <- colnames(mm.promoters) <- colnames(dr.promoters)
       <- colnames(dm.promoters) <- colnames(ce.promoters) <- -1074:1075
```

This code generates 5 objects (hs.promoters, mm.promoters, dr.promoters, dm.promoters and ce.promoters) that will be used as input in the following sub-sections.

3.2. Promoter annotation with pre-compiled files

Files named XX_promoterAnnotation.txt (where XX is a two digit code for the species) contain the information on presence or absence of important DNA-encoded promoter features such as TATA-

box, Initiator element and CpG island (only for human and mouse). This subsection will read the files into matrices and generate vectors containing promoter IDs for the TATA+ promoters, TATA- / Inr+ promoters and TATA- / Inr- promoters. Moreover, for D. melanogaster the presence of the DPE element is also annotated.

```
hs.promoterAnnotation <- read.table("data/Hs_promoterAnnotation.txt",
       header=T, row.names=1)
mm.promoterAnnotation <- read.table("data/Mm_promoterAnnotation.txt",
       header=T, row.names=1)
dr.promoterAnnotation <- read.table("data/Dr_promoterAnnotation.txt",
       header=T, row.names=1)
dm.promoterAnnotation <- read.table("data/Dm_promoterAnnotation.txt",
       header=T, row.names=1)
ce.promoterAnnotation <- read.table("data/Ce_promoterAnnotation.txt",
       header=T, row.names=1)

hs.tata.promoters <- rownames(
       hs.promoterAnnotation[which(hs.promoterAnnotation$TATA == 1),])
hs.inr.promoters <- rownames(
       hs.promoterAnnotation[which(hs.promoterAnnotation$TATA == 0 &
       hs.promoterAnnotation$Inr == 1),])
hs.nocpe.promoters <- rownames(
       hs.promoterAnnotation[which(hs.promoterAnnotation$TATA == 0 &
       hs.promoterAnnotation$Inr == 0),])
hs.cpg.promoters <- rownames(
       hs.promoterAnnotation[which(hs.promoterAnnotation$CpG == 1),])
hs.nocpg.promoters <- rownames(
       hs.promoterAnnotation[which(hs.promoterAnnotation$CpG == 0),])
#
mm.tata.promoters <- rownames(
       mm.promoterAnnotation[which(mm.promoterAnnotation$TATA == 1),])
mm.inr.promoters <- rownames(
       mm.promoterAnnotation[which(mm.promoterAnnotation$TATA == 0 &
       mm.promoterAnnotation$Inr == 1),])
mm.nocpe.promoters <- rownames(
       mm.promoterAnnotation[which(mm.promoterAnnotation$TATA == 0 &
       mm.promoterAnnotation$Inr == 0),])
mm.cpg.promoters <- rownames(
       mm.promoterAnnotation[which(mm.promoterAnnotation$CpG == 1),])
mm.nocpg.promoters <- rownames(
       mm.promoterAnnotation[which(mm.promoterAnnotation$CpG == 0),])
#
dr.tata.promoters <- rownames(
       dr.promoterAnnotation[which(dr.promoterAnnotation$TATA == 1),])
dr.inr.promoters <- rownames(
       dr.promoterAnnotation[which(dr.promoterAnnotation$TATA == 0 &
       dr.promoterAnnotation$Inr == 1),])
dr.nocpe.promoters <- rownames(
       dr.promoterAnnotation[which(dr.promoterAnnotation$TATA == 0 &
       dr.promoterAnnotation$Inr == 0),])
#
dm.tata.promoters <- rownames(
       dm.promoterAnnotation[which(dm.promoterAnnotation$TATA == 1),])
dm.inr.promoters <- rownames(
```

```
        dm.promoterAnnotation[which(dm.promoterAnnotation$TATA == 0 &
        dm.promoterAnnotation$Inr == 1),])
dm.nocpe.promoters <- rownames(
        dm.promoterAnnotation[which(dm.promoterAnnotation$TATA == 0 &
        dm.promoterAnnotation$Inr == 0),])
#
ce.tata.promoters <- rownames(
        ce.promoterAnnotation[which(ce.promoterAnnotation$TATA == 1),])
ce.inr.promoters <- rownames(
        ce.promoterAnnotation[which(ce.promoterAnnotation$TATA == 0 &
        ce.promoterAnnotation$Inr == 1),])
ce.nocpe.promoters <- rownames(
        ce.promoterAnnotation[which(ce.promoterAnnotation$TATA == 0 &
        ce.promoterAnnotation$Inr == 0),])
```

3.3. Evaluation of promoters' dinucleotides frequencies

This subsection will show how to calculate average dinucleotide frequencies around promoters using the function "find.freq" defined in the Functions definition section of this document. As done before, we start with an explanatory example of how to apply the function and then proceed with the loop to generate all the other cases. The result is a vector of frequencies for each position around promoters with the familiar name schema "JJ.KK.freq":

```
hs.yy.freq <- find.freq(hs.promoters, type="YY")

# Repeat the analysis for all dinucleotides and organisms
for (J in organisms){
      cat("Processing organism", J, "\n")
      for (K in dinucleotide){
            cat("   ", K, "\n")
            result.name <- paste(J, tolower(K), "freq", sep=".")
            assign(result.name, find.freq(get(paste(J, "promoters",
            sep=".")), type=K))
      }
}
```

Evaluation of dinucleotide frequencies for subset of promoters is simple:

```
hs.tata.yy.freq <- find.freq(hs.promoters[hs.tata.promoters,], type="YY")
hs.inr.yy.freq <- find.freq(hs.promoters[hs.inr.promoters,], type="YY")
hs.nocpe.yy.freq <- find.freq(hs.promoters[hs.nocpe.promoters,], type="YY")
hs.cpg.yy.freq <- find.freq(hs.promoters[hs.cpg.promoters,], type="YY")
hs.nocpg.yy.freq <- find.freq(hs.promoters[hs.nocpg.promoters,], type="YY")
#
mm.tata.rr.freq <- find.freq(mm.promoters[mm.tata.promoters,], type="RR")
mm.inr.rr.freq <- find.freq(mm.promoters[mm.inr.promoters,], type="RR")
mm.nocpe.rr.freq <- find.freq(mm.promoters[mm.nocpe.promoters,], type="RR")
mm.cpg.rr.freq <- find.freq(mm.promoters[mm.cpg.promoters,], type="RR")
mm.nocpg.rr.freq <- find.freq(mm.promoters[mm.nocpg.promoters,], type="RR")
#
dr.tata.ww.freq <- find.freq(dr.promoters[dr.tata.promoters,], type="WW")
dr.inr.ww.freq <- find.freq(dr.promoters[dr.inr.promoters,], type="WW")
```

```
dr.nocpe.ww.freq <- find.freq(dr.promoters[dr.nocpe.promoters,], type="WW")
#
dm.tata.ww.freq <- find.freq(dm.promoters[dm.tata.promoters,], type="WW")
dm.inr.ww.freq <- find.freq(dm.promoters[dm.inr.promoters,], type="WW")
dm.nocpe.ww.freq <- find.freq(dm.promoters[dm.nocpe.promoters,], type="WW")
#
ce.tata.ww.freq <- find.freq(ce.promoters[ce.tata.promoters,], type="WW")
ce.inr.ww.freq <- find.freq(ce.promoters[ce.inr.promoters,], type="WW")
ce.nocpe.ww.freq <- find.freq(ce.promoters[ce.nocpe.promoters,], type="WW")
```

Dinucleotides frequencies for all organisms can be saved to a file for easy inspection and figure plotting. Note that this file is already been provided and can be used directly to make the figures.

```
din.freq <- cbind(hs.all.yy = hs.yy.freq,
                  hs.all.rr = hs.rr.freq,
                  hs.all.ww = hs.ww.freq,
                  hs.all.ss = hs.ss.freq,
                  hs.tata.yy = hs.tata.yy.freq,
                  hs.inr.yy = hs.inr.yy.freq,
                  hs.nocpe.yy = hs.nocpe.yy.freq,
                  hs.cpg.yy = hs.cpg.yy.freq,
                  hs.nocpg.yy = hs.nocpg.yy.freq,
                  mm.all.yy = mm.yy.freq,
                  mm.all.rr = mm.rr.freq,
                  mm.all.ww = mm.ww.freq,
                  mm.all.ss = mm.ss.freq,
                  mm.tata.rr = mm.tata.rr.freq,
                  mm.inr.rr = mm.inr.rr.freq,
                  mm.nocpe.rr = mm.nocpe.rr.freq,
                  mm.cpg.rr = mm.cpg.rr.freq,
                  mm.nocpg.rr = mm.nocpg.rr.freq,
                  dr.all.yy = dr.yy.freq,
                  dr.all.rr = dr.rr.freq,
                  dr.all.ww = dr.ww.freq,
                  dr.all.ss = dr.ss.freq,
                  dr.tata.ww = dr.tata.ww.freq,
                  dr.inr.ww = dr.inr.ww.freq,
                  dr.nocpe.ww = dr.nocpe.ww.freq,
                  dm.all.yy = dm.yy.freq,
                  dm.all.rr = dm.rr.freq,
                  dm.all.ww = dm.ww.freq,
                  dm.all.ss = dm.ss.freq,
                  dm.tata.ww = dm.tata.ww.freq,
                  dm.inr.ww = dm.inr.ww.freq,
                  dm.nocpe.ww = dm.nocpe.ww.freq,
                  ce.all.yy = ce.yy.freq,
                  ce.all.rr = ce.rr.freq,
                  ce.all.ww = ce.ww.freq,
                  ce.all.ss = ce.ss.freq,
```

```
                   ce.tata.ww = ce.tata.ww.freq,
                   ce.inr.ww = ce.inr.ww.freq,
                   ce.nocpe.ww = ce.nocpe.ww.freq
                   )
write.table(din.freq, file="data/DinucleotideFrequencies.txt", sep="\t",
       quote=T, row.names=T, col.names=T)
```

3.4 Promoter Spectra analysis

The function "all.spectra" is used here to analyze the spectra decomposition of dinucleotide around promoters. The function takes as input a region of 150 bp for all promoters and evaluates the spectra decomposition between frequency 2 bp and 20 bp (nspectra vector) and output an average for all sequences for each position determined by the interval vector. The following is the complete code for generating the human spectra analysis for WW dinucleotides in the region -1000 to 1000 bp from the TSS with 10 bp intervals:

```
intervals <- seq(-1000, 1000, 10)
nspectra <- seq(2,20,0.1)

hs.promoters.spectra.ww <- matrix(0, nrow=length(intervals),
  ncol=length(nspectra))
colnames(hs.promoters.spectra.ww) <- seq(2,20,0.1)
rownames(hs.promoters.spectra.ww) <- intervals
for (I in intervals){
      region <- as.character(seq(I-74, I+75, 1))
      hs.region <- hs.promoters[,region]
      hs.region.nas <- matrix(ncol=length(nspectra),
      nrow=dim(hs.region)[1])
      for (K in 1:dim(hs.region)[1]){
           hs.region.nas[K,] <- all.spectra(hs.region[K,], "WW")
      }
      final.nas.region <- colMeans(hs.region.nas, na.rm=T)
      hs.promoters.spectra.ww[which(intervals == I),] <- final.nas.region
      image(x=intervals, y=nspectra, z=hs.promoters.spectra.ww,
        zlim=range(hs.promoters.spectra.ww[hs.promoters.spectra.ww>0]),
        col=topo.colors(100), xlab="Distance from TSS", ylab="Spectrum
        Frequences", main="H. sapiens promoters - WW")
}
```

This chunk of code takes as input the transformed sequence matrix for human (hs.promoters), extract the 150 bp region of interest (the intervals `I`) of all promoters and applies the spectra decomposition for dinucleotide WW. The result is a matrix with the number of rows (`Y`) equal to the number of frequencies (nspectra) analyzed and number of columns (`X`) equal to the intervals. Each cell in the matrix corresponds to the average spectrum intensity evaluated at position `X` relative to the TSS.

The complete code for generating all spectra profiles for the other dinucleotides and organisms is presented below. It generates 20 objects names following the rule "JJ.promoters.spectra.KK" where JJ is the organism (hs, mm, dr, dm and ce) and KK is the dinucleotide (yy, rr, ww, ss). Please consider

the fact that this code can take up to a week to run and uses more that 10 GB of memory. However, if you have time or hardware constraints you can skip this step and load the saved results directly into R.

```
organisms <- c("hs","mm","dr","dm","ce")
dinucleotide <- c("YY","RR","WW","SS")

for (J in organisms){
      cat("Processing organism", J, "\n")
      for (K in dinucleotide){
            cat("   ", K, "\n")
            result.name <- paste(J, "promoters.spectra", tolower(K),
            sep=".")
            assign(result.name, vector(mode = "numeric"))
            for (I in intervals){
                  region <- as.character(seq(I-74, I+75, 1))
                  region.data <- get(paste(J,"promoters",sep="."))[,region]
                  region.spec <- all.spectra(region.data, K)
                  average.spec <- colMeans(region.spec, na.rm=T)
                  assign(result.name, cbind(get(result.name),
                    average.spec))
            }
            var <- get(result.name)
            colnames(var) <- intervals
            assign(result.name, var)
      }
}
```

Save the results on a file or load them:

```
save(hs.promoters.spectra.yy,
     hs.promoters.spectra.ww,
     hs.promoters.spectra.rr,
     hs.promoters.spectra.ss,
     dm.promoters.spectra.ww,
     dm.promoters.spectra.yy,
     dm.promoters.spectra.rr,
     dm.promoters.spectra.ss,
     dr.promoters.spectra.ww,
     dr.promoters.spectra.yy,
     dr.promoters.spectra.rr,
     dr.promoters.spectra.ss,
     ce.promoters.spectra.ww,
     ce.promoters.spectra.yy,
     ce.promoters.spectra.rr,
     ce.promoters.spectra.ss,
     mm.promoters.spectra.yy,
     mm.promoters.spectra.ww,
```

```
     mm.promoters.spectra.rr,
     mm.promoters.spectra.ss,
     file="data/promotersRawSpectra.RData")
```

3.5. Computation of 10.3 bp spectra for individual sequences.

The elementary function "raw.spectra" is used for computing the 10.3 bp spectra decomposition score for any input sequence of length 150 bases. It exploits the fact that nucleosomes prefer DNA sequences with dinucleotide periodicity of 10.3 bases. The function accepts 4 dinucleotides sequences: YY, RR, SS, WW. In this context it evaluates the strength of a 10.3 bp periodicity in the sequence for a given dinucleotide. Complete code for generating the human raw score profiles for WW dinucleotides in the region -1000 to 1000 bp from the TSS with 10 bp intervals:

```
intervals <- seq(-1000, 1000, 10)

hs.promoters.raw.yy <- matrix(0, ncol=length(intervals),
       nrow=dim(hs.promoters)[1])
colnames(hs.promoters.raw.yy) <- intervals
rownames(hs.promoters.raw.yy) <- rownames(hs.promoters)
for (I in intervals){
      region <- as.character(seq(I-74, I+75, 1))
      hs.region <- hs.promoters[,region]
      hs.region.raw <- apply(hs.region, 1, raw.spectra, "YY", 10.3)
      cat(I, "\t", mean(hs.region.raw, na.rm=T), "\n")
      hs.promoters.raw.yy[,which(intervals == I)] <- hs.region.raw
      plot(intervals, colMeans(hs.promoters.raw.yy, na.rm=T), type="b",
        pch=19, ylim=range(colMeans(hs.promoters.raw.yy,
        na.rm=T)[colMeans(hs.promoters.raw.yy, na.rm=T)>0]))
}
```

This chunk of code takes as input the integer transformed sequence matrix for human ("hs.promoters"), extract the 150 region of interest (the intervals `I`) of all promoters and applies the raw.spectra score for dinucleotide YY. The result is a matrix with the number of rows (`Y`) equal to the number of promoters analyzed and number of columns (`X`) equal to the intervals. Each cell in the matrix corresponds to the intensity of 10.3 bp spectrum value evaluated at position `X` (in a 150 bp window) relative to the TSS of the promoter `Y`.

The complete code for generating all NA score profiles for the other dinucleotides and organisms is presented below. It generates 20 objects names following the rule "JJ.promoters.eaw.KK" where JJ is the organism (hs, mm, dr, dm and ce) and KK is the dinucleotide (yy, rr, ww, ss).

```
organisms <- c("hs","mm","dr","dm","ce")
dinucleotide <- c("YY","RR","WW","SS")

for (J in organisms){
      cat("Processing organism", J, "\n")
      for (K in dinucleotide){
            cat("    ", K, "\n")
            result.name <- paste(J, "promoters.raw", tolower(K), sep=".")
```

```
            assign(result.name, vector(mode = "numeric"))
            for (I in intervals){
                  region <- as.character(seq(I-74, I+75, 1))
                  region.data <- get(paste(J,"promoters",sep="."))[,region]
                  region.nas <- apply(region.data, 1, raw.spectra, K, 10.3)
                  assign(result.name, cbind(get(result.name), region.nas))
            }
            var <- get(result.name)
            colnames(var) <- intervals
            assign(result.name, var)
      }
}
```

Save the objects:

```
save(hs.promoters.raw.yy,
     hs.promoters.raw.rr,
     hs.promoters.raw.ww,
     hs.promoters.raw.ss,
     mm.promoters.raw.yy,
     mm.promoters.raw.rr,
     mm.promoters.raw.ww,
     mm.promoters.raw.ss,
     dr.promoters.raw.yy,
     dr.promoters.raw.rr,
     dr.promoters.raw.ww,
     dr.promoters.raw.ss,
     dm.promoters.raw.yy,
     dm.promoters.raw.rr,
     dm.promoters.raw.ww,
     dm.promoters.raw.ss,
     ce.promoters.raw.yy,
     ce.promoters.raw.rr,
     ce.promoters.raw.ww,
     ce.promoters.raw.ss,
     file="data/promotersRawSignals.RData")
```

3.6. Correlation between dinucleotides and in-vivo nucleosomes

In this section we will present the steps to be taken to generate the data presented in Figure 1. This part of the code is focused on human data and it will be extensively commented to clarify each step. The code for all other organisms will be presented after without comments since it will be very similar to this. We will start with uploading the data for MNase experiments:

```
# Get the MNase data for human:
hs.nucleosomes <- read.table('data/Hs_EPDnew_vs_GSM907783.dat', header=F,
     row.names=1)[rownames(hs.promoters.raw.yy),]
colnames(hs.nucleosomes) <- -1000:1000
```

```
hs.m.nucleosomes <- matrix(nrow=dim(hs.nucleosomes)[1], ncol=201)
colnames(hs.m.nucleosomes) <- seq(-1000, 1000, 10)
rownames(hs.m.nucleosomes) <- rownames(hs.nucleosomes)

# this file is 1bp in steps, convert to a 10bp steps:
for (I in seq(-1000, 1000, 10)){
     if ( (I - 4) > -1000){
          gap.min <- I – 4
     }else{
          gap.min <- I
     }
     if ( (I + 5) < 1000){
          gap.max <- I + 5
     }else{
          gap.max <- I
     }
     gap <- as.character(gap.min:gap.max)
     hs.m.nucleosomes[,as.character(I)] <- rowSums(hs.nucleosomes[,gap],
        na.rm=T)
}
```

This chunk of code read the MNase data in the file 'data/Hs_EPDnew_vs_GSM907783.dat and reduce the number of steps to the same number as the intervals in the hs.promoter.raw.XX serie of objects created before. Now the correlation analysis:

```
ordering <- order(apply(hs.promoters.raw.ww, 1, cor,
   colMeans(hs.m.nucleosomes)))

# Order promoters according to ordering
hs.ww <- hs.promoters.raw.ww[ordering,]
hs.ww.mean <- condense.matrix(hs.ww, 20)

# Now the same for the nucleosomes
hs.nuc <- hs.m.nucleosomes[ordering,]
hs.nuc.mean <- condense.matrix(hs.nuc, 20)

# And for CpG islands and TATA-box distributions:
hs.cpg <- hs.annotation[ordering,3]
hs.cpg.m <- condense.matrix(as.matrix(hs.cpg), 20)
hs.tata <- hs.annotation[ordering,1]
hs.tata.m <- condense.matrix(as.matrix(hs.tata), 20)
```

The “ordering” object is a vector of indices for promoters with higher correlation to the average nucleosome distribution. Subsequently it is used to reorder the promoters, nucleosome and annotation objects. The “condense.matrix” function is used to reduce the matrix row numbers by

averaging groups of 20 rows column-wise. This procedure reduces the dimension of the image to be plotted without altering the output.

In the next chunk of code we will find promoters with high signal in the N+1 region and low signal in the NFR for the human promoters:

```
range.1n <- which(seq(-1000,1000,10) >= 50 & seq(-1000,1000,10) <= 200)
range.nfr <- which(seq(-1000,1000,10) >= -150 & seq(-1000,1000,10) <= -20)

hs.promotersDinucleotideCorrelation <- matrix(0, ncol=4,
   nrow=dim(hs.promoters.raw.ss)[1])
colnames(hs.promotersDinucleotideCorrelation) <- c("YY","RR","WW","SS")
rownames(hs.promotersDinucleotideCorrelation) <-
   rownames(hs.promoters.raw.ss)

average.yy.proxprom <- cbind(rowMeans(hs.promoters.raw.yy[,range.nfr],
   na.rm=T), rowMeans(hs.promoters.raw.yy[,range.1n], na.rm=T))
average.rr.proxprom <- cbind(rowMeans(hs.promoters.raw.rr[,range.nfr],
   na.rm=T), rowMeans(hs.promoters.raw.rr[,range.1n], na.rm=T))
average.ww.proxprom <- cbind(rowMeans(hs.promoters.raw.ww[,range.nfr],
   na.rm=T), rowMeans(hs.promoters.raw.ww[,range.1n], na.rm=T))
average.ss.proxprom <- cbind(rowMeans(hs.promoters.raw.ss[,range.nfr],
   na.rm=T), rowMeans(hs.promoters.raw.ss[,range.1n], na.rm=T))
average.nucl.proxprom <- cbind(rowMeans(hs.m.nucleosomes[,range.nfr]),
   rowMeans(hs.m.nucleosomes[,range.1n]))

hs.promotersDinucleotideCorrelation <- matrix(0, ncol=4,
   nrow=dim(hs.promoters.raw.ss)[1])
colnames(hs.promotersDinucleotideCorrelation) <- c("YY","RR","WW","SS")
rownames(hs.promotersDinucleotideCorrelation) <-
   rownames(hs.promoters.raw.ss)

hs.promotersDinucleotideCorrelation[which(average.yy.proxprom[,2] >
   mean(average.yy.proxprom)),1] <- 1
hs.promotersDinucleotideCorrelation[which(average.rr.proxprom[,2] >
   mean(average.rr.proxprom)),2] <- 1
hs.promotersDinucleotideCorrelation[which(average.ww.proxprom[,2] >
   mean(average.ww.proxprom)),3] <- 1
hs.promotersDinucleotideCorrelation[which(average.ss.proxprom[,2] >
   mean(average.ss.proxprom)),4] <- 1

fractionConcordanceDinucleotides <- cumsum(
     c(
     sum(rowSums(hs.promotersDinucleotideCorrelation) == 0) /
        dim(hs.promotersDinucleotideCorrelation)[1],
     sum(rowSums(hs.promotersDinucleotideCorrelation) == 1) /
        dim(hs.promotersDinucleotideCorrelation)[1],
```

```
    sum(rowSums(hs.promotersDinucleotideCorrelation) == 2) /
      dim(hs.promotersDinucleotideCorrelation)[1],
    sum(rowSums(hs.promotersDinucleotideCorrelation) == 3) /
      dim(hs.promotersDinucleotideCorrelation)[1],
    sum(rowSums(hs.promotersDinucleotideCorrelation) == 4) /
      dim(hs.promotersDinucleotideCorrelation)[1]
    )
)
```

And this is the code to repeat the same analysis on the other organisms:

First let read the MNase data:

```
# M. musculus:
mm.nucleosomes <- as.matrix(read.table('data/Mm_EPDnew_vs_GSM1293995.dat',
header=F, row.names=1)[rownames(mm.promoters.raw.ww),])
mm.m.nucleosomes <- matrix(nrow=dim(mm.nucleosomes)[1], ncol=201)
colnames(mm.m.nucleosomes) <- seq(-1000, 1000, 10)
rownames(mm.m.nucleosomes) <- rownames(mm.nucleosomes)
mm.m.nucleosomes <- cbind(mm.nucleosomes[,1], mm.nucleosomes,
mm.nucleosomes[,199])

# D. redio:
dr.nucleosomes <- as.matrix(read.table('data/Dr_EPDnew_vs_GSM1081554.dat',
header=F, row.names=1)[rownames(dr.promoters.raw.ww),])
colnames(dr.nucleosomes) <- -1000:1000
dr.m.nucleosomes <- matrix(nrow=dim(dr.nucleosomes)[1], ncol=201)
colnames(dr.m.nucleosomes) <- seq(-1000, 1000, 10)
rownames(dr.m.nucleosomes) <- rownames(dr.nucleosomes)

# this file is 1bp in steps, convert to a 10bp steps:
for (I in seq(-1000, 1000, 10)){
  if ( (I - 4) > -1000){
    gap.min <- I - 4
  }else{
    gap.min <- I
  }
  if ( (I + 5) < 1000){
    gap.max <- I + 5
  }else{
    gap.max <- I
  }
  gap <- as.character(gap.min:gap.max)
  dr.m.nucleosomes[,as.character(I)] <- rowSums(dr.nucleosomes[,gap],
na.rm=T)
}
```

```
# D. melanogaster:
dm.nucleosomes <- read.table('data/Dm_EPDnew_vs_GSM1248974.dat', header=F,
row.names=1)[rownames(dm.promoters.raw.ww),]
colnames(dm.nucleosomes) <- -1000:1000
dm.m.nucleosomes <- matrix(nrow=dim(dm.nucleosomes)[1], ncol=201)
colnames(dm.m.nucleosomes) <- seq(-1000, 1000, 10)
rownames(dm.m.nucleosomes) <- rownames(dm.nucleosomes)

# this file is 1bp in steps, convert to a 10bp steps:
for (I in seq(-1000, 1000, 10)){
  if ( (I - 4) > -1000){
    gap.min <- I - 4
  }else{
    gap.min <- I
  }
  if ( (I + 5) < 1000){
    gap.max <- I + 5
  }else{
    gap.max <- I
  }
  gap <- as.character(gap.min:gap.max)
  dm.m.nucleosomes[,as.character(I)] <- rowSums(dm.nucleosomes[,gap],
na.rm=T)
}

# C. elegans:
ce.nucleosomes <- as.matrix(read.table('data/Ce_EPDnew_vs_valouev08.out',
header=F, row.names=1)[rownames(ce.promoters.raw.ww),])
ce.m.nucleosomes <- cbind(ce.nucleosomes[,1], ce.nucleosomes,
ce.nucleosomes[,199])

### Save out the data:
save(hs.m.nucleosomes,
     mm.m.nucleosomes,
     dr.m.nucleosomes,
     dm.m.nucleosomes,
     ce.m.nucleosomes,
     file="data/nucleosomesDistributions.RData")
```

Then orer the promoters according to the average nucleosome distribution:

```
# M. musculus:
ordering.mm <- order(apply(mm.promoters.raw.ww, 1, cov,
colMeans(hs.m.nucleosomes)))
```

```
mm.ww <- mm.promoters.raw.ww[ordering.mm,]
mm.ww.mean <- condense.matrix(mm.ww, 20)

mm.nuc <- mm.m.nucleosomes[ordering.mm,]
mm.nuc.mean <- condense.matrix(mm.nuc, 20)

# D. rerio:
ordering.dr <- order(apply(dr.promoters.raw.ww, 1, cov,
colMeans(dr.m.nucleosomes)))

dr.ww <- dr.promoters.raw.ww[ordering.dr,]
dr.ww.mean <- condense.matrix(dr.ww, 10)

dr.nuc <- dr.m.nucleosomes[ordering.dr,]
dr.nuc.mean <- condense.matrix(dr.nuc, 10)

# D. melanogaster:
ordering.dm <- order(apply(dm.promoters.raw.ss , 1, cov,
colMeans(dm.m.nucleosomes)))

dm.ss <- dm.promoters.raw.ss[ordering.dm,]
dm.ss.mean <- condense.matrix(dm.ss, 10)

dm.nuc <- dm.m.nucleosomes[ordering.dm,]
dm.nuc.mean <- condense.matrix(dm.nuc, 10)

# C. elegans:
ordering.ce <- order(apply(ce.promoters.raw.ww, 1, cov,
colMeans(ce.m.nucleosomes)))

ce.ww <- ce.promoters.raw.ww[ordering.ce,]
ce.ww.mean <- condense.matrix(ce.ww, 5)

ce.nuc <- ce.m.nucleosomes[ordering.ce,]
ce.nuc.mean <- condense.matrix(ce.nuc, 5)
```

Get the number of promoters with concordant signal, their cumulative fraction and nucleosome distribution:

```
mm.average.yy.proxprom <- cbind(rowMeans(mm.promoters.raw.yy[,range.nfr],
     na.rm=T), rowMeans(mm.promoters.raw.yy[,range.1n], na.rm=T))
mm.average.rr.proxprom <- cbind(rowMeans(mm.promoters.raw.rr[,range.nfr],
     na.rm=T), rowMeans(mm.promoters.raw.rr[,range.1n], na.rm=T))
mm.average.ww.proxprom <- cbind(rowMeans(mm.promoters.raw.ww[,range.nfr],
     na.rm=T), rowMeans(mm.promoters.raw.ww[,range.1n], na.rm=T))
```

```
mm.average.ss.proxprom <- cbind(rowMeans(mm.promoters.raw.ss[,range.nfr],
     na.rm=T), rowMeans(mm.promoters.raw.ss[,range.1n], na.rm=T))

dm.average.yy.proxprom <- cbind(rowMeans(dm.promoters.raw.yy[,range.nfr],
     na.rm=T), rowMeans(dm.promoters.raw.yy[,range.1n], na.rm=T))
dm.average.rr.proxprom <- cbind(rowMeans(dm.promoters.raw.rr[,range.nfr],
     na.rm=T), rowMeans(dm.promoters.raw.rr[,range.1n], na.rm=T))
dm.average.ww.proxprom <- cbind(rowMeans(dm.promoters.raw.ww[,range.nfr],
     na.rm=T), rowMeans(dm.promoters.raw.ww[,range.1n], na.rm=T))
dm.average.ss.proxprom <- cbind(rowMeans(dm.promoters.raw.ss[,range.nfr],
     na.rm=T), rowMeans(dm.promoters.raw.ss[,range.1n], na.rm=T))

dr.average.yy.proxprom <- cbind(rowMeans(dr.promoters.raw.yy[,range.nfr],
     na.rm=T), rowMeans(dr.promoters.raw.yy[,range.1n], na.rm=T))
dr.average.rr.proxprom <- cbind(rowMeans(dr.promoters.raw.rr[,range.nfr],
     na.rm=T), rowMeans(dr.promoters.raw.rr[,range.1n], na.rm=T))
dr.average.ww.proxprom <- cbind(rowMeans(dr.promoters.raw.ww[,range.nfr],
     na.rm=T), rowMeans(dr.promoters.raw.ww[,range.1n], na.rm=T))
dr.average.ss.proxprom <- cbind(rowMeans(dr.promoters.raw.ss[,range.nfr],
     na.rm=T), rowMeans(dr.promoters.raw.ss[,range.1n], na.rm=T))

ce.average.yy.proxprom <- cbind(rowMeans(ce.promoters.raw.yy[,range.nfr],
     na.rm=T), rowMeans(ce.promoters.raw.yy[,range.1n], na.rm=T))
ce.average.rr.proxprom <- cbind(rowMeans(ce.promoters.raw.rr[,range.nfr],
     na.rm=T), rowMeans(ce.promoters.raw.rr[,range.1n], na.rm=T))
ce.average.ww.proxprom <- cbind(rowMeans(ce.promoters.raw.ww[,range.nfr],
     na.rm=T), rowMeans(ce.promoters.raw.ww[,range.1n], na.rm=T))
ce.average.ss.proxprom <- cbind(rowMeans(ce.promoters.raw.ss[,range.nfr],
     na.rm=T), rowMeans(ce.promoters.raw.ss[,range.1n], na.rm=T))

# make a matrix that summarize the promoters with concordant signal
mm.promotersDinucleotideCorrelation <- matrix(0, ncol=4,
     nrow=dim(mm.promoters.raw.ss)[1])
colnames(mm.promotersDinucleotideCorrelation) <- c("YY","RR","WW","SS")
rownames(mm.promotersDinucleotideCorrelation) <-
     rownames(mm.promoters.raw.ss)
mm.promotersDinucleotideCorrelation[which(mm.average.yy.proxprom[,2] >
     mean(mm.average.yy.proxprom)),1] <- 1
mm.promotersDinucleotideCorrelation[which(mm.average.rr.proxprom[,2] >
     mean(mm.average.rr.proxprom)),2] <- 1
mm.promotersDinucleotideCorrelation[which(mm.average.ww.proxprom[,2] >
     mean(mm.average.ww.proxprom)),3] <- 1
mm.promotersDinucleotideCorrelation[which(mm.average.ss.proxprom[,2] >
     mean(mm.average.ss.proxprom)),4] <- 1

dm.promotersDinucleotideCorrelation <- matrix(0, ncol=4,
     nrow=dim(dm.promoters.raw.ss)[1])
```

```
colnames(dm.promotersDinucleotideCorrelation) <- c("YY","RR","WW","SS")
rownames(dm.promotersDinucleotideCorrelation) <-
     rownames(dm.promoters.raw.ss)
dm.promotersDinucleotideCorrelation[which(dm.average.yy.proxprom[,2] >
     mean(dm.average.yy.proxprom)),1] <- 1
dm.promotersDinucleotideCorrelation[which(dm.average.rr.proxprom[,2] >
     mean(dm.average.rr.proxprom)),2] <- 1
dm.promotersDinucleotideCorrelation[which(dm.average.ww.proxprom[,2] >
     mean(dm.average.ww.proxprom)),3] <- 1
dm.promotersDinucleotideCorrelation[which(dm.average.ss.proxprom[,2] >
     mean(dm.average.ss.proxprom)),4] <- 1

dr.promotersDinucleotideCorrelation <- matrix(0, ncol=4,
     nrow=dim(dr.promoters.raw.ss)[1])
colnames(dr.promotersDinucleotideCorrelation) <- c("YY","RR","WW","SS")
rownames(dr.promotersDinucleotideCorrelation) <-
     rownames(dr.promoters.raw.ss)
dr.promotersDinucleotideCorrelation[which(dr.average.yy.proxprom[,2] >
     mean(dr.average.yy.proxprom)),1] <- 1
dr.promotersDinucleotideCorrelation[which(dr.average.rr.proxprom[,2] >
     mean(dr.average.rr.proxprom)),2] <- 1
dr.promotersDinucleotideCorrelation[which(dr.average.ww.proxprom[,2] >
     mean(dr.average.ww.proxprom)),3] <- 1
dr.promotersDinucleotideCorrelation[which(dr.average.ss.proxprom[,2] >
     mean(dr.average.ss.proxprom)),4] <- 1

ce.promotersDinucleotideCorrelation <- matrix(0, ncol=4,
     nrow=dim(ce.promoters.raw.ss)[1])
colnames(ce.promotersDinucleotideCorrelation) <- c("YY","RR","WW","SS")
rownames(ce.promotersDinucleotideCorrelation) <-
     rownames(ce.promoters.raw.ss)
ce.promotersDinucleotideCorrelation[which(ce.average.yy.proxprom[,2] >
     mean(ce.average.yy.proxprom)),1] <- 1
ce.promotersDinucleotideCorrelation[which(ce.average.rr.proxprom[,2] >
     mean(ce.average.rr.proxprom)),2] <- 1
ce.promotersDinucleotideCorrelation[which(ce.average.ww.proxprom[,2] >
     mean(ce.average.ww.proxprom)),3] <- 1
ce.promotersDinucleotideCorrelation[which(ce.average.ss.proxprom[,2] >
     mean(ce.average.ss.proxprom)),4] <- 1

# Calculate the fraction of promoters containing more that one concordant
# signal:
mm.fractionConcordanceDinucleotides <-
     cumsum(c(sum(rowSums(mm.promotersDinucleotideCorrelation) == 0) /
     dim(mm.promotersDinucleotideCorrelation)[1],

     sum(rowSums(mm.promotersDinucleotideCorrelation) == 1) /
```

```
     dim(mm.promotersDinucleotideCorrelation)[1],

     sum(rowSums(mm.promotersDinucleotideCorrelation) == 2) /
     dim(mm.promotersDinucleotideCorrelation)[1],

     sum(rowSums(mm.promotersDinucleotideCorrelation) == 3) /
     dim(mm.promotersDinucleotideCorrelation)[1],

     sum(rowSums(mm.promotersDinucleotideCorrelation) == 4) /
     dim(mm.promotersDinucleotideCorrelation)[1]))

dm.fractionConcordanceDinucleotides <-
     cumsum(c(sum(rowSums(dm.promotersDinucleotideCorrelation) == 0) /
     dim(dm.promotersDinucleotideCorrelation)[1],

     sum(rowSums(dm.promotersDinucleotideCorrelation) == 1) /
     dim(dm.promotersDinucleotideCorrelation)[1],

     sum(rowSums(dm.promotersDinucleotideCorrelation) == 2) /
     dim(dm.promotersDinucleotideCorrelation)[1],

     sum(rowSums(dm.promotersDinucleotideCorrelation) == 3) /
     dim(dm.promotersDinucleotideCorrelation)[1],

     sum(rowSums(dm.promotersDinucleotideCorrelation) == 4) /
     dim(dm.promotersDinucleotideCorrelation)[1]))

dr.fractionConcordanceDinucleotides <-
     cumsum(c(sum(rowSums(dr.promotersDinucleotideCorrelation) == 0) /
     dim(dr.promotersDinucleotideCorrelation)[1],

     sum(rowSums(dr.promotersDinucleotideCorrelation) == 1) /
     dim(dr.promotersDinucleotideCorrelation)[1],

     sum(rowSums(dr.promotersDinucleotideCorrelation) == 2) /
     dim(dr.promotersDinucleotideCorrelation)[1],

     sum(rowSums(dr.promotersDinucleotideCorrelation) == 3) /
     dim(dr.promotersDinucleotideCorrelation)[1],

     sum(rowSums(dr.promotersDinucleotideCorrelation) == 4) /
     dim(dr.promotersDinucleotideCorrelation)[1]))

ce.fractionConcordanceDinucleotides <-
     cumsum(c(sum(rowSums(ce.promotersDinucleotideCorrelation) == 0) /
     dim(ce.promotersDinucleotideCorrelation)[1],

     sum(rowSums(ce.promotersDinucleotideCorrelation) == 1) /
     dim(ce.promotersDinucleotideCorrelation)[1],
```

```
     sum(rowSums(ce.promotersDinucleotideCorrelation) == 2) /
     dim(ce.promotersDinucleotideCorrelation)[1],

     sum(rowSums(ce.promotersDinucleotideCorrelation) == 3) /
     dim(ce.promotersDinucleotideCorrelation)[1],

     sum(rowSums(ce.promotersDinucleotideCorrelation) == 4) /
     dim(ce.promotersDinucleotideCorrelation)[1]))
```

3.7. Map consensus sequence (10 bp motif) to *H. sapiens* promoters and evaluate the 10 bp frequency average

To map sequences to promoters and to find their pattern of occurrence the code needs 4 parts: (1) define the position weight matrix (PWM) for the consensus; (2) map the PWM to promoters; (3) use a Fourier transform to evaluate the 10 bp frequency for each promoters and (4) evaluate the average:

```
# Define motif YYWWNNRRSS as PWM:
#                    A  C  G  T
motif.1 <- rbind(
                 c(-1, 0,-1, 0), # Y
                 c(-1, 0,-1, 0), # Y
                 c( 0,-1,-1, 0), # W
                 c( 0,-1,-1, 0), # W
                 c( 0, 0, 0, 0), # N
                 c( 0, 0, 0, 0), # N
                 c( 0,-1, 0,-1), # R
                 c( 0,-1, 0,-1), # R
                 c(-1, 0, 0,-1), # S
                 c(-1, 0, 0,-1)  # S
                 )

# Map it to the promoters
hs.m1.matches <- matrix(ncol=2140, nrow=dim(hs.promoters)[1])
rownames(hs.m1.matches) <- rownames(hs.promoters)
for (K in 1:dim(hs.promoters)[1]){
  sequence.1 <- matrix(0,ncol=4, nrow=dim(hs.promoters)[2])
  sequence.1[which(hs.promoters[K,] == 1),1] <- 1
  sequence.1[which(hs.promoters[K,] == 2),2] <- 1
  sequence.1[which(hs.promoters[K,] == 3),3] <- 1
  sequence.1[which(hs.promoters[K,] == 4),4] <- 1
  rownames(sequence.1) <- colnames(hs.promoters)
  result <- vector()
  for (I in 1:(dim(sequence.1)[1]-dim(motif.1)[1])){
    pos <- as.numeric(rownames(sequence.1)[I+5])
    step <- I + dim(motif.1)[1] - 1
    pwmscan <- sum(sequence.1[I:step,] * motif.1)
    if (pwmscan > -3.5){ # 3 mis-matches
      find.m <- 1
```

```
    }else{
      find.m <- 0
    }
    result <- rbind(result, c(pos, find.m))
  }
  hs.m1.matches[K,] <- result[,2]
}
colnames(hs.m1.matches) <- result[,1]

plot(as.integer(colnames(hs.m1.matches)),colMeans(hs.m1.matches), type="l",
xlim=c(-100,300))

# Define intervals where evaluate NAS
intervals <- seq(-900, 900, 10)

# Evaluate the spectrum
hs.promoters.nas <- matrix(0, ncol=length(intervals),
nrow=dim(hs.m1.matches)[1])
colnames(hs.promoters.nas) <- intervals
rownames(hs.promoters.nas) <- rownames(hs.promoters)
for (I in intervals){
  region <- as.character(seq(I-74, I+75, 1))
  hs.region <- hs.m1.matches[,region]
  hs.region.nas <- apply(hs.region, 1, rawSpectraMappedSeq)
  cat(I, "\t", mean(hs.region.nas, na.rm=T), "\n")
  hs.promoters.nas[,which(intervals == I)] <- hs.region.nas
  plot(intervals, colMeans(hs.promoters.nas, na.rm=T), type="b", pch=19,
ylim=range(colMeans(hs.promoters.nas, na.rm=T)[colMeans(hs.promoters.nas,
na.rm=T)>0]))
}
```

3.8. Motif analysis of promoter and genomic nucleosomes

This section of the document want to investigate the consensus sequence generated by the 4 dinucleotides that is predominant in promoter nucleosomes and compares it to the genomic nucleosome consensus. Most of this analysis have been done using OPROF (http://ccg.vital-it.ch/ssa/oprof.php) for each of the 240 motifs (10 bp motifs generated permuting the dinucleotides WW, SS, YY, RR and 2 Ns) in the N+1 region of promoters and in genomic nucleosomes defined as MNase regions of 147 bp. Here is the code to read the OPROF results and to define two classes of sequences that are known to facilitate the wrapping of DNA around histones:

```
optimisedMotifs10bp <- read.table("data/optimisedMotifs10bp", header=T,
     row.names=1)
optimisedMotifs10bp.scaled <- t(t(optimisedMotifs10bp) /
     apply(optimisedMotifs10bp, 2, max))

# Now check the motifs found using MNase data
mnaseMotifs10bp <- read.table("data/mnaseMotifs10bp", header=T,
     row.names=1)
dr.mnaseMotifs10bp <- read.table("data/dr_mnaseMotifs10bp", header=T,
     row.names=1)
```

```
ce.mnaseMotifs10bp <- read.table("data/ce_mnaseMotifs10bp", header=T,
     row.names=1)
mm.mnaseMotifs10bp <- read.table("data/mm_mnaseMotifs10bp", header=T,
     row.names=1)

# merge them:
mnaseOptimisedMotifs <- cbind(mnaseMotifs10bp[,"H_sapiens"],
     mm.mnaseMotifs10bp, dr.mnaseMotifs10bp,
     mnaseMotifs10bp[,"D_melanogaster"], ce.mnaseMotifs10bp)
colnames(mnaseOptimisedMotifs)[c(1,4)] <- c("H_sapiens","D_melanogaster")
mnaseOptimisedMotifs.scaled <- t(t(mnaseOptimisedMotifs) /
     apply(mnaseOptimisedMotifs, 2, max))

# Define 2 groups of motifs:
ssyywwrr <- c("YYNWWRRNSS","SSYYNNWWRR","RRNSSNYYWW","SSNYYWWRRN",
              "WWRRSSNYYN","WWNNRRSSYY","WWNRRSSYYN","YYNWWNRRSS",
              "RRSSNYYNWW","YYWWNRRNSS","RRSSNYYWWN","WWNRRSSNYY",
              "SSYYNWWNRR","RRNSSYYNWW","SSYYNWWRRN","RRSSYYNWWN",
              "WWNRRNSSYY","WWRRNSSYYN","SSNYYNWWRR","WWRRNSSNYY",
              "RRSSYYNNWW","WWRRNNSSYY","RRSSNNYYWW","WWRRSSNNYY",
              "SSYYWWRRNN","YYWWNNRRSS","YYWWRRNNSS","SSNNYYWWRR",
              "RRSSYYWWNN","YYWWRRNSSN","YYWWRRSSNN","WWRRSSYYNN",
              "YYNNWWRRSS","RRNNSSYYWW","SSNYYWWNRR","SSYYWWNRRN",
              "YYNWWRRSSN","YYWWNRRSSN","SSYYWWNNRR","RRNSSYYWWN")

ssrrwwyy <- c("YYSSNNRRWW","SSRRWWNNYY","RRWWNYYSSN","WWNYYSSNRR",
              "WWNNYYSSRR","WWYYSSNNRR","RRWWYYSSNN","RRWWNYYNSS",
              "RRNWWYYSSN","YYSSRRWWNN","SSRRNWWNYY","RRNWWNYYSS",
              "SSNRRNWWYY","YYSSNRRWWN","RRWWNNYYSS","RRNYYNSSWW",
              "SSWWRRNYYN","WWNYYNSSRR","WWYYNNSSRR","YYNNSSRRWW",
              "SSRRWWNYYN","SSRRNWWYYN","WWYYNSSRRN","WWYYSSNRRN",
              "RRNWWYYNSS","SSRRNNWWYY","RRNNWWYYSS","YYNSSRRWWN",
              "YYSSNRRNWW","YYSSRRNNWW","YYNSSRRNWW","WWYYSSRRNN",
              "YYSSRRNWWN","WWYYNSSNRR","YYNSSNRRWW","WWNYYSSRRN",
              "RRWWYYNSSN","SSNRRWWYYN","SSNRRWWNYY","SSNNRRWWYY")

# and some representative to plot in the figure:
repres <- c("YYWWNNRRSS","WWNRRSSNYY","SSNYYWWNRR","RRSSNNYYWW",
            "SSNRRWWNYY","YYSSNNRRWW","WWNYYSSNRR","RRWWNNYYSS",
            "RRYYNWWSSN","YYNWWSSNRR","SSWWNNYYRR","WWSSNNRRYY",
            "YYRRNNSSWW","RRYYNNWWSS","SSWWNNRRYY","WWSSNNRRYY")
```

3.9. Analysis of consensus sequences in *C. elegans* promoters

This sub-section describes the correlation analysis to study the two consensus sequence families that are present in *C. elegans* promoters: the SS-RR-WW-YY family and the SS-YY-WW-RR family. One sequence is chosen as representative for each sequence.

The following is the code used to map the two sequences to C. elegans promoters with 3 MM. It is very similar to the code used to map a consensus sequence around *H. sapiens* promoters as presented in section 3.7.

```
# first motif: RRWWNNYYSS
motif.ce1 <- rbind(
                   c( 0,-1, 0,-1), # R
                   c( 0,-1, 0,-1), # R
                   c( 0,-1,-1, 0), # W
                   c( 0,-1,-1, 0), # W
                   c( 0, 0, 0, 0), # N
                   c( 0, 0, 0, 0), # N
                   c(-1, 0,-1, 0), # Y
                   c(-1, 0,-1, 0), # Y
                   c(-1, 0, 0,-1), # S
                   c(-1, 0, 0,-1)  # S
                   )

ce.tot.result.mce1.3 <- matrix(ncol=2140)
for (K in 1:dim(ce.promoters)[1]){
  sequence.1 <- matrix(0,ncol=4, nrow=dim(ce.promoters)[2])
  sequence.1[which(ce.promoters[K,] == 1),1] <- 1
  sequence.1[which(ce.promoters[K,] == 2),2] <- 1
  sequence.1[which(ce.promoters[K,] == 3),3] <- 1
  sequence.1[which(ce.promoters[K,] == 4),4] <- 1
  rownames(sequence.1) <- colnames(ce.promoters)
  result <- vector()
  for (I in 1:(dim(sequence.1)[1]-dim(motif.ce1)[1])){
    pos <- as.numeric(rownames(sequence.1)[I+5])
    step <- I + dim(motif.ce1)[1] - 1
    pwmscan <- sum(sequence.1[I:step,] * motif.ce1)
    if (pwmscan > -3.5){ # 3 MM
      find.m <- 1
    }else{
      find.m <- 0
    }
    result <- rbind(result, c(pos, find.m))
  }
  ce.tot.result.mce1.3 <- rbind(ce.tot.result.mce1.3, result[,2])
}

ce.tot.result.mce1.3 <- ce.tot.result.mce1.3[-1,]
colnames(ce.tot.result.mce1.3) <- result[,1]
plot(as.integer(colnames(ce.tot.result.mce1.3)),colMeans(ce.tot.result.mce1
       .3), type="l", xlim=c(-100,300), col="magenta")

# See how this signal is in single promoter:
intervals <- seq(-900,900,10)
ce.promoters.mce1.3 <- matrix(0, ncol=length(intervals),
```

```
       nrow=dim(ce.promoters)[1])
colnames(ce.promoters.mce1.3) <- intervals
rownames(ce.promoters.mce1.3) <- rownames(ce.promoters)
for (I in intervals){
  region <- as.character(seq(I-74, I+75, 1))
  ce.region <- ce.tot.result.mce1.3[,region]
  ce.region.raw <- apply(ce.region, 1, rawSpectraMappedSeq, freq=10)
  cat(I, "\t", mean(ce.region.raw, na.rm=T), "\n")
  ce.promoters.mce1.3[,which(intervals == I)] <- ce.region.raw
  plot(intervals, colMeans(ce.promoters.mce1.3, na.rm=T), type="b", pch=19,
       ylim=range(colMeans(ce.promoters.mce1.3,
       na.rm=T)[colMeans(ce.promoters.mce1.3, na.rm=T)>0]))
}

# second motif: YYWWNNNRRSS
motif.ce2 <- rbind(
                   c(-1, 0,-1, 0), # Y
                   c(-1, 0,-1, 0), # Y
                   c( 0,-1,-1, 0), # W
                   c( 0,-1,-1, 0), # W
                   c( 0, 0, 0, 0), # N
                   c( 0, 0, 0, 0), # N
                   c( 0,-1, 0,-1), # R
                   c( 0,-1, 0,-1), # R
                   c(-1, 0, 0,-1), # S
                   c(-1, 0, 0,-1)  # S
                   )

ce.tot.result.mce2.3 <- matrix(ncol=2140)
for (K in 1:dim(ce.promoters)[1]){
  sequence.1 <- matrix(0,ncol=4, nrow=dim(ce.promoters)[2])
  sequence.1[which(ce.promoters[K,] == 1),1] <- 1
  sequence.1[which(ce.promoters[K,] == 2),2] <- 1
  sequence.1[which(ce.promoters[K,] == 3),3] <- 1
  sequence.1[which(ce.promoters[K,] == 4),4] <- 1
  rownames(sequence.1) <- colnames(ce.promoters)
  result <- vector()
  for (I in 1:(dim(sequence.1)[1]-dim(motif.ce2)[1])){
    pos <- as.numeric(rownames(sequence.1)[I+5])
    step <- I + dim(motif.ce2)[1] - 1
    pwmscan <- sum(sequence.1[I:step,] * motif.ce2)
    if (pwmscan > -3.5){ # 3 MM
      find.m <- 1
    }else{
      find.m <- 0
    }
    result <- rbind(result, c(pos, find.m))
  }
  ce.tot.result.mce2.3 <- rbind(ce.tot.result.mce2.3, result[,2])
```

```
}

ce.tot.result.mce2.3 <- ce.tot.result.mce2.3[-1,]
colnames(ce.tot.result.mce2.3) <- result[,1]
plot(as.integer(colnames(ce.tot.result.mce2.3)),colMeans(ce.tot.result.mce2
       .3), type="l", xlim=c(-100,300), col="magenta")

# See how this signal is in single promoter:
intervals <- seq(-900,900,10)
ce.promoters.mce2.3 <- matrix(0, ncol=length(intervals),
       nrow=dim(ce.promoters)[1])
colnames(ce.promoters.mce2.3) <- intervals
rownames(ce.promoters.mce2.3) <- rownames(ce.promoters)
for (I in intervals){
  region <- as.character(seq(I-74, I+75, 1))
  ce.region <- ce.tot.result.mce2.3[,region]
  ce.region.raw <- apply(ce.region, 1, rawSpectraMappedSeq, freq=10)
  cat(I, "\t", mean(ce.region.raw, na.rm=T), "\n")
  ce.promoters.mce2.3[,which(intervals == I)] <- ce.region.raw
  plot(intervals, colMeans(ce.promoters.mce2.3, na.rm=T), type="b", pch=19,
       ylim=range(colMeans(ce.promoters.mce2.3,
       na.rm=T)[colMeans(ce.promoters.mce2.3, na.rm=T)>0]))
}
```

As before for the *H. sapiens* consensus sequence analysis, here the result give two matrices with the 10 bp frequency intensity of the consensus sequence around each promoter evaluated using a Fourier transform. With 3 MM the two consensus sequences are quite degenerated and could potentially bind the same sequence resulting in some ovlap in the signal intensity. To be sure that this is not the case, repeat the analysis reducing the number of allowed MM to 2 and then to 1:

```
####
### Now reduce the MM to 2:

# First motif
ce.tot.result.mce1.2 <- matrix(ncol=2140)
for (K in 1:dim(ce.promoters)[1]){
  sequence.1 <- matrix(0,ncol=4, nrow=dim(ce.promoters)[2])
  sequence.1[which(ce.promoters[K,] == 1),1] <- 1
  sequence.1[which(ce.promoters[K,] == 2),2] <- 1
  sequence.1[which(ce.promoters[K,] == 3),3] <- 1
  sequence.1[which(ce.promoters[K,] == 4),4] <- 1
  rownames(sequence.1) <- colnames(ce.promoters)
  result <- vector()
  for (I in 1:(dim(sequence.1)[1]-dim(motif.ce1)[1])){
    pos <- as.numeric(rownames(sequence.1)[I+5])
    step <- I + dim(motif.ce1)[1] - 1
    pwmscan <- sum(sequence.1[I:step,] * motif.ce1)
    if (pwmscan > -2.5){ # 2 MM
      find.m <- 1
    }else{
```

```
      find.m <- 0
    }
    result <- rbind(result, c(pos, find.m))
  }
  ce.tot.result.mce1.2 <- rbind(ce.tot.result.mce1.2, result[,2])
}

ce.tot.result.mce1.2 <- ce.tot.result.mce1.2[-1,]
colnames(ce.tot.result.mce1.2) <- result[,1]
plot(as.integer(colnames(ce.tot.result.mce1.2)),colMeans(ce.tot.result.mce1
       .2), type="l", xlim=c(-100,300), col="red")

intervals <- seq(-900,900,10)
ce.promoters.mce1.2 <- matrix(0, ncol=length(intervals),
       nrow=dim(ce.promoters)[1])
colnames(ce.promoters.mce1.2) <- intervals
rownames(ce.promoters.mce1.2) <- rownames(ce.promoters)
for (I in intervals){
  region <- as.character(seq(I-74, I+75, 1))
  ce.region <- ce.tot.result.mce1.2[,region]
  ce.region.raw <- apply(ce.region, 1, rawSpectraMappedSeq, freq=10)
  cat(I, "\t", mean(ce.region.raw, na.rm=T), "\n")
  ce.promoters.mce1.2[,which(intervals == I)] <- ce.region.raw
  plot(intervals, colMeans(ce.promoters.mce1.2, na.rm=T), type="b", pch=19,
       ylim=range(colMeans(ce.promoters.mce1.2,
       na.rm=T)[colMeans(ce.promoters.mce1.2, na.rm=T)>0]))
}

# Second motif
ce.tot.result.mce2.2 <- matrix(ncol=2140)
for (K in 1:dim(ce.promoters)[1]){
  sequence.1 <- matrix(0,ncol=4, nrow=dim(ce.promoters)[2])
  sequence.1[which(ce.promoters[K,] == 1),1] <- 1
  sequence.1[which(ce.promoters[K,] == 2),2] <- 1
  sequence.1[which(ce.promoters[K,] == 3),3] <- 1
  sequence.1[which(ce.promoters[K,] == 4),4] <- 1
  rownames(sequence.1) <- colnames(ce.promoters)
  result <- vector()
  for (I in 1:(dim(sequence.1)[1]-dim(motif.ce2)[1])){
    pos <- as.numeric(rownames(sequence.1)[I+5])
    step <- I + dim(motif.ce2)[1] - 1
    pwmscan <- sum(sequence.1[I:step,] * motif.ce2)
    if (pwmscan > -2.5){ # 2 MM
      find.m <- 1
    }else{
      find.m <- 0
    }
    result <- rbind(result, c(pos, find.m))
  }
```

```
  ce.tot.result.mce2.2 <- rbind(ce.tot.result.mce2.2, result[,2])
}

ce.tot.result.mce2.2 <- ce.tot.result.mce2.2[-1,]
colnames(ce.tot.result.mce2.2) <- result[,1]
plot(as.integer(colnames(ce.tot.result.mce2.2)),colMeans(ce.tot.result.mce2
       .2), type="l", xlim=c(-100,300), col="red")

intervals <- seq(-900,900,10)
ce.promoters.mce2.2 <- matrix(0, ncol=length(intervals),
       nrow=dim(ce.promoters)[1])
colnames(ce.promoters.mce2.2) <- intervals
rownames(ce.promoters.mce2.2) <- rownames(ce.promoters)
for (I in intervals){
  region <- as.character(seq(I-74, I+75, 1))
  ce.region <- ce.tot.result.mce2.2[,region]
  ce.region.raw <- apply(ce.region, 1, rawSpectraMappedSeq, freq=10)
  cat(I, "\t", mean(ce.region.raw, na.rm=T), "\n")
  ce.promoters.mce2.2[,which(intervals == I)] <- ce.region.raw
  plot(intervals, colMeans(ce.promoters.mce2.2, na.rm=T), type="b", pch=19,
       ylim=range(colMeans(ce.promoters.mce2.2,
       na.rm=T)[colMeans(ce.promoters.mce2.2, na.rm=T)>0]))
}
```

And now with 1 MM:

```
####
### Now reduce the MM to 1:

# First motif
ce.tot.result.mce1.1 <- matrix(ncol=2140)
for (K in 1:dim(ce.promoters)[1]){
  sequence.1 <- matrix(0,ncol=4, nrow=dim(ce.promoters)[2])
  sequence.1[which(ce.promoters[K,] == 1),1] <- 1
  sequence.1[which(ce.promoters[K,] == 2),2] <- 1
  sequence.1[which(ce.promoters[K,] == 3),3] <- 1
  sequence.1[which(ce.promoters[K,] == 4),4] <- 1
  rownames(sequence.1) <- colnames(ce.promoters)
  result <- vector()
  for (I in 1:(dim(sequence.1)[1]-dim(motif.ce1)[1])){
    pos <- as.numeric(rownames(sequence.1)[I+5])
    step <- I + dim(motif.ce1)[1] - 1
    pwmscan <- sum(sequence.1[I:step,] * motif.ce1)
    if (pwmscan > -1.5){ # 1 MM
      find.m <- 1
    }else{
      find.m <- 0
    }
    result <- rbind(result, c(pos, find.m))
  }
```

```
  ce.tot.result.mce1.1 <- rbind(ce.tot.result.mce1.1, result[,2])
}

ce.tot.result.mce1.1 <- ce.tot.result.mce1.1[-1,]
colnames(ce.tot.result.mce1.1) <- result[,1]
plot(as.integer(colnames(ce.tot.result.mce1.1)),colMeans(ce.tot.result.mce1
.1), type="l", xlim=c(-100,300), col="red")

intervals <- seq(-900,900,10)
ce.promoters.mce1.1 <- matrix(0, ncol=length(intervals),
nrow=dim(ce.promoters)[1])
colnames(ce.promoters.mce1.1) <- intervals
rownames(ce.promoters.mce1.1) <- rownames(ce.promoters)
for (I in intervals){
  region <- as.character(seq(I-74, I+75, 1))
  ce.region <- ce.tot.result.mce1.1[,region]
  ce.region.raw <- apply(ce.region, 1, rawSpectraMappedSeq, freq=10)
  cat(I, "\t", mean(ce.region.raw, na.rm=T), "\n")
  ce.promoters.mce1.1[,which(intervals == I)] <- ce.region.raw
  plot(intervals, colMeans(ce.promoters.mce1.1, na.rm=T), type="b", pch=19,
ylim=range(colMeans(ce.promoters.mce1.1,
na.rm=T)[colMeans(ce.promoters.mce1.1, na.rm=T)>0]))
}

# Second motif
ce.tot.result.mce2.1 <- matrix(ncol=2140)
for (K in 1:dim(ce.promoters)[1]){
  sequence.1 <- matrix(0,ncol=4, nrow=dim(ce.promoters)[2])
  sequence.1[which(ce.promoters[K,] == 1),1] <- 1
  sequence.1[which(ce.promoters[K,] == 2),2] <- 1
  sequence.1[which(ce.promoters[K,] == 3),3] <- 1
  sequence.1[which(ce.promoters[K,] == 4),4] <- 1
  rownames(sequence.1) <- colnames(ce.promoters)
  result <- vector()
  for (I in 1:(dim(sequence.1)[1]-dim(motif.ce2)[1])){
    pos <- as.numeric(rownames(sequence.1)[I+5])
    step <- I + dim(motif.ce2)[1] - 1
    pwmscan <- sum(sequence.1[I:step,] * motif.ce2)
    if (pwmscan > -1.5){ # 1 MM
      find.m <- 1
    }else{
      find.m <- 0
    }
    result <- rbind(result, c(pos, find.m))
  }
  ce.tot.result.mce2.1 <- rbind(ce.tot.result.mce2.1, result[,2])
}

ce.tot.result.mce2.1 <- ce.tot.result.mce2.1[-1,]
```

```
colnames(ce.tot.result.mce2.1) <- result[,1]
plot(as.integer(colnames(ce.tot.result.mce2.1)),colMeans(ce.tot.result.mce2
.1), type="l", xlim=c(-100,300), col="red")

intervals <- seq(-900,900,10)
ce.promoters.mce2.1 <- matrix(0, ncol=length(intervals),
nrow=dim(ce.promoters)[1])
colnames(ce.promoters.mce2.1) <- intervals
rownames(ce.promoters.mce2.1) <- rownames(ce.promoters)
for (I in intervals){
  region <- as.character(seq(I-74, I+75, 1))
  ce.region <- ce.tot.result.mce2.1[,region]
  ce.region.raw <- apply(ce.region, 1, rawSpectraMappedSeq, freq=10)
  cat(I, "\t", mean(ce.region.raw, na.rm=T), "\n")
  ce.promoters.mce2.1[,which(intervals == I)] <- ce.region.raw
  plot(intervals, colMeans(ce.promoters.mce2.1, na.rm=T), type="b", pch=19,
ylim=range(colMeans(ce.promoters.mce2.1,
na.rm=T)[colMeans(ce.promoters.mce2.1, na.rm=T)>0]))
}
```

Extract promoters that are characterize for a strong signal in only one of the consensus:

```
# Get promoters with one or the other motif signal in the N+1 region:
ce.promoters.with.mce1 <-
     which(log2(rowMeans(ce.promoters.mce1.3[,as.character(seq(100,150,10))
     ])/rowMeans(ce.promoters.mce2.3[,as.character(seq(100,150,10))])) > 1
     & rowMeans(ce.promoters.mce1.3[,as.character(seq(100,150,10))]) > 1)
ce.promoters.with.mce2 <-
     which(log2(rowMeans(ce.promoters.mce1.3[,as.character(seq(100,150,10))
     ])/rowMeans(ce.promoters.mce2.3[,as.character(seq(100,150,10))])) < -1
     & rowMeans(ce.promoters.mce2.3[,as.character(seq(100,150,10))]) > 1)

# as expected motif 1 is more frequent in promoters:
length(ce.promoters.with.mce1)
length(ce.promoters.with.mce2)
```

3.10. Correlation analysis between DI and nucleosome affinity in the N+1 region

This subsection describes the steps to be taken to calculate the correlation between Pol-II fuzziness in starting transcription (defined by the Dispersion Index) and nucleosome affinity in the N+1 region definded as the sum of the 10 bp frequency intensities for the 4 dinucleotides. DI is a measure of dispersion that can be considered similar to the standard deviation aound the most probable initiation site. For each organism, the values have been calculated for each promoter and for each CAGE sample and store in a file. This file is the input for this section. Later on in the document you will lern how to do it for a small group of samples.

First, get the DI values for *H. sapiens* (then this part will be repeated for all other organisms):

```
DI.promoters.encode <-
     read.table("data/cage_cell_longPolyA_dispersion.dat.gz", header=T,
     row.names=1)
DI.promoters.fantom <- read.table("data/hsCageDispersion.dat.gz",
```

```
     row.names=1)
DI.promoters <- cbind(DI.promoters.encode, DI.promoters.fantom)
DI.promoters[DI.promoters == -1] <- NA
DI.promoters[DI.promoters == 0] <- NA
DI.promoters.median <- apply(DI.promoters, 1, mean, na.rm=T)

plot(density(DI.promoters.median, na.rm=T))
```

Get the average dinucleotide signal for the N+1 region and divide promoters according to their TATA-box status and DI:

```
# For each promoter get the average of the dinucleotide intensity in the
# NFR and N+1
average.yy.proxprom <- cbind(rowMeans(hs.promoters.raw.yy[,range.nfr],
     na.rm=T), rowMeans(hs.promoters.raw.yy[,range.1n], na.rm=T))
average.rr.proxprom <- cbind(rowMeans(hs.promoters.raw.rr[,range.nfr],
     na.rm=T), rowMeans(hs.promoters.raw.rr[,range.1n], na.rm=T))
average.ww.proxprom <- cbind(rowMeans(hs.promoters.raw.ww[,range.nfr],
     na.rm=T), rowMeans(hs.promoters.raw.ww[,range.1n], na.rm=T))
average.ss.proxprom <- cbind(rowMeans(hs.promoters.raw.ss[,range.nfr],
     na.rm=T), rowMeans(hs.promoters.raw.ss[,range.1n], na.rm=T))

# Get the average intensity for the N+1 in TATA-box promoters:
average.ww.di.tata <- mean(average.ww.proxprom[hs.tata.promoters,2],
     na.rm=T)
average.rr.di.tata <- mean(average.rr.proxprom[hs.tata.promoters,2],
     na.rm=T)
average.yy.di.tata <- mean(average.yy.proxprom[hs.tata.promoters,2],
     na.rm=T)
average.ss.di.tata <- mean(average.ss.proxprom[hs.tata.promoters,2],
     na.rm=T)
average.dinucleotite.tata <- mean(c(average.ww.di.tata, average.rr.di.tata,
     average.yy.di.tata, average.ss.di.tata))

# And their DI
average.di.tata <- mean(DI.promoters.median[hs.tata.promoters], na.rm=T)

# DI for TATA-less promoters
tataless.prom <- rownames(hs.promoterAnnotation[hs.promoterAnnotation[,1]
     == 0,])
# order the TATA-less promoters according to their DI
ordered.DI <- names(sort(DI.promoters.median[tataless.prom]))

# Get the average dinucleotide intensity for TATA-less promoters ordered
# by their DI in groups of 2000:
average.ww.di <- c(mean(average.ww.proxprom[ordered.DI[1:2000],2]),
                   mean(average.ww.proxprom[ordered.DI[2001:4000],2]),
```

```
                   mean(average.ww.proxprom[ordered.DI[4001:6000],2]),
                   mean(average.ww.proxprom[ordered.DI[6001:8000],2]),
                   mean(average.ww.proxprom[ordered.DI[8001:10000],2]),
                   mean(average.ww.proxprom[ordered.DI[10001:12000],2]),
                   mean(average.ww.proxprom[ordered.DI[12001:14000],2]),
                   mean(average.ww.proxprom[ordered.DI[14001:16000],2]),
                   mean(average.ww.proxprom[ordered.DI[16001:18000],2]),
                   mean(average.ww.proxprom[ordered.DI[18001:20000],2]),
                   mean(average.ww.proxprom[ordered.DI[20001:22000],2]),
                   mean(average.ww.proxprom[ordered.DI[22001:23932],2]))

average.ss.di <- c(mean(average.ss.proxprom[ordered.DI[1:2000],2]),
                   mean(average.ss.proxprom[ordered.DI[2001:4000],2]),
                   mean(average.ss.proxprom[ordered.DI[4001:6000],2]),
                   mean(average.ss.proxprom[ordered.DI[6001:8000],2]),
                   mean(average.ss.proxprom[ordered.DI[8001:10000],2]),
                   mean(average.ss.proxprom[ordered.DI[10001:12000],2]),
                   mean(average.ss.proxprom[ordered.DI[12001:14000],2]),
                   mean(average.ss.proxprom[ordered.DI[14001:16000],2]),
                   mean(average.ss.proxprom[ordered.DI[16001:18000],2]),
                   mean(average.ss.proxprom[ordered.DI[18001:20000],2]),
                   mean(average.ss.proxprom[ordered.DI[20001:22000],2]),
                   mean(average.ss.proxprom[ordered.DI[22001:23932],2]))

average.yy.di <- c(mean(average.yy.proxprom[ordered.DI[1:2000],2]),
                   mean(average.yy.proxprom[ordered.DI[2001:4000],2]),
                   mean(average.yy.proxprom[ordered.DI[4001:6000],2]),
                   mean(average.yy.proxprom[ordered.DI[6001:8000],2]),
                   mean(average.yy.proxprom[ordered.DI[8001:10000],2]),
                   mean(average.yy.proxprom[ordered.DI[10001:12000],2]),
                   mean(average.yy.proxprom[ordered.DI[12001:14000],2]),
                   mean(average.yy.proxprom[ordered.DI[14001:16000],2]),
                   mean(average.yy.proxprom[ordered.DI[16001:18000],2]),
                   mean(average.yy.proxprom[ordered.DI[18001:20000],2]),
                   mean(average.yy.proxprom[ordered.DI[20001:22000],2]),
                   mean(average.yy.proxprom[ordered.DI[22001:23932],2]))

average.rr.di <- c(mean(average.rr.proxprom[ordered.DI[1:2000],2]),
                   mean(average.rr.proxprom[ordered.DI[2001:4000],2]),
                   mean(average.rr.proxprom[ordered.DI[4001:6000],2]),
                   mean(average.rr.proxprom[ordered.DI[6001:8000],2]),
                   mean(average.rr.proxprom[ordered.DI[8001:10000],2]),
                   mean(average.rr.proxprom[ordered.DI[10001:12000],2]),
```

```
                   mean(average.rr.proxprom[ordered.DI[12001:14000],2]),
                   mean(average.rr.proxprom[ordered.DI[14001:16000],2]),
                   mean(average.rr.proxprom[ordered.DI[16001:18000],2]),
                   mean(average.rr.proxprom[ordered.DI[18001:20000],2]),
                   mean(average.rr.proxprom[ordered.DI[20001:22000],2]),
                   mean(average.rr.proxprom[ordered.DI[22001:23932],2]))

# evaluate also the average DI for the same groups
average.di <- c(mean(DI.promoters.median[ordered.DI[1:2000]]),
                mean(DI.promoters.median[ordered.DI[2001:4000]]),
                mean(DI.promoters.median[ordered.DI[4001:6000]]),
                mean(DI.promoters.median[ordered.DI[6001:8000]]),
                mean(DI.promoters.median[ordered.DI[8001:10000]]),
                mean(DI.promoters.median[ordered.DI[10001:12000]]),
                mean(DI.promoters.median[ordered.DI[12001:14000]]),
                mean(DI.promoters.median[ordered.DI[14001:16000]]),
                mean(DI.promoters.median[ordered.DI[16001:18000]]),
                mean(DI.promoters.median[ordered.DI[18001:20000]]),
                mean(DI.promoters.median[ordered.DI[20001:22000]]),
                mean(DI.promoters.median[ordered.DI[22001:23932]],
     na.rm=T))

# get the global average
average.dinucleotide.di <- cbind(average.ww.di, average.yy.di,
     average.ss.di, average.rr.di)
all.dinucleotide.di <- apply(average.dinucleotide.di,1,mean)

# check the correlation:
cor(all.dinucleotide.di, average.di)
summary(lm(all.dinucleotide.di ~ average.di))
```

Now, the same analysis is repeated for the other organisms:

```
## M. musculus
# Get promoters DI
mm.DI.promoters <- read.table("data/mmCageDispersion.dat.gz", row.names=1)
mm.DI.promoters[mm.DI.promoters == -1] <- NA
mm.DI.promoters[mm.DI.promoters == 0] <- NA
mm.DI.promoters.median <- apply(mm.DI.promoters, 1, mean, na.rm=T)
plot(density(mm.DI.promoters.median, na.rm=T))

# Get average raw signals for promoters:
mm.average.yy.proxprom <- cbind(rowMeans(mm.promoters.raw.yy[,range.nfr], na.rm=T),
   rowMeans(mm.promoters.raw.yy[,range.1n], na.rm=T))
mm.average.rr.proxprom <- cbind(rowMeans(mm.promoters.raw.rr[,range.nfr], na.rm=T),
   rowMeans(mm.promoters.raw.rr[,range.1n], na.rm=T))
```

```
mm.average.ww.proxprom <- cbind(rowMeans(mm.promoters.raw.ww[,range.nfr], na.rm=T),
   rowMeans(mm.promoters.raw.ww[,range.1n], na.rm=T))
mm.average.ss.proxprom <- cbind(rowMeans(mm.promoters.raw.ss[,range.nfr], na.rm=T),
   rowMeans(mm.promoters.raw.ss[,range.1n], na.rm=T))

mm.average.ww.di.tata <- mean(mm.average.ww.proxprom[mm.tata.promoters,2], na.rm=T)
mm.average.rr.di.tata <- mean(mm.average.rr.proxprom[mm.tata.promoters,2], na.rm=T)
mm.average.yy.di.tata <- mean(mm.average.yy.proxprom[mm.tata.promoters,2], na.rm=T)
mm.average.ss.di.tata <- mean(mm.average.ss.proxprom[mm.tata.promoters,2], na.rm=T)
mm.average.dinucleotite.tata <- mean(c(mm.average.ww.di.tata, mm.average.ss.di.tata,
   mm.average.yy.di.tata, mm.average.ss.di.tata))

mm.average.di.tata <- mean(mm.DI.promoters.median[mm.tata.promoters], na.rm=T)

mm.tataless <- rownames(mm.promoterAnnotation[mm.promoterAnnotation[,1] == 0,])
mm.ordered.DI <- names(sort(mm.DI.promoters.median[mm.tataless]))

g.intervals <- cbind(seq(1, length(mm.ordered.DI), 2000), c(seq(2000, length(mm.ordered.DI),
   2000), length(mm.ordered.DI)))

mm.average.ww.di <-
   c(mean(mm.average.ww.proxprom[mm.ordered.DI[g.intervals[1,1]:g.intervals[1,2]],2]),
   mean(mm.average.ww.proxprom[mm.ordered.DI[g.intervals[2,1]:g.intervals[2,2]],2]),
   mean(mm.average.ww.proxprom[mm.ordered.DI[g.intervals[3,1]:g.intervals[3,2]],2]),
   mean(mm.average.ww.proxprom[mm.ordered.DI[g.intervals[4,1]:g.intervals[4,2]],2]),
   mean(mm.average.ww.proxprom[mm.ordered.DI[g.intervals[5,1]:g.intervals[5,2]],2]),
   mean(mm.average.ww.proxprom[mm.ordered.DI[g.intervals[6,1]:g.intervals[6,2]],2]),
   mean(mm.average.ww.proxprom[mm.ordered.DI[g.intervals[7,1]:g.intervals[7,2]],2]),
   mean(mm.average.ww.proxprom[mm.ordered.DI[g.intervals[8,1]:g.intervals[8,2]],2]),
   mean(mm.average.ww.proxprom[mm.ordered.DI[g.intervals[9,1]:g.intervals[9,2]],2]),
   mean(mm.average.ww.proxprom[mm.ordered.DI[g.intervals[10,1]:g.intervals[10,2]],2]))

mm.average.ss.di <-
   c(mean(mm.average.ss.proxprom[mm.ordered.DI[g.intervals[1,1]:g.intervals[1,2]],2]),
   mean(mm.average.ss.proxprom[mm.ordered.DI[g.intervals[2,1]:g.intervals[2,2]],2]),
   mean(mm.average.ss.proxprom[mm.ordered.DI[g.intervals[3,1]:g.intervals[3,2]],2]),
   mean(mm.average.ss.proxprom[mm.ordered.DI[g.intervals[4,1]:g.intervals[4,2]],2]),
   mean(mm.average.ss.proxprom[mm.ordered.DI[g.intervals[5,1]:g.intervals[5,2]],2]),
   mean(mm.average.ss.proxprom[mm.ordered.DI[g.intervals[6,1]:g.intervals[6,2]],2]),
   mean(mm.average.ss.proxprom[mm.ordered.DI[g.intervals[7,1]:g.intervals[7,2]],2]),
   mean(mm.average.ss.proxprom[mm.ordered.DI[g.intervals[8,1]:g.intervals[8,2]],2]),
   mean(mm.average.ss.proxprom[mm.ordered.DI[g.intervals[9,1]:g.intervals[9,2]],2]),
   mean(mm.average.ss.proxprom[mm.ordered.DI[g.intervals[10,1]:g.intervals[10,2]],2]))

mm.average.yy.di <-
   c(mean(mm.average.yy.proxprom[mm.ordered.DI[g.intervals[1,1]:g.intervals[1,2]],2]),
   mean(mm.average.yy.proxprom[mm.ordered.DI[g.intervals[2,1]:g.intervals[2,2]],2]),
   mean(mm.average.yy.proxprom[mm.ordered.DI[g.intervals[3,1]:g.intervals[3,2]],2]),
   mean(mm.average.yy.proxprom[mm.ordered.DI[g.intervals[4,1]:g.intervals[4,2]],2]),
   mean(mm.average.yy.proxprom[mm.ordered.DI[g.intervals[5,1]:g.intervals[5,2]],2]),
   mean(mm.average.yy.proxprom[mm.ordered.DI[g.intervals[6,1]:g.intervals[6,2]],2]),
   mean(mm.average.yy.proxprom[mm.ordered.DI[g.intervals[7,1]:g.intervals[7,2]],2]),
   mean(mm.average.yy.proxprom[mm.ordered.DI[g.intervals[8,1]:g.intervals[8,2]],2]),
   mean(mm.average.yy.proxprom[mm.ordered.DI[g.intervals[9,1]:g.intervals[9,2]],2]),
```

```
   mean(mm.average.yy.proxprom[mm.ordered.DI[g.intervals[10,1]:g.intervals[10,2]],2]))

mm.average.rr.di <-
   c(mean(mm.average.rr.proxprom[mm.ordered.DI[g.intervals[1,1]:g.intervals[1,2]],2]),
   mean(mm.average.rr.proxprom[mm.ordered.DI[g.intervals[2,1]:g.intervals[2,2]],2]),
   mean(mm.average.rr.proxprom[mm.ordered.DI[g.intervals[3,1]:g.intervals[3,2]],2]),
   mean(mm.average.rr.proxprom[mm.ordered.DI[g.intervals[4,1]:g.intervals[4,2]],2]),
   mean(mm.average.rr.proxprom[mm.ordered.DI[g.intervals[5,1]:g.intervals[5,2]],2]),
   mean(mm.average.rr.proxprom[mm.ordered.DI[g.intervals[6,1]:g.intervals[6,2]],2]),
   mean(mm.average.rr.proxprom[mm.ordered.DI[g.intervals[7,1]:g.intervals[7,2]],2]),
   mean(mm.average.rr.proxprom[mm.ordered.DI[g.intervals[8,1]:g.intervals[8,2]],2]),
   mean(mm.average.rr.proxprom[mm.ordered.DI[g.intervals[9,1]:g.intervals[9,2]],2]),
   mean(mm.average.rr.proxprom[mm.ordered.DI[g.intervals[10,1]:g.intervals[10,2]],2]))

mm.average.di <-
   c(mean(mm.DI.promoters.median[mm.ordered.DI[g.intervals[1,1]:g.intervals[1,2]]]),
   mean(mm.DI.promoters.median[mm.ordered.DI[g.intervals[2,1]:g.intervals[2,2]]]),
   mean(mm.DI.promoters.median[mm.ordered.DI[g.intervals[3,1]:g.intervals[3,2]]]),
   mean(mm.DI.promoters.median[mm.ordered.DI[g.intervals[4,1]:g.intervals[4,2]]]),
   mean(mm.DI.promoters.median[mm.ordered.DI[g.intervals[5,1]:g.intervals[5,2]]]),
   mean(mm.DI.promoters.median[mm.ordered.DI[g.intervals[6,1]:g.intervals[6,2]]]),
   mean(mm.DI.promoters.median[mm.ordered.DI[g.intervals[7,1]:g.intervals[7,2]]]),
   mean(mm.DI.promoters.median[mm.ordered.DI[g.intervals[8,1]:g.intervals[8,2]]]),
   mean(mm.DI.promoters.median[mm.ordered.DI[g.intervals[9,1]:g.intervals[9,2]]]),
   mean(mm.DI.promoters.median[mm.ordered.DI[g.intervals[10,1]:g.intervals[10,2]]]))

mm.average.dinucleotide.di <- cbind(mm.average.ww.di, mm.average.yy.di, mm.average.ss.di,
   mm.average.rr.di)
mm.all.dinucleotide.di <- apply(mm.average.dinucleotide.di,1,mean)

cor(mm.all.dinucleotide.di, mm.average.di)
summary(lm(mm.all.dinucleotide.di ~ mm.average.di))

## D. rerio
# Get promoters DI
dr.DI.promoters <- read.table("data/drCageDispersion.dat.gz", row.names=1, )
dr.DI.promoters[dr.DI.promoters == -1] <- NA
dr.DI.promoters.median <- apply(dr.DI.promoters, 1, mean, na.rm=T)
plot(density(dr.DI.promoters.median, na.rm=T))

# Get average raw signals for promoters:
dr.average.yy.proxprom <- cbind(rowMeans(dr.promoters.raw.yy[,range.nfr], na.rm=T),
   rowMeans(dr.promoters.raw.yy[,range.1n], na.rm=T))
dr.average.rr.proxprom <- cbind(rowMeans(dr.promoters.raw.rr[,range.nfr], na.rm=T),
   rowMeans(dr.promoters.raw.rr[,range.1n], na.rm=T))
dr.average.ww.proxprom <- cbind(rowMeans(dr.promoters.raw.ww[,range.nfr], na.rm=T),
   rowMeans(dr.promoters.raw.ww[,range.1n], na.rm=T))
dr.average.ss.proxprom <- cbind(rowMeans(dr.promoters.raw.ss[,range.nfr], na.rm=T),
   rowMeans(dr.promoters.raw.ss[,range.1n], na.rm=T))

dr.average.ww.di.tata <- mean(dr.average.ww.proxprom[dr.tata.promoters,2], na.rm=T)
dr.average.rr.di.tata <- mean(dr.average.rr.proxprom[dr.tata.promoters,2], na.rm=T)
```

```
dr.average.yy.di.tata <- mean(dr.average.yy.proxprom[dr.tata.promoters,2], na.rm=T)
dr.average.ss.di.tata <- mean(dr.average.ss.proxprom[dr.tata.promoters,2], na.rm=T)
#dr.average.dinucleotite.tata <- mean(c(dr.average.ww.di.tata, dr.average.rr.di.tata,
   dr.average.yy.di.tata, dr.average.ss.di.tata))
dr.average.dinucleotite.tata <- dr.average.ww.di.tata

dr.average.di.tata <- mean(dr.DI.promoters.median[dr.tata.promoters], na.rm=T)

dr.tataless <- rownames(dr.promoterAnnotation[dr.promoterAnnotation[,1] == 0,])
dr.ordered.DI <- names(sort(dr.DI.promoters.median[dr.tataless]))
g.intervals <- cbind(seq(1, length(dr.ordered.DI), 1000), c(seq(1000, length(dr.ordered.DI),
   1000), length(dr.ordered.DI)))

dr.average.ww.di <-
   c(mean(dr.average.ww.proxprom[dr.ordered.DI[g.intervals[1,1]:g.intervals[1,2]],2]),
   mean(dr.average.ww.proxprom[dr.ordered.DI[g.intervals[2,1]:g.intervals[2,2]],2]),
   mean(dr.average.ww.proxprom[dr.ordered.DI[g.intervals[3,1]:g.intervals[3,2]],2]),
   mean(dr.average.ww.proxprom[dr.ordered.DI[g.intervals[4,1]:g.intervals[4,2]],2]),
   mean(dr.average.ww.proxprom[dr.ordered.DI[g.intervals[5,1]:g.intervals[5,2]],2]),
   mean(dr.average.ww.proxprom[dr.ordered.DI[g.intervals[6,1]:g.intervals[6,2]],2]),
   mean(dr.average.ww.proxprom[dr.ordered.DI[g.intervals[7,1]:g.intervals[7,2]],2]),
   mean(dr.average.ww.proxprom[dr.ordered.DI[g.intervals[8,1]:g.intervals[8,2]],2]),
   mean(dr.average.ww.proxprom[dr.ordered.DI[g.intervals[9,1]:g.intervals[9,2]],2]))

dr.average.ss.di <-
   c(mean(dr.average.ss.proxprom[dr.ordered.DI[g.intervals[1,1]:g.intervals[1,2]],2]),
   mean(dr.average.ss.proxprom[dr.ordered.DI[g.intervals[2,1]:g.intervals[2,2]],2]),
   mean(dr.average.ss.proxprom[dr.ordered.DI[g.intervals[3,1]:g.intervals[3,2]],2]),
   mean(dr.average.ss.proxprom[dr.ordered.DI[g.intervals[4,1]:g.intervals[4,2]],2]),
   mean(dr.average.ss.proxprom[dr.ordered.DI[g.intervals[5,1]:g.intervals[5,2]],2]),
   mean(dr.average.ss.proxprom[dr.ordered.DI[g.intervals[6,1]:g.intervals[6,2]],2]),
   mean(dr.average.ss.proxprom[dr.ordered.DI[g.intervals[7,1]:g.intervals[7,2]],2]),
   mean(dr.average.ss.proxprom[dr.ordered.DI[g.intervals[8,1]:g.intervals[8,2]],2]),
   mean(dr.average.ss.proxprom[dr.ordered.DI[g.intervals[9,1]:g.intervals[9,2]],2]))

dr.average.yy.di <-
   c(mean(dr.average.yy.proxprom[dr.ordered.DI[g.intervals[1,1]:g.intervals[1,2]],2]),
   mean(dr.average.yy.proxprom[dr.ordered.DI[g.intervals[2,1]:g.intervals[2,2]],2]),
   mean(dr.average.yy.proxprom[dr.ordered.DI[g.intervals[3,1]:g.intervals[3,2]],2]),
   mean(dr.average.yy.proxprom[dr.ordered.DI[g.intervals[4,1]:g.intervals[4,2]],2]),
   mean(dr.average.yy.proxprom[dr.ordered.DI[g.intervals[5,1]:g.intervals[5,2]],2]),
   mean(dr.average.yy.proxprom[dr.ordered.DI[g.intervals[6,1]:g.intervals[6,2]],2]),
   mean(dr.average.yy.proxprom[dr.ordered.DI[g.intervals[7,1]:g.intervals[7,2]],2]),
   mean(dr.average.yy.proxprom[dr.ordered.DI[g.intervals[8,1]:g.intervals[8,2]],2]),
   mean(dr.average.yy.proxprom[dr.ordered.DI[g.intervals[9,1]:g.intervals[9,2]],2]))

dr.average.rr.di <-
   c(mean(dr.average.rr.proxprom[dr.ordered.DI[g.intervals[1,1]:g.intervals[1,2]],2]),
   mean(dr.average.rr.proxprom[dr.ordered.DI[g.intervals[2,1]:g.intervals[2,2]],2]),
   mean(dr.average.rr.proxprom[dr.ordered.DI[g.intervals[3,1]:g.intervals[3,2]],2]),
   mean(dr.average.rr.proxprom[dr.ordered.DI[g.intervals[4,1]:g.intervals[4,2]],2]),
   mean(dr.average.rr.proxprom[dr.ordered.DI[g.intervals[5,1]:g.intervals[5,2]],2]),
   mean(dr.average.rr.proxprom[dr.ordered.DI[g.intervals[6,1]:g.intervals[6,2]],2]),
   mean(dr.average.rr.proxprom[dr.ordered.DI[g.intervals[7,1]:g.intervals[7,2]],2]),
   mean(dr.average.rr.proxprom[dr.ordered.DI[g.intervals[8,1]:g.intervals[8,2]],2]),
```

```
    mean(dr.average.rr.proxprom[dr.ordered.DI[g.intervals[9,1]:g.intervals[9,2]],2]))

dr.average.di <-
    c(mean(dr.DI.promoters.median[dr.ordered.DI[g.intervals[1,1]:g.intervals[1,2]]]),
    mean(dr.DI.promoters.median[dr.ordered.DI[g.intervals[2,1]:g.intervals[2,2]]]),
    mean(dr.DI.promoters.median[dr.ordered.DI[g.intervals[3,1]:g.intervals[3,2]]]),
    mean(dr.DI.promoters.median[dr.ordered.DI[g.intervals[4,1]:g.intervals[4,2]]]),
    mean(dr.DI.promoters.median[dr.ordered.DI[g.intervals[5,1]:g.intervals[5,2]]]),
    mean(dr.DI.promoters.median[dr.ordered.DI[g.intervals[6,1]:g.intervals[6,2]]]),
    mean(dr.DI.promoters.median[dr.ordered.DI[g.intervals[7,1]:g.intervals[7,2]]]),
    mean(dr.DI.promoters.median[dr.ordered.DI[g.intervals[8,1]:g.intervals[8,2]]]),
    mean(dr.DI.promoters.median[dr.ordered.DI[g.intervals[9,1]:g.intervals[9,2]]]))

dr.average.dinucleotide.di <- cbind(dr.average.ww.di, dr.average.yy.di, dr.average.ss.di,
    dr.average.rr.di)
#dr.all.dinucleotide.di <- apply(dr.average.dinucleotide.di,1,mean)
dr.all.dinucleotide.di <- dr.average.ww.di

cor(dr.all.dinucleotide.di, dr.average.di)
summary(lm(dr.all.dinucleotide.di[-1] ~ dr.average.di[-1]))

## D. melanogaster
# Get promoters DI
dm.DI.promoters <- read.table("data/dmCageDispersion.dat.gz", row.names=1)
dm.DI.promoters[dm.DI.promoters == -1] <- NA
dm.DI.promoters[dm.DI.promoters == 0] <- NA
dm.DI.promoters.median <- apply(dm.DI.promoters, 1, mean, na.rm=T)
plot(density(dm.DI.promoters.median, na.rm=T))

# Get average raw signals for promoters:
dm.average.yy.proxprom <- cbind(rowMeans(dm.promoters.raw.yy[,range.nfr], na.rm=T),
    rowMeans(dm.promoters.raw.yy[,range.1n], na.rm=T))
dm.average.rr.proxprom <- cbind(rowMeans(dm.promoters.raw.rr[,range.nfr], na.rm=T),
    rowMeans(dm.promoters.raw.rr[,range.1n], na.rm=T))
dm.average.ww.proxprom <- cbind(rowMeans(dm.promoters.raw.ww[,range.nfr], na.rm=T),
    rowMeans(dm.promoters.raw.ww[,range.1n], na.rm=T))
dm.average.ss.proxprom <- cbind(rowMeans(dm.promoters.raw.ss[,range.nfr], na.rm=T),
    rowMeans(dm.promoters.raw.ss[,range.1n], na.rm=T))

dm.average.ww.di.tata <- mean(dm.average.ww.proxprom[dm.tata.promoters,2], na.rm=T)
dm.average.rr.di.tata <- mean(dm.average.rr.proxprom[dm.tata.promoters,2], na.rm=T)
dm.average.yy.di.tata <- mean(dm.average.yy.proxprom[dm.tata.promoters,2], na.rm=T)
dm.average.ss.di.tata <- mean(dm.average.ss.proxprom[dm.tata.promoters,2], na.rm=T)
dm.average.dinucleotite.tata <- mean(c(dm.average.ww.di.tata, dm.average.rr.di.tata,
    dm.average.yy.di.tata, dm.average.ss.di.tata))
dm.average.di.tata <- mean(dm.DI.promoters.median[dm.tata.promoters], na.rm=T)

# InrDpe
dm.inrdpe.promoters <- rownames(dm.promoterAnnotation)[which(dm.promoterAnnotation[,3] == 1)]
dm.average.ww.di.inrdpe <- mean(dm.average.ww.proxprom[dm.inrdpe.promoters,2], na.rm=T)
```

```
dm.average.rr.di.inrdpe <- mean(dm.average.rr.proxprom[dm.inrdpe.promoters,2], na.rm=T)
dm.average.yy.di.inrdpe <- mean(dm.average.yy.proxprom[dm.inrdpe.promoters,2], na.rm=T)
dm.average.ss.di.inrdpe <- mean(dm.average.ss.proxprom[dm.inrdpe.promoters,2], na.rm=T)
dm.average.dinucleotite.inrdpe <- mean(c(dm.average.ww.di.inrdpe, dm.average.rr.di.inrdpe,
   dm.average.yy.di.inrdpe, dm.average.ss.di.inrdpe))
dm.average.di.inrdpe <- mean(dm.DI.promoters.median[dm.inrdpe.promoters], na.rm=T)



dm.tataless <- rownames(dm.promoterAnnotation[dm.promoterAnnotation[,1] == 0,])
dm.dpeless <- dm.tataless[dm.promoterAnnotation[dm.tataless,3] == 0]


dm.ordered.DI <- names(sort(dm.DI.promoters.median[dm.dpeless]))
g.intervals <- cbind(seq(1, length(dm.ordered.DI), 2000), c(seq(2000, length(dm.ordered.DI),
   2000), length(dm.ordered.DI)))


dm.average.ww.di <-
   c(mean(dm.average.ww.proxprom[dm.ordered.DI[g.intervals[1,1]:g.intervals[1,2]],2]),
   mean(dm.average.ww.proxprom[dm.ordered.DI[g.intervals[2,1]:g.intervals[2,2]],2]),
   mean(dm.average.ww.proxprom[dm.ordered.DI[g.intervals[3,1]:g.intervals[3,2]],2]),
   mean(dm.average.ww.proxprom[dm.ordered.DI[g.intervals[4,1]:g.intervals[4,2]],2]))


dm.average.ss.di <-
   c(mean(dm.average.ss.proxprom[dm.ordered.DI[g.intervals[1,1]:g.intervals[1,2]],2]),
   mean(dm.average.ss.proxprom[dm.ordered.DI[g.intervals[2,1]:g.intervals[2,2]],2]),
   mean(dm.average.ss.proxprom[dm.ordered.DI[g.intervals[3,1]:g.intervals[3,2]],2]),
   mean(dm.average.ss.proxprom[dm.ordered.DI[g.intervals[4,1]:g.intervals[4,2]],2]))


dm.average.yy.di <-
   c(mean(dm.average.yy.proxprom[dm.ordered.DI[g.intervals[1,1]:g.intervals[1,2]],2]),
   mean(dm.average.yy.proxprom[dm.ordered.DI[g.intervals[2,1]:g.intervals[2,2]],2]),
   mean(dm.average.yy.proxprom[dm.ordered.DI[g.intervals[3,1]:g.intervals[3,2]],2]),
   mean(dm.average.yy.proxprom[dm.ordered.DI[g.intervals[4,1]:g.intervals[4,2]],2]))


dm.average.rr.di <-
   c(mean(dm.average.rr.proxprom[dm.ordered.DI[g.intervals[1,1]:g.intervals[1,2]],2]),
   mean(dm.average.rr.proxprom[dm.ordered.DI[g.intervals[2,1]:g.intervals[2,2]],2]),
   mean(dm.average.rr.proxprom[dm.ordered.DI[g.intervals[3,1]:g.intervals[3,2]],2]),
   mean(dm.average.rr.proxprom[dm.ordered.DI[g.intervals[4,1]:g.intervals[4,2]],2]))


dm.average.di <-
   c(mean(dm.DI.promoters.median[dm.ordered.DI[g.intervals[1,1]:g.intervals[1,2]]]),
   mean(dm.DI.promoters.median[dm.ordered.DI[g.intervals[2,1]:g.intervals[2,2]]]),
   mean(dm.DI.promoters.median[dm.ordered.DI[g.intervals[3,1]:g.intervals[3,2]]]),
   mean(dm.DI.promoters.median[dm.ordered.DI[g.intervals[4,1]:g.intervals[4,2]]]))



dm.average.dinucleotide.di <- cbind(dm.average.ww.di, dm.average.yy.di, dm.average.rr.di,
   dm.average.ss.di)
dm.all.dinucleotide.di <- apply(dm.average.dinucleotide.di,1,mean)


cor(all.dinucleotide.di, dm.average.di)
summary(lm(all.dinucleotide.di[-1] ~ dm.average.di[-1]))
```

```
## C. elegans
# Get promoters DI
ce.DI.promoters <- read.table("data/ceCageDispersion.dat.gz", row.names=1, )
ce.DI.promoters[ce.DI.promoters == -1] <- NA
#ce.DI.promoters[ce.DI.promoters == 0] <- NA
ce.DI.promoters.median <- apply(ce.DI.promoters, 1, mean, na.rm=T)
plot(density(ce.DI.promoters.median, na.rm=T))

# Get average raw signals for promoters:
ce.average.yy.proxprom <- cbind(rowMeans(ce.promoters.raw.yy[,range.nfr], na.rm=T),
   rowMeans(ce.promoters.raw.yy[,range.1n], na.rm=T))
ce.average.rr.proxprom <- cbind(rowMeans(ce.promoters.raw.rr[,range.nfr], na.rm=T),
   rowMeans(ce.promoters.raw.rr[,range.1n], na.rm=T))
ce.average.ww.proxprom <- cbind(rowMeans(ce.promoters.raw.ww[,range.nfr], na.rm=T),
   rowMeans(ce.promoters.raw.ww[,range.1n], na.rm=T))
ce.average.ss.proxprom <- cbind(rowMeans(ce.promoters.raw.ss[,range.nfr], na.rm=T),
   rowMeans(ce.promoters.raw.ss[,range.1n], na.rm=T))

ce.average.ww.di.tata <- mean(ce.average.ww.proxprom[ce.tata.promoters,2], na.rm=T)
ce.average.rr.di.tata <- mean(ce.average.rr.proxprom[ce.tata.promoters,2], na.rm=T)
ce.average.yy.di.tata <- mean(ce.average.yy.proxprom[ce.tata.promoters,2], na.rm=T)
ce.average.ss.di.tata <- mean(ce.average.ss.proxprom[ce.tata.promoters,2], na.rm=T)
ce.average.dinucleotite.tata <- mean(c(ce.average.ww.di.tata, ce.average.rr.di.tata,
   ce.average.yy.di.tata, ce.average.ss.di.tata))

ce.average.di.tata <- mean(ce.DI.promoters.median[ce.tata.promoters], na.rm=T)

ce.tataless <- rownames(ce.promoterAnnotation[ce.promoterAnnotation[,1] == 0,])
ce.ordered.DI <- names(sort(ce.DI.promoters.median[ce.tataless]))
g.intervals <- cbind(seq(1, length(ce.ordered.DI), 1000), c(seq(1000, length(ce.ordered.DI),
   1000), length(ce.ordered.DI)))

ce.average.ww.di <-
   c(mean(ce.average.ww.proxprom[ce.ordered.DI[g.intervals[1,1]:g.intervals[1,2]],2]),
   mean(ce.average.ww.proxprom[ce.ordered.DI[g.intervals[2,1]:g.intervals[2,2]],2]),
   mean(ce.average.ww.proxprom[ce.ordered.DI[g.intervals[3,1]:g.intervals[3,2]],2]),
   mean(ce.average.ww.proxprom[ce.ordered.DI[g.intervals[4,1]:g.intervals[4,2]],2]),
   mean(ce.average.ww.proxprom[ce.ordered.DI[g.intervals[5,1]:g.intervals[5,2]],2]),
   mean(ce.average.ww.proxprom[ce.ordered.DI[g.intervals[6,1]:g.intervals[6,2]],2]),
   mean(ce.average.ww.proxprom[ce.ordered.DI[g.intervals[7,1]:g.intervals[7,2]],2]))

ce.average.ss.di <-
   c(mean(ce.average.ss.proxprom[ce.ordered.DI[g.intervals[1,1]:g.intervals[1,2]],2]),
   mean(ce.average.ss.proxprom[ce.ordered.DI[g.intervals[2,1]:g.intervals[2,2]],2]),
   mean(ce.average.ss.proxprom[ce.ordered.DI[g.intervals[3,1]:g.intervals[3,2]],2]),
   mean(ce.average.ss.proxprom[ce.ordered.DI[g.intervals[4,1]:g.intervals[4,2]],2]),
   mean(ce.average.ss.proxprom[ce.ordered.DI[g.intervals[5,1]:g.intervals[5,2]],2]),
   mean(ce.average.ss.proxprom[ce.ordered.DI[g.intervals[6,1]:g.intervals[6,2]],2]),
   mean(ce.average.ss.proxprom[ce.ordered.DI[g.intervals[7,1]:g.intervals[7,2]],2]))
```

```
ce.average.yy.di <-
   c(mean(ce.average.yy.proxprom[ce.ordered.DI[g.intervals[1,1]:g.intervals[1,2]],2]),
   mean(ce.average.yy.proxprom[ce.ordered.DI[g.intervals[2,1]:g.intervals[2,2]],2]),
   mean(ce.average.yy.proxprom[ce.ordered.DI[g.intervals[3,1]:g.intervals[3,2]],2]),
   mean(ce.average.yy.proxprom[ce.ordered.DI[g.intervals[4,1]:g.intervals[4,2]],2]),
   mean(ce.average.yy.proxprom[ce.ordered.DI[g.intervals[5,1]:g.intervals[5,2]],2]),
   mean(ce.average.yy.proxprom[ce.ordered.DI[g.intervals[6,1]:g.intervals[6,2]],2]),
   mean(ce.average.yy.proxprom[ce.ordered.DI[g.intervals[7,1]:g.intervals[7,2]],2]))


ce.average.rr.di <-
   c(mean(ce.average.rr.proxprom[ce.ordered.DI[g.intervals[1,1]:g.intervals[1,2]],2]),
   mean(ce.average.rr.proxprom[ce.ordered.DI[g.intervals[2,1]:g.intervals[2,2]],2]),
   mean(ce.average.rr.proxprom[ce.ordered.DI[g.intervals[3,1]:g.intervals[3,2]],2]),
   mean(ce.average.rr.proxprom[ce.ordered.DI[g.intervals[4,1]:g.intervals[4,2]],2]),
   mean(ce.average.rr.proxprom[ce.ordered.DI[g.intervals[5,1]:g.intervals[5,2]],2]),
   mean(ce.average.rr.proxprom[ce.ordered.DI[g.intervals[6,1]:g.intervals[6,2]],2]),
   mean(ce.average.rr.proxprom[ce.ordered.DI[g.intervals[7,1]:g.intervals[7,2]],2]))


ce.average.di <-
   c(mean(ce.DI.promoters.median[ce.ordered.DI[g.intervals[1,1]:g.intervals[1,2]]]),
   mean(ce.DI.promoters.median[ce.ordered.DI[g.intervals[2,1]:g.intervals[2,2]]]),
   mean(ce.DI.promoters.median[ce.ordered.DI[g.intervals[3,1]:g.intervals[3,2]]]),
   mean(ce.DI.promoters.median[ce.ordered.DI[g.intervals[4,1]:g.intervals[4,2]]]),
   mean(ce.DI.promoters.median[ce.ordered.DI[g.intervals[5,1]:g.intervals[5,2]]]),
   mean(ce.DI.promoters.median[ce.ordered.DI[g.intervals[6,1]:g.intervals[6,2]]]),
   mean(ce.DI.promoters.median[ce.ordered.DI[g.intervals[7,1]:g.intervals[7,2]]]))


ce.average.dinucleotide.di <- cbind(ce.average.ww.di, ce.average.yy.di, ce.average.ss.di,
   ce.average.rr.di)
ce.all.dinucleotide.di <- apply(ce.average.dinucleotide.di,1,mean)


cor(all.dinucleotide.di, ce.average.di)
summary(lm(all.dinucleotide.di[-1] ~ ce.average.di[-1]))
```

3.11. Correlation analysis between DI and average promoter expression level

The next chunk of code is very similar to the one present in the previous section. The only difference is that the correlation this time is done between the DI and the average expression level of promoters. The expression is evaluated as the sum of CAGE tags in a 100 bp region around the TSS:

```
hs.expression <- read.table("data/cage_cell_longPolyA_expression.dat",
   header=T, row.names=1)[rownames(hs.promoters.raw.yy),]
hs.average.expression <- rowMeans(hs.expression)
hs.sd.expression <- apply(hs.expression, 1, sd)
hs.cv.expression <- hs.sd.expression/hs.average.expression


average.exprs.tata <- mean(hs.average.expression[hs.tata.promoters],
   na.rm=T)
ordered.EXPRS <- names(sort(hs.average.expression[hs.promoterAnnotation[,1]
   == 0]))


average.ww.exprs <- c(mean(average.ww.proxprom[ordered.EXPRS[1:2000],2]),
```

```
  mean(average.ww.proxprom[ordered.EXPRS[2001:4000],2]),
  mean(average.ww.proxprom[ordered.EXPRS[4001:6000],2]),
  mean(average.ww.proxprom[ordered.EXPRS[6001:8000],2]),
  mean(average.ww.proxprom[ordered.EXPRS[8001:10000],2]),
  mean(average.ww.proxprom[ordered.EXPRS[10001:12000],2]),
  mean(average.ww.proxprom[ordered.EXPRS[12001:14000],2]),
  mean(average.ww.proxprom[ordered.EXPRS[14001:16000],2]),
  mean(average.ww.proxprom[ordered.EXPRS[16001:18000],2]),
  mean(average.ww.proxprom[ordered.EXPRS[18001:20137],2]))

average.ss.exprs <- c(mean(average.ss.proxprom[ordered.EXPRS[1:2000],2]),
  mean(average.ss.proxprom[ordered.EXPRS[2001:4000],2]),
  mean(average.ss.proxprom[ordered.EXPRS[4001:6000],2]),
  mean(average.ss.proxprom[ordered.EXPRS[6001:8000],2]),
  mean(average.ss.proxprom[ordered.EXPRS[8001:10000],2]),
  mean(average.ss.proxprom[ordered.EXPRS[10001:12000],2]),
  mean(average.ss.proxprom[ordered.EXPRS[12001:14000],2]),
  mean(average.ss.proxprom[ordered.EXPRS[14001:16000],2]),
  mean(average.ss.proxprom[ordered.EXPRS[16001:18000],2]),
  mean(average.ss.proxprom[ordered.EXPRS[18001:20137],2]))

average.yy.exprs <- c(mean(average.yy.proxprom[ordered.EXPRS[1:2000],2]),
  mean(average.yy.proxprom[ordered.EXPRS[2001:4000],2]),
  mean(average.yy.proxprom[ordered.EXPRS[4001:6000],2]),
  mean(average.yy.proxprom[ordered.EXPRS[6001:8000],2]),
  mean(average.yy.proxprom[ordered.EXPRS[8001:10000],2]),
  mean(average.yy.proxprom[ordered.EXPRS[10001:12000],2]),
  mean(average.yy.proxprom[ordered.EXPRS[12001:14000],2]),
  mean(average.yy.proxprom[ordered.EXPRS[14001:16000],2]),
  mean(average.yy.proxprom[ordered.EXPRS[16001:18000],2]),
  mean(average.yy.proxprom[ordered.EXPRS[18001:20137],2]))

average.rr.exprs <- c(mean(average.rr.proxprom[ordered.EXPRS[1:2000],2]),
  mean(average.rr.proxprom[ordered.EXPRS[2001:4000],2]),
  mean(average.rr.proxprom[ordered.EXPRS[4001:6000],2]),
  mean(average.rr.proxprom[ordered.EXPRS[6001:8000],2]),
  mean(average.rr.proxprom[ordered.EXPRS[8001:10000],2]),
  mean(average.rr.proxprom[ordered.EXPRS[10001:12000],2]),
  mean(average.rr.proxprom[ordered.EXPRS[12001:14000],2]),
  mean(average.rr.proxprom[ordered.EXPRS[14001:16000],2]),
  mean(average.rr.proxprom[ordered.EXPRS[16001:18000],2]),
  mean(average.rr.proxprom[ordered.EXPRS[18001:20137],2]))

average.exprs <- c(median(hs.average.expression[ordered.EXPRS[1:2000]]),
  median(hs.average.expression[ordered.EXPRS[2001:4000]]),
  median(hs.average.expression[ordered.EXPRS[4001:6000]]),
  median(hs.average.expression[ordered.EXPRS[6001:8000]]),
  median(hs.average.expression[ordered.EXPRS[8001:10000]]),
  median(hs.average.expression[ordered.EXPRS[10001:12000]]),
  median(hs.average.expression[ordered.EXPRS[12001:14000]]),
  median(hs.average.expression[ordered.EXPRS[14001:16000]]),
```

```
   median(hs.average.expression[ordered.EXPRS[16001:18000]]),
   median(hs.average.expression[ordered.EXPRS[18001:20137]]))

average.dinucleotide.exprs <- cbind(average.ww.exprs, average.yy.exprs,
   average.ss.exprs, average.rr.exprs)
all.dinucleotide.exprs <- apply(average.dinucleotide.exprs,1,mean)

cor(all.dinucleotide.exprs, average.exprs)
```

3.12. CAGE micro-peak extraction around promoters

This sub-section describes the analysis of CAGE data distribution around promoters. Raw data is formatted similarly as nucleosome data presented in a previous sub-section: a matrix in which each row represents a promoter and each column a position around the TSS (from base -103 to base 104, step 1 base). In order to reduce noise in the data, micro-peaks of 4 bases window (`win` parameter) are extracted from CAGE data with arbitrary chosen threshold (`th` parameter) that is roughly proportional to the total number of tags for each organism. Micro-peaks are extracted using the R function `find.peaks` that is presented in the Functions definition section at the end of this document. The function returns the number of tags that are present in a region of window `win` and centered on the position with the higher tag count. Moreover, in order to reduce the influence of few hyper-expressed promoters, we applied for each peak a count cut-off (`co` parameter) of 10. The following is the analysis of human data:

```
hs.cage <- as.matrix(read.table("data/Hs_EPDnew_002a_hg19_CAGEtags.dat",
       row.names=1, header=F))
mm.cage <- as.matrix(read.table("data/Mm_EPDnew_002_mm9_CAGEtags.dat",
       row.names=1, header=F))
dr.cage <- as.matrix(read.table("data/Dr_EPDnew_001_danRer7_CAGEtags.dat",
       row.names=1, header=F))
dm.cage <- as.matrix(read.table("data/Dm_EPDnew_002_dm3_CAGEtags.dat",
       row.names=1, header=F))
ce.cage <- as.matrix(read.table("data/Ce_EPDnew_001_ce6_CAGEtags.dat",
       row.names=1, header=F))
colnames(hs.cage) <- -103:104
win <- 4
co <- 1
L <- dim(hs.cage)[2]
X <- (1:L)-(L/2)

th <- 100
hs.cage.micropeaks <- t(apply(hs.cage, 1, find.peaks, win, th))
hs.cage.micropeaks[hs.cage.micropeaks > co] <- co
plot(X,predict(loess(colMeans(hs.cage.micropeaks)~X, span=0.05)),t="l")

th <- 100
mm.cage.micropeaks <- t(apply(mm.cage, 1, find.peaks, win, th))
mm.cage.micropeaks[mm.cage.micropeaks > co] <- co
plot(X,predict(loess(colMeans(mm.cage.micropeaks)~X, span=0.05)),t="l")

th <- 100
```

```
dr.cage.micropeaks <- t(apply(dr.cage, 1, find.peaks, win, th))
dr.cage.micropeaks[dr.cage.micropeaks > co] <- co
plot(X,predict(loess(colMeans(dr.cage.micropeaks)~X, span=0.05)),t="l")

th <- 100
dm.cage.micropeaks <- t(apply(dm.cage, 1, find.peaks, win, th))
dm.cage.micropeaks[dm.cage.micropeaks > co] <- co
plot(X,predict(loess(colMeans(dm.cage.micropeaks)~X, span=0.05)),t="l")

th <- 100
ce.cage.micropeaks <- t(apply(ce.cage, 1, find.peaks, win, th))
ce.cage.micropeaks[ce.cage.micropeaks > co] <- co
plot(X,predict(loess(colMeans(ce.cage.micropeaks)~X, span=0.05)),t="l")
```

The next part of the code describe the steps used to extract promoters with a strong Pol-II periodic signal:

```
# compute covariance vector with cosine function
range <- c(70:99,109:138)
N <- dim(hs.cage.micropeaks)[1]
c <- rep(0,N)
for(i in 1:N) {
  c[i] <- cov(hs.cage.micropeaks[i,range],cos((range-54)/10.0*pi*2))
}

# define positive and negative subsets
d <- sort(c)
s <- rep(0,N)
for(i in 1:N) {
  s[i] <- sum(d[1:i])
}
pos <- order(c)[N:(N-length(which(s > 0))+1)]
neg <- order(c)[(N-length(which(s > 0))):1]
print(length(pos)); print(length(neg))
pos.names <- row.names(hs.cage.micropeaks)[pos]
neg.names <- row.names(hs.cage.micropeaks)[neg]

hs.pos.yy.freq <- find.freq(hs.promoters[pos.names,], type="YY")
hs.pos.rr.freq <- find.freq(hs.promoters[pos.names,], type="RR")
hs.pos.ww.freq <- find.freq(hs.promoters[pos.names,], type="WW")
hs.pos.ss.freq <- find.freq(hs.promoters[pos.names,], type="SS")

hs.neg.yy.freq <- find.freq(hs.promoters[neg.names,], type="YY")
hs.neg.rr.freq <- find.freq(hs.promoters[neg.names,], type="RR")
hs.neg.ww.freq <- find.freq(hs.promoters[neg.names,], type="WW")
hs.neg.ss.freq <- find.freq(hs.promoters[neg.names,], type="SS")

region <- as.character(seq(50, 200, 1))
hs.spec.pos <- spec.pgram(hs.pos.yy.freq[region], span=2, log="yes")
plot(1/hs.spec.pos$freq,hs.spec.pos$spec, type="l", xlim=c(0,25),
```

```
      frame.plot="F", xlab="Period (nt)", ylab="Power spectrum", lwd=6,
      main="Spectrum decomposition (H. sapiens)", cex.axis=1.2,
      cex.main=1.5, cex.lab=1.2)

microPeaks.positive <- list(pos.promoters=pos.names,
      neg.promoters=neg.names,
      hs.pos.micropeaks=predict(loess(colMeans(hs.cage.micropeaks[pos.names
      ,])~X, span=0.05)),
      hs.neg.micropeaks=predict(loess(colMeans(hs.cage.micropeaks[neg.names
      ,])~X, span=0.05)), hs.pos.yy.freq=hs.pos.yy.freq,
      hs.neg.yy.freq=hs.neg.yy.freq)
save(microPeaks.positive, file="data/microPeaks.positive.RData")
```

3.13. SNPs and Indels distribution around *H. sapiens* promoters

This sub-section is focused on studing the SNPs and Indels distribution aroung promoters of the GM12878 cell line (a lymphoblastoid cell). As a reference we have developed the “Most Likely Genome” (ML genome) that uses the locus with the higher frequency for each SNP and Indel map in the cell line. This step is necessary since we do not have other cell lines for which we have both the genome and CAGE data (important for study the Pol-II dinamics and how it is affected by the presence of the natural variants). As CAGE reference we used other blood related cell lines from the ENCODE and FANTOM projects and assigned them to the ML genome. The following are the R commands to make the analysis. The first step is to read in the sequences of the new genomes:

```
hs.raw.sequences <- read.fasta("data/Hs_EPDnew_002a_MostProbableGenome.fa")
hs.names <- names(hs.raw.sequences)
hs.sequences <- matrix(data=unlist(hs.raw.sequences),
     nrow=length(hs.raw.sequences), ncol=length(hs.raw.sequences[[1]]),
     byrow=TRUE)
rownames(hs.sequences) <- hs.names
hs.promoters <- seq.trans(hs.sequences)
colnames(hs.promoters) <- -1074:1075

gm12878.raw.sequences <- read.fasta("data/Hs_EPDnew_002a_GM12878.fa")
gm12878.names <- names(gm12878.raw.sequences)
gm12878.sequences <- matrix(data=unlist(gm12878.raw.sequences),
     nrow=length(gm12878.raw.sequences),
     ncol=length(gm12878.raw.sequences[[1]]), byrow=TRUE)
rownames(gm12878.sequences) <- gm12878.names
gm12878.promoters <- seq.trans(gm12878.sequences)
colnames(gm12878.promoters) <- -1074:1075

gm12878.indels <- read.table("data/GM12878PromotersRealIndels.txt",
     sep="\t", as.is=T)
gm12878.snps <- read.table("data/GM12878PromotersRealSnps.txt", sep="\t",
     as.is=T)

# Get the promoters that have indels and extract them from the GS12878
# genome and from the reference:
```

```
gm12878.promoters.indels.names <- gm12878.indels[unique(which(
     (gm12878.indels[,2] >= -1000) & (gm12878.indels[,2] < 1000) )),1]


gm12878.promotersWithIndels <-
     gm12878.promoters[gm12878.promoters.indels.names,]
hs.promotersWithIndels <- hs.promoters[gm12878.promoters.indels.names,]


intervals <- seq(-1000, 1000, 10)
```

Then calculate the WW frequency in the GM12878 promoters and in the ML genome (restrict this analysis only to promoters that have natural variants). This is performed first on indels and then on SNPs.

```
########################################################################
# WW for the GM12878 genome (Indels):
gm12878.promotersWithIndels.raw.ww <- matrix(0, ncol=length(intervals),
     nrow=dim(gm12878.promotersWithIndels)[1])
colnames(gm12878.promotersWithIndels.raw.ww) <- intervals
rownames(gm12878.promotersWithIndels.raw.ww) <-
     rownames(gm12878.promotersWithIndels)
for (I in intervals){
  region <- as.character(seq(I-74, I+75, 1))
  gm12878.region <- gm12878.promotersWithIndels[,region]
  gm12878.region.raw <- apply(gm12878.region, 1, raw.spectra, "WW", 10)
  cat(I, "\t", mean(gm12878.region.raw, na.rm=T), "\n")
  gm12878.promotersWithIndels.raw.ww[,which(intervals == I)] <-
     gm12878.region.raw
  plot(intervals, colMeans(gm12878.promotersWithIndels.raw.ww, na.rm=T),
     type="b", pch=19,
     ylim=range(colMeans(gm12878.promotersWithIndels.raw.ww,
     na.rm=T)[colMeans(gm12878.promotersWithIndels.raw.ww, na.rm=T)>0]))
}
# And for the reference:
hs.promotersWithIndels.raw.ww <- matrix(0, ncol=length(intervals),
     nrow=dim(hs.promotersWithIndels)[1])
colnames(hs.promotersWithIndels.raw.ww) <- intervals
rownames(hs.promotersWithIndels.raw.ww) <- rownames(hs.promotersWithIndels)
for (I in intervals){
  region <- as.character(seq(I-74, I+75, 1))
  hs.region <- hs.promotersWithIndels[,region]
  hs.region.raw <- apply(hs.region, 1, raw.spectra, "WW", 10)
  cat(I, "\t", mean(hs.region.raw, na.rm=T), "\n")
  hs.promotersWithIndels.raw.ww[,which(intervals == I)] <- hs.region.raw
  plot(intervals, colMeans(hs.promotersWithIndels.raw.ww, na.rm=T),
     type="b", pch=19, ylim=range(colMeans(hs.promotersWithIndels.raw.ww,
     na.rm=T)[colMeans(hs.promotersWithIndels.raw.ww, na.rm=T)>0]))
```

```
}
# plot them:
plot(intervals, colMeans(gm12878.promotersWithIndels.raw.ww, na.rm=T),
     type="b", pch=19)
points(intervals, colMeans(hs.promotersWithIndels.raw.ww, na.rm=T),
     type="b", pch=19, col="red")

######################################################################
### Do the same for SNPs:
######################################################################
gm12878.promoters.snps.names <- gm12878.snps[unique(which(
     (gm12878.snps[,2] >= -1000) & (gm12878.snps[,2] < 1000) )),1]
gm12878.promotersWithSnps <-
     gm12878.promoters[gm12878.promoters.snps.names,]
hs.promotersWithSnps <- hs.promoters[gm12878.promoters.snps.names,]

######################################################################
# WW for the GM12878 genome:
gm12878.promotersWithSnps.raw.ww <- matrix(0, ncol=length(intervals),
     nrow=dim(gm12878.promotersWithSnps)[1])
colnames(gm12878.promotersWithSnps.raw.ww) <- intervals
rownames(gm12878.promotersWithSnps.raw.ww) <-
     rownames(gm12878.promotersWithSnps)
for (I in intervals){
  region <- as.character(seq(I-74, I+75, 1))
  gm12878.region <- gm12878.promotersWithSnps[,region]
  gm12878.region.raw <- apply(gm12878.region, 1, raw.spectra, "WW", 10)
  cat(I, "\t", mean(gm12878.region.raw, na.rm=T), "\n")
  gm12878.promotersWithSnps.raw.ww[,which(intervals == I)] <-
     gm12878.region.raw
  plot(intervals, colMeans(gm12878.promotersWithSnps.raw.ww, na.rm=T),
     type="b", pch=19,
     ylim=range(colMeans(gm12878.promotersWithSnps.raw.ww,
     na.rm=T)[colMeans(gm12878.promotersWithSnps.raw.ww, na.rm=T)>0]))
}
# And for the reference:
hs.promotersWithSnps.raw.ww <- matrix(0, ncol=length(intervals),
     nrow=dim(hs.promotersWithSnps)[1])
colnames(hs.promotersWithSnps.raw.ww) <- intervals
rownames(hs.promotersWithSnps.raw.ww) <- rownames(hs.promotersWithSnps)
for (I in intervals){
  region <- as.character(seq(I-74, I+75, 1))
  hs.region <- hs.promotersWithSnps[,region]
  hs.region.raw <- apply(hs.region, 1, raw.spectra, "WW", 10)
  cat(I, "\t", mean(hs.region.raw, na.rm=T), "\n")
```

```
  hs.promotersWithSnps.raw.ww[,which(intervals == I)] <- hs.region.raw
  plot(intervals, colMeans(hs.promotersWithSnps.raw.ww, na.rm=T), type="b",
     pch=19, ylim=range(colMeans(hs.promotersWithSnps.raw.ww,
     na.rm=T)[colMeans(hs.promotersWithSnps.raw.ww, na.rm=T)>0]))
}
# plot them:
plot(intervals, colMeans(gm12878.promotersWithSnps.raw.ww, na.rm=T),
     type="b", pch=19)
points(intervals, colMeans(hs.promotersWithSnps.raw.ww, na.rm=T), type="b",
     pch=19, col="red")
```

The next step is to read the CAGE tags distribution around promoters for the GM12878 cell line and for other blood related cells (these will be used as reference). The ".dat" files that are used here have been generated using ChIP-Extract. They contain the CAGE distribution (raw data) around each promoter from base -100 to 100 from the TSS. The matrix contains the number of CAGE reads that map in each position (columns) around each promoter (rows). To make a fare comparison between different experiments, a normalization step is applied to the samples: first the total number of CAGE tags is scaled to 1M for each sample; then DI scores are evaluated for each promoter and for each sample and normalized using the quantile normalization.

```
# GM12878 cell line CAGE data:
files <- c("data/Gm12878Cage/gm12878CellLongPolyaRep1.dat",
           "data/Gm12878Cage/gm12878CellLongPolyaRep2.dat",
           "data/Gm12878Cage/gm12878CytosolLongPolyaRep1.dat",
           "data/Gm12878Cage/gm12878CytosolLongPolyaRep2.dat",
           "data/Gm12878Cage/gm12878NucleusLongPolyaRep1.dat",
           "data/Gm12878Cage/gm12878NucleusLongPolyaRep2.dat")
GM12878cage.DI <- matrix(nrow=length(rownames(hs.promoters)),
     ncol=length(files))
GM12878cage.AE <- matrix(nrow=length(rownames(hs.promoters)),
     ncol=length(files))
rownames(GM12878cage.DI) <- rownames(hs.promoters)
rownames(GM12878cage.AE) <- rownames(hs.promoters)
for ( I in 1:length(files)){
  GM12878cage <- matrix(0, ncol=201, nrow=length(rownames(hs.promoters)))
  rownames(GM12878cage) <- rownames(hs.promoters)
  colnames(GM12878cage) <- -100:100
  tmp <- as.matrix(read.table(files[I], row.names=1, header=F))
  GM12878cage <- tmp[rownames(GM12878cage),]
  GM12878cage <- GM12878cage*(10000000/sum(GM12878cage))
  GM12878cage.DI[,I] <- apply(GM12878cage[,50:150], 1, dispersion)
  GM12878cage.AE[,I] <- apply(GM12878cage[,50:150], 1, sum, na.rm=T)
}

# Other Blood cell derived CAGE data
files.wt <- c("data/WhiteBlood/CD20cellLongPolyaRep1.dat",
```

```
              "data/WhiteBlood/CD20cellLongPolyaRep2.dat",
              "data/WhiteBlood/CD34cellLongPolyaRep1.dat",
              "data/WhiteBlood/FantomCD4TcellDonor1.dat",
              "data/WhiteBlood/FantomCD4TcellDonor2.dat",
              "data/WhiteBlood/FantomCD4TcellDonor3.dat",
              "data/WhiteBlood/FantomCD8TcellDonor1.dat",
              "data/WhiteBlood/FantomCD8TcellDonor2.dat",
              "data/WhiteBlood/FantomCD8TcellDonor3.dat")
WBcage.DI <- matrix(nrow=length(rownames(hs.promoters)),
     ncol=length(files.wt))
WBcage.AE <- matrix(nrow=length(rownames(hs.promoters)),
     ncol=length(files.wt))
rownames(WBcage.DI) <- rownames(hs.promoters)
rownames(WBcage.AE) <- rownames(hs.promoters)
for ( I in 1:length(files.wt)){
  WBcage <- matrix(0, ncol=201, nrow=length(rownames(hs.promoters)))
  rownames(WBcage) <- rownames(hs.promoters)
  colnames(WBcage) <- -100:100
  tmp <- as.matrix(read.table(files.wt[I], row.names=1, header=F))
  WBcage <- tmp[rownames(WBcage),]
  WBcage <- WBcage*(10000000/sum(WBcage))
  WBcage.DI[,I] <- apply(WBcage[,50:150], 1, dispersion)
  WBcage.AE[,I] <- apply(WBcage[,50:150], 1, sum, na.rm=T)
}

# apply quantile normalization and get the median for each promoter:
cage.DI.norm <- normalizeQuantiles(cbind(GM12878cage.DI, WBcage.DI))
cage.AE.norm <- normalizeQuantiles(cbind(GM12878cage.AE, WBcage.AE))

GM12878cage.DI.mean <- apply(cage.DI.norm[,1:length(files)], 1, median,
     na.rm=T)
GM12878cage.AE.mean <- apply(cage.AE.norm[,1:length(files)], 1, median,
     na.rm=T)
WBcage.DI.mean <-
     apply(cage.DI.norm[,(length(files)+1):dim(cage.DI.norm)[2]], 1,
     median, na.rm=T)
WBcage.AE.mean <-
     apply(cage.AE.norm[,(length(files)+1):dim(cage.DI.norm)[2]], 1,
     median, na.rm=T)
```

The next step in the analysis is focused on evaluate the average impact of natural variants on Pol-II initiation as a function of their distance from the TSS. Intuitively, natural variants that map closer to the TSS should have a biggher impact on Pol-II activity than variants that map far away. The analysis is splitted between SNPs and Indels, since Indels should have a grater impact.

```
# Start with the Indels:
```

```
DIvsWB.indels <- vector()
AEvsWB.indels <- vector()
DIvsWB.pval.indels <- vector()
AEvsWB.pval.indels <- vector()
# Scan promoters region:
for (I in intervals){
  from <- I - 74
  to <- I + 75
  # Get the names of promoters that have NV in the region of interest
  pnames <- gm12878.indels[which(gm12878.indels[,2] >= from &
     gm12878.indels[,2] < to),1]
  # evaluate the difference in DI for the two cell lines for this promoters
  DIvsWB.indels <- c(DIvsWB.indels, mean(GM12878cage.DI.mean[pnames],
     na.rm=T) - mean(WBcage.DI.mean[pnames], na.rm=T))
  # And the same for the expression
  AEvsWB.indels <- c(AEvsWB.indels, mean(GM12878cage.AE.mean[pnames],
     na.rm=T) - mean(WBcage.AE.mean[pnames], na.rm=T))
  # Get the p.values between the two groups (GM12878 DI and other blood)
  DIpval <- t.test(GM12878cage.DI.norm[pnames,], WBcage.DI.norm[pnames,],
     alternative="g")$p.value
  # adjust the p-value for multiple testing:
  DIpval.adj <- p.adjust(DIpval, method="bonferroni", n=length(pnames))
  # Do the same for the expression:
  AEpval <- t.test(GM12878cage.AE.norm[pnames,], WBcage.AE.norm[pnames,],
     alternative="l")$p.value
  AEpval.adj <- p.adjust(AEpval, method="bonferroni", n=length(pnames))
  # save the results
  DIvsWB.pval.indels <- c(DIvsWB.pval.indels, DIpval.adj)
  AEvsWB.pval.indels <- c(AEvsWB.pval.indels, AEpval.adj)
}

# Now do the same procedure for the SNPs:
DIvsWB.snps <- vector()
AEvsWB.snps <- vector()
DIvsWB.pval.snps <- vector()
AEvsWB.pval.snps <- vector()
for (I in intervals){
  from <- I - 74
  to <- I + 75
  pnames <- gm12878.snps[which(gm12878.snps[,2] >= from & gm12878.snps[,2]
     < to),1]
  DIvsWB.snps <- c(DIvsWB.snps, mean(GM12878cage.DI.mean[pnames], na.rm=T)
     - mean(WBcage.DI.mean[pnames], na.rm=T))
  AEvsWB.snps <- c(AEvsWB.snps, mean(GM12878cage.AE.mean[pnames], na.rm=T)
     - mean(WBcage.AE.mean[pnames], na.rm=T))
```

```
  DIpval.snps <- t.test(GM12878cage.DI.norm[pnames,],
     WBcage.DI.norm[pnames,], alternative="g")$p.value
  DIpval.snps.adj <- p.adjust(DIpval.snps, method="bonferroni",
     n=length(pnames))
  AEpval.snps <- t.test(GM12878cage.AE.norm[pnames,],
     WBcage.AE.norm[pnames,], alternative="l")$p.value
  AEpval.snps.adj <- p.adjust(AEpval.snps, method="bonferroni",
     n=length(pnames))
  DIvsWB.pval.snps <- c(DIvsWB.pval.snps, DIpval.snps.adj)
  AEvsWB.pval.snps <- c(AEvsWB.pval.snps, AEpval.snps.adj)
}
```

Now it is possible to go a step further and check the linear relationship between the variation in WW frequency given by NV and the variation in DI as a function of the distance of the NV from the TSS. The procedure is similar to the previous one: scan the promoter region with a 150 bp window for NV that map there. But this time it will also check the variation in WW frequency in that region and how this corralate (using a linear model) to the variation in DI for the single promoter. Ideally, a variation in WW frequency in the N+1 region should correspond to a variation in DI. Here it is reported the code for scanning the promoter region and plotting the angular coefficients (slope) of the linear models evaluated at each position. Then it runs the same analysis again for the most important region (+110 bp from the TSS):

```
# Find the region near the TSS that has the stronghest correlation:
coeffPval <- vector()
coeffVal <- vector()
inter <- seq(-900,900,10)
for(I in inter){
  n1.region <- as.character(I)
  n1.start <- I - 74
  n1.stop <- I + 75
  n1.indels <- gm12878.indels[unique(which( (gm12878.indels[,2] >=
     n1.start) & (gm12878.indels[,2] <= n1.stop) )),1]
  hsVsGm12878inNucleosome1 <-
     gm12878.promotersWithIndels.raw.ww[n1.indels,n1.region] -
     hs.promotersWithIndels.raw.ww[n1.indels,n1.region]
  n1.snps <- gm12878.snps[unique(which( (gm12878.snps[,2] >= n1.start) &
     (gm12878.snps[,2] <= n1.stop) )),1]
  hsVsGm12878inNucleosome1.s <-
     gm12878.promotersWithSnps.raw.ww[n1.snps,n1.region] -
     hs.promotersWithSnps.raw.ww[n1.snps,n1.region]
  n1.variants <- unique(sort(c(n1.indels, n1.snps)))
  deltaNAS <- c(hsVsGm12878inNucleosome1,
     hsVsGm12878inNucleosome1.s)[n1.variants]
  deltaDI <- GM12878cage.DI.mean[n1.variants] - WBcage.DI.mean[n1.variants]
  varNas <- names(which(deltaNAS != 0))
  goodProm <- varNas
  coeffPval <- c(coeffPval,summary(lm(deltaDI[goodProm] ~
```

```
    deltaNAS[goodProm]))$coefficients[2,4])
  coeffVal <- c(coeffVal, summary(lm(deltaDI[goodProm] ~
    deltaNAS[goodProm]))$coefficients[2,1])
}

par(mfrow=c(1,1))
plot(inter, coeffVal, type="b", pch=19, xlab="Distance from TSS",
    ylab="Angular Coefficient", main="")

inter[which.min(coeffVal)]
inter[which.max(coeffVal)]

# repeat the analysis for the region with the lowest Angular Coefficient
n1.region <- "110"
n1.start <- 110 - 74
n1.stop <- 110 + 75
n1.indels <- gm12878.indels[which( (gm12878.indels[,2] >= n1.start) &
    (gm12878.indels[,2] <= n1.stop)),1]
hsVsGm12878inNucleosome1 <-
    gm12878.promotersWithIndels.raw.ww[n1.indels,n1.region] -
    hs.promotersWithIndels.raw.ww[n1.indels,n1.region]
n1.snps <- gm12878.snps[which( (gm12878.snps[,2] >= n1.start) &
    (gm12878.snps[,2] <= n1.stop)),1]
hsVsGm12878inNucleosome1.s <-
    gm12878.promotersWithSnps.raw.ww[n1.snps,n1.region] -
    hs.promotersWithSnps.raw.ww[n1.snps,n1.region]
n1.variants <- unique(sort(c(n1.indels, n1.snps)))
deltaNAS <- c(hsVsGm12878inNucleosome1,
    hsVsGm12878inNucleosome1.s)[n1.variants]
deltaDI <- GM12878cage.DI.mean[n1.variants] - WBcage.DI.mean[n1.variants]
varNas <- names(which(deltaNAS != 0))
goodProm <- varNas

diNasLm <- summary(lm(deltaDI[goodProm] ~ deltaNAS[goodProm]))
summary(lm(deltaDI[goodProm] ~ deltaNAS[goodProm]))

plot(deltaDI[goodProm] ~ deltaNAS[goodProm], pch=19)
abline(a=diNasLm$coefficients[1,1], b=diNasLm$coefficients[2,1])
```

Figures

An image of the R session derived from the code presented in the previous section, that contain all the objects needed to produce the figures can be downloaded from the ftp site et the following address:

```
ftp://ccg.vital-it.ch/dreos16/dreos16_promoterNucleosomesAnalysis.RData
```

And uploaded using the following command:

```
load("dreos16_promoterNucleosomesAnalysis.RData")
```

Figure 1

```
margins <- c(5, 4, 4, 2) + 0.1
intervals <- seq(-1000, 1000, 10)

png("Figure1.png", width=100, height=120, units="mm", res=400, pointsize=6)
####################################################################
# Panel A:
par(fig=c(0,0.32,0,1), mar=margins)
image(x=intervals, y=1:dim(hs.ww.mean)[1], t(as.matrix(hs.ww.mean)),
       col=topo.colors(100), yaxt='n', xaxt='n', ylab="", main="WW
       period.", xlab="", cex.lab=1.2)
axis(1, at=c(-1000,0,1000), labels=c("-1000bp","0","+1000bp"),
       cex.axis=1.2)
#text(x=1, y=1380, labels="WW period.", xpd=NA, srt=0, font=2, cex=1.2)
text(x=-1800, y=1350, labels="A", cex=2, font=2, xpd=NA)
par(fig=c(0.18,0.38,0,1), new=T)
image(x=1, y=1:dim(hs.ww.mean)[1], t(as.matrix(hs.tata.m)),
       col=topo.colors(100), xaxt='n', yaxt='n', ylab="", xlab="",
       cex.lab=1.2)
text(x=1.5, y=1380, labels="TATA", xpd=NA, srt=30, font=2, cex=1.2)
par(fig=c(0.24,0.44,0,1), new=T)
image(x=1, y=1:dim(hs.ww.mean)[1], t(as.matrix(hs.cpg.m)),
       col=topo.colors(100), xaxt='n', yaxt='n', ylab="", xlab="",
       cex.lab=1.2)
text(x=1.5, y=1380, labels="CpG", xpd=NA, srt=30, font=2, cex=1.2)
par(fig=c(0.3,0.62,0,1), new=T)
image(x=intervals, z=t(as.matrix(hs.nuc.mean)), col=topo.colors(100),
       yaxt='n', xaxt='n', ylab="", main="In-vivo", xlab="", cex.lab=1.2)
#text(x=1, y=1300, labels="In-vivo", xpd=NA, srt=0, font=2, cex=1.2)
axis(1, at=c(-1000,0,1000), labels=c("-1000bp","0","+1000bp"),
       cex.axis=1.2)
par(mar=c(0, 4, 0, 2)+0.1, fig=c(0,0.32,0.03,0.06), new=T, xpd=T)
image(x=seq(0, 1, l=100), y=1, z=as.matrix(seq(1, 0, l=100)),
       col=topo.colors(100), yaxt='n', xaxt='n', ylab="", main="", xlab="")
axis(1, at=c(0,1), labels=c("High","Low"), cex=0.4, padj=-0.7)
####################################################################
# Panel B:
par(fig=c(0.6,1,0.6,1), new=T, mar=margins)
```

```
plot(-1,-0.5, xlim=c(0.5,5.5), ylim=c(0,0.5), frame.plot=F, xlab="",
       ylab="Frequency", xaxt="n", yaxt="n", cex.lab=1.2)
text(x=-2, y=0.6, labels="B", cex=2, font=2, xpd=NA)
axis(2, at=seq(0,0.5,0.1), las=1, cex.axis=1.2)
axis(1, at=1:5, labels=c("Hs","Mm","Dm","Dr","Ce"), las=2, cex.axis=1.2)
# human
polygon(x=c(0.6,0.6,0.8,0.8),
       y=c(0,sum(hs.promotersDinucleotideCorrelation[,1])/dim(hs.promotersD
       inucleotideCorrelation)[1],sum(hs.promotersDinucleotideCorrelation[,
       1])/dim(hs.promotersDinucleotideCorrelation)[1],0), col="black")
polygon(x=c(0.8,0.8,1,1),
       y=c(0,sum(hs.promotersDinucleotideCorrelation[,2])/dim(hs.promotersD
       inucleotideCorrelation)[1],sum(hs.promotersDinucleotideCorrelation[,
       2])/dim(hs.promotersDinucleotideCorrelation)[1],0), col="darkred")
polygon(x=c(1,1,1.2,1.2),
       y=c(0,sum(hs.promotersDinucleotideCorrelation[,3])/dim(hs.promotersD
       inucleotideCorrelation)[1],sum(hs.promotersDinucleotideCorrelation[,
       3])/dim(hs.promotersDinucleotideCorrelation)[1],0), col="darkgreen")
polygon(x=c(1.2,1.2,1.4,1.4),
       y=c(0,sum(hs.promotersDinucleotideCorrelation[,4])/dim(hs.promotersD
       inucleotideCorrelation)[1],sum(hs.promotersDinucleotideCorrelation[,
       4])/dim(hs.promotersDinucleotideCorrelation)[1],0), col="orange")
# mouse
polygon(x=c(1.6,1.6,1.8,1.8),
       y=c(0,sum(mm.promotersDinucleotideCorrelation[,1])/dim(mm.promotersD
       inucleotideCorrelation)[1],sum(mm.promotersDinucleotideCorrelation[,
       1])/dim(mm.promotersDinucleotideCorrelation)[1],0), col="black")
polygon(x=c(1.8,1.8,2,2),
       y=c(0,sum(mm.promotersDinucleotideCorrelation[,2])/dim(mm.promotersD
       inucleotideCorrelation)[1],sum(mm.promotersDinucleotideCorrelation[,
       2])/dim(mm.promotersDinucleotideCorrelation)[1],0), col="darkred")
polygon(x=c(2,2,2.2,2.2),
       y=c(0,sum(mm.promotersDinucleotideCorrelation[,3])/dim(mm.promotersD
       inucleotideCorrelation)[1],sum(mm.promotersDinucleotideCorrelation[,
       3])/dim(mm.promotersDinucleotideCorrelation)[1],0), col="darkgreen")
polygon(x=c(2.2,2.2,2.4,2.4),
       y=c(0,sum(mm.promotersDinucleotideCorrelation[,4])/dim(mm.promotersD
       inucleotideCorrelation)[1],sum(mm.promotersDinucleotideCorrelation[,
       4])/dim(mm.promotersDinucleotideCorrelation)[1],0), col="orange")
# d. melanogaster
polygon(x=c(2.6,2.6,2.8,2.8),
       y=c(0,sum(dm.promotersDinucleotideCorrelation[,1])/dim(dm.promotersD
       inucleotideCorrelation)[1],sum(dm.promotersDinucleotideCorrelation[,
       1])/dim(dm.promotersDinucleotideCorrelation)[1],0), col="black")
polygon(x=c(2.8,2.8,3,3),
       y=c(0,sum(dm.promotersDinucleotideCorrelation[,2])/dim(dm.promotersD
       inucleotideCorrelation)[1],sum(dm.promotersDinucleotideCorrelation[,
       2])/dim(dm.promotersDinucleotideCorrelation)[1],0), col="darkred")
polygon(x=c(3,3,3.2,3.2),
       y=c(0,sum(dm.promotersDinucleotideCorrelation[,3])/dim(dm.promotersD
       inucleotideCorrelation)[1],sum(dm.promotersDinucleotideCorrelation[,
       3])/dim(dm.promotersDinucleotideCorrelation)[1],0), col="darkgreen")
polygon(x=c(3.2,3.2,3.4,3.4),
       y=c(0,sum(dm.promotersDinucleotideCorrelation[,4])/dim(dm.promotersD
       inucleotideCorrelation)[1],sum(dm.promotersDinucleotideCorrelation[,
       4])/dim(dm.promotersDinucleotideCorrelation)[1],0), col="orange")
```

```
# d. rerio
polygon(x=c(3.6,3.6,3.8,3.8),
       y=c(0,sum(dr.promotersDinucleotideCorrelation[,1])/dim(dr.promotersD
       inucleotideCorrelation)[1],sum(dr.promotersDinucleotideCorrelation[,
       1])/dim(dr.promotersDinucleotideCorrelation)[1],0), col="black")
polygon(x=c(3.8,3.8,4,4),
       y=c(0,sum(dr.promotersDinucleotideCorrelation[,2])/dim(dr.promotersD
       inucleotideCorrelation)[1],sum(dr.promotersDinucleotideCorrelation[,
       2])/dim(dr.promotersDinucleotideCorrelation)[1],0), col="darkred")
polygon(x=c(4,4,4.2,4.2),
       y=c(0,sum(dr.promotersDinucleotideCorrelation[,3])/dim(dr.promotersD
       inucleotideCorrelation)[1],sum(dr.promotersDinucleotideCorrelation[,
       3])/dim(dr.promotersDinucleotideCorrelation)[1],0), col="darkgreen")
polygon(x=c(4.2,4.2,4.4,4.4),
       y=c(0,sum(dr.promotersDinucleotideCorrelation[,4])/dim(dr.promotersD
       inucleotideCorrelation)[1],sum(dr.promotersDinucleotideCorrelation[,
       4])/dim(dr.promotersDinucleotideCorrelation)[1],0), col="orange")
# c. elegans
polygon(x=c(4.6,4.6,4.8,4.8),
       y=c(0,sum(ce.promotersDinucleotideCorrelation[,1])/dim(ce.promotersD
       inucleotideCorrelation)[1],sum(ce.promotersDinucleotideCorrelation[,
       1])/dim(ce.promotersDinucleotideCorrelation)[1],0), col="black")
polygon(x=c(4.8,4.8,5,5),
       y=c(0,sum(ce.promotersDinucleotideCorrelation[,2])/dim(ce.promotersD
       inucleotideCorrelation)[1],sum(ce.promotersDinucleotideCorrelation[,
       2])/dim(ce.promotersDinucleotideCorrelation)[1],0), col="darkred")
polygon(x=c(5,5,5.2,5.2),
       y=c(0,sum(ce.promotersDinucleotideCorrelation[,3])/dim(ce.promotersD
       inucleotideCorrelation)[1],sum(ce.promotersDinucleotideCorrelation[,
       3])/dim(ce.promotersDinucleotideCorrelation)[1],0), col="darkgreen")
polygon(x=c(5.2,5.2,5.4,5.4),
       y=c(0,sum(ce.promotersDinucleotideCorrelation[,4])/dim(ce.promotersD
       inucleotideCorrelation)[1],sum(ce.promotersDinucleotideCorrelation[,
       4])/dim(ce.promotersDinucleotideCorrelation)[1],0), col="orange")
legend("topleft",legend=c("YY","RR","WW","SS"), pch=15, pt.cex=1.5,
       bty="n", col=c("black","darkred","darkgreen","orange"), horiz=T,
       cex=0.8)
#######################################################################
# Panel C:
par(fig=c(0.6,1,0.3,0.7), new=T)
plot(1,0.5, xlim=c(0.5,5.5), ylim=c(0,1), frame.plot=F, xlab="",
       ylab="Frequency", xaxt="n", yaxt="n", cex.lab=1.2)
text(x=-2, y=1.1, labels="C", cex=2, font=2, xpd=NA)
axis(2, at=seq(0,1,0.2), las=1, cex.axis=1.2)
axis(1, at=1:5, labels=c("Hs","Mm","Dm","Dr","Ce"), las=2, cex.axis=1.2)
# human
polygon(x=c(0.7,0.7,1.3,1.3),
       y=c(0,hs.fractionConcordanceDinucleotides[1],hs.fractionConcordanceD
       inucleotides[1],0), col="lightblue")
polygon(x=c(0.7,0.7,1.3,1.3),
       y=c(hs.fractionConcordanceDinucleotides[1],hs.fractionConcordanceDin
       ucleotides[2],hs.fractionConcordanceDinucleotides[2],hs.fractionConc
       ordanceDinucleotides[1]), col="skyblue2")
polygon(x=c(0.7,0.7,1.3,1.3),
       y=c(hs.fractionConcordanceDinucleotides[2],hs.fractionConcordanceDin
```

```
       ucleotides[3],hs.fractionConcordanceDinucleotides[3],hs.fractionConc
       ordanceDinucleotides[2]), col="dodgerblue")
polygon(x=c(0.7,0.7,1.3,1.3),
       y=c(hs.fractionConcordanceDinucleotides[3],hs.fractionConcordanceDin
       ucleotides[4],hs.fractionConcordanceDinucleotides[4],hs.fractionConc
       ordanceDinucleotides[3]), col="royalblue2")
polygon(x=c(0.7,0.7,1.3,1.3),
       y=c(hs.fractionConcordanceDinucleotides[4],hs.fractionConcordanceDin
       ucleotides[5],hs.fractionConcordanceDinucleotides[5],hs.fractionConc
       ordanceDinucleotides[4]), col="blue4")
# maouse
polygon(x=c(1.7,1.7,2.3,2.3),
       y=c(0,mm.fractionConcordanceDinucleotides[1],mm.fractionConcordanceD
       inucleotides[1],0), col="lightblue")
polygon(x=c(1.7,1.7,2.3,2.3),
       y=c(mm.fractionConcordanceDinucleotides[1],mm.fractionConcordanceDin
       ucleotides[2],mm.fractionConcordanceDinucleotides[2],mm.fractionConc
       ordanceDinucleotides[1]), col="skyblue2")
polygon(x=c(1.7,1.7,2.3,2.3),
       y=c(mm.fractionConcordanceDinucleotides[2],mm.fractionConcordanceDin
       ucleotides[3],mm.fractionConcordanceDinucleotides[3],mm.fractionConc
       ordanceDinucleotides[2]), col="dodgerblue")
polygon(x=c(1.7,1.7,2.3,2.3),
       y=c(mm.fractionConcordanceDinucleotides[3],mm.fractionConcordanceDin
       ucleotides[4],mm.fractionConcordanceDinucleotides[4],mm.fractionConc
       ordanceDinucleotides[3]), col="royalblue2")
polygon(x=c(1.7,1.7,2.3,2.3),
       y=c(mm.fractionConcordanceDinucleotides[4],mm.fractionConcordanceDin
       ucleotides[5],mm.fractionConcordanceDinucleotides[5],mm.fractionConc
       ordanceDinucleotides[4]), col="blue4")
# drosophila
polygon(x=c(2.7,2.7,3.3,3.3),
       y=c(0,dm.fractionConcordanceDinucleotides[1],dm.fractionConcordanceD
       inucleotides[1],0), col="lightblue")
polygon(x=c(2.7,2.7,3.3,3.3),
       y=c(dm.fractionConcordanceDinucleotides[1],dm.fractionConcordanceDin
       ucleotides[2],dm.fractionConcordanceDinucleotides[2],dm.fractionConc
       ordanceDinucleotides[1]), col="skyblue2")
polygon(x=c(2.7,2.7,3.3,3.3),
       y=c(dm.fractionConcordanceDinucleotides[2],dm.fractionConcordanceDin
       ucleotides[3],dm.fractionConcordanceDinucleotides[3],dm.fractionConc
       ordanceDinucleotides[2]), col="dodgerblue")
polygon(x=c(2.7,2.7,3.3,3.3),
       y=c(dm.fractionConcordanceDinucleotides[3],dm.fractionConcordanceDin
       ucleotides[4],dm.fractionConcordanceDinucleotides[4],dm.fractionConc
       ordanceDinucleotides[3]), col="royalblue2")
polygon(x=c(2.7,2.7,3.3,3.3),
       y=c(dm.fractionConcordanceDinucleotides[4],dm.fractionConcordanceDin
       ucleotides[5],dm.fractionConcordanceDinucleotides[5],dm.fractionConc
       ordanceDinucleotides[4]), col="blue4")
# d. rerio
polygon(x=c(3.7,3.7,4.3,4.3),
       y=c(0,dr.fractionConcordanceDinucleotides[1],dr.fractionConcordanceD
       inucleotides[1],0), col="lightblue")
polygon(x=c(3.7,3.7,4.3,4.3),
       y=c(dr.fractionConcordanceDinucleotides[1],dr.fractionConcordanceDin
       ucleotides[2],dr.fractionConcordanceDinucleotides[2],dr.fractionConc
```

```
       ordanceDinucleotides[1]), col="skyblue2")
polygon(x=c(3.7,3.7,4.3,4.3),
       y=c(dr.fractionConcordanceDinucleotides[2],dr.fractionConcordanceDin
       ucleotides[3],dr.fractionConcordanceDinucleotides[3],dr.fractionConc
       ordanceDinucleotides[2]), col="dodgerblue")
polygon(x=c(3.7,3.7,4.3,4.3),
       y=c(dr.fractionConcordanceDinucleotides[3],dr.fractionConcordanceDin
       ucleotides[4],dr.fractionConcordanceDinucleotides[4],dr.fractionConc
       ordanceDinucleotides[3]), col="royalblue2")
polygon(x=c(3.7,3.7,4.3,4.3),
       y=c(dr.fractionConcordanceDinucleotides[4],dr.fractionConcordanceDin
       ucleotides[5],dr.fractionConcordanceDinucleotides[5],dr.fractionConc
       ordanceDinucleotides[4]), col="blue4")
# c. elegans
polygon(x=c(4.7,4.7,5.3,5.3),
       y=c(0,ce.fractionConcordanceDinucleotides[1],ce.fractionConcordanceD
       inucleotides[1],0), col="lightblue")
polygon(x=c(4.7,4.7,5.3,5.3),
       y=c(ce.fractionConcordanceDinucleotides[1],ce.fractionConcordanceDin
       ucleotides[2],ce.fractionConcordanceDinucleotides[2],ce.fractionConc
       ordanceDinucleotides[1]), col="skyblue2")
polygon(x=c(4.7,4.7,5.3,5.3),
       y=c(ce.fractionConcordanceDinucleotides[2],ce.fractionConcordanceDin
       ucleotides[3],ce.fractionConcordanceDinucleotides[3],ce.fractionConc
       ordanceDinucleotides[2]), col="dodgerblue")
polygon(x=c(4.7,4.7,5.3,5.3),
       y=c(ce.fractionConcordanceDinucleotides[3],ce.fractionConcordanceDin
       ucleotides[4],ce.fractionConcordanceDinucleotides[4],ce.fractionConc
       ordanceDinucleotides[3]), col="royalblue2")
polygon(x=c(4.7,4.7,5.3,5.3),
       y=c(ce.fractionConcordanceDinucleotides[4],ce.fractionConcordanceDin
       ucleotides[5],ce.fractionConcordanceDinucleotides[5],ce.fractionConc
       ordanceDinucleotides[4]), col="blue4")
text(x=rep(5.6, 5), y=c(0.1, 0.35, 0.65, 0.85, 0.98), labels=0:4, cex=1,
       font=1, xpd=NA)
#####################################################################
# Panel D:
par(fig=c(0.6,1,0,0.4), new=T, xpd=F)
plot(intervals,
       predict(loess(colMeans(hs.m.nucleosomes[which(rowSums(hs.promotersDi
       nucleotideCorrelation) == 4),]) ~ intervals, span=0.1)), type="l",
       main="", ylim=c(0.35,0.9), xlim=c(-500,500), frame.plot=F, ylab="In-
       vivo occupancy", xlab="", lwd=1, col="blue4", xaxt="n", las=1,
       cex.lab=1.2, cex.axis=1.2)
axis(1, at=c(-500,0,500), labels=c("-500bp","TSS","+500bp"), cex.axis=1.2)
text(x=-1000, y=0.95, labels="D", cex=2, font=2, xpd=NA)
points(intervals,
       predict(loess(colMeans(hs.m.nucleosomes[which(rowSums(hs.promotersDi
       nucleotideCorrelation) == 3),]) ~ intervals, span=0.1)), type="l",
       col="royalblue2", lwd=1)
points(intervals,
       predict(loess(colMeans(hs.m.nucleosomes[which(rowSums(hs.promotersDi
       nucleotideCorrelation) == 2),]) ~ intervals, span=0.1)), type="l",
       col="dodgerblue", lwd=1)
points(intervals,
       predict(loess(colMeans(hs.m.nucleosomes[which(rowSums(hs.promotersDi
```

```
       nucleotideCorrelation) == 1),]) ~ intervals, span=0.1)), type="l",
       col="skyblue2", lwd=1)
points(intervals,
       predict(loess(colMeans(hs.m.nucleosomes[which(rowSums(hs.promotersDi
       nucleotideCorrelation) == 0),]) ~ intervals, span=0.1)), type="l",
       col="lightblue", lwd=1)
legend("topleft", legend=c("4","3","2","1","0"), bty="n", pch=20,
       col=c("blue4","royalblue2","dodgerblue","skyblue2","lightblue"),
       cex=1)
dev.off()
```

Figure 2

```
margins <- c(5, 4, 4, 2) + 0.1

png("Figure2.png", width=178, height=60, units="mm", res=300, pointsize=6)
par(fig=c(0,0.33,0,1), mar=margins)
plot(mnaseMotifs10bp[,1] ~ optimisedMotifs10bp[,1], pch=19,
       xlab="Nucleosome +1", ylab="Genomic Nucleosomes", main="H. sapiens",
       frame.plot=F, cex.lab=1.2, cex.axis=1.2, las=1, xlim=c(0,40))
text(x=-8, y=48, labels="A", font=2, cex=2, xpd=NA)
points(mnaseMotifs10bp[ssyywwrr,1] ~ optimisedMotifs10bp[ssyywwrr,1],
       pch=19, col="magenta")
points(mnaseMotifs10bp[ssrrwwyy,1] ~ optimisedMotifs10bp[ssrrwwyy,1],
       pch=19, col="green")
legend(x=0, y=40, legend=c("SS-YY-WW-RR","SS-RR-WW-YY"),
       col=c("magenta","green"), pch=19, bty="n", xpd=NA)
###
par(fig=c(0.28,0.46,0,1), new=T)
image(x=1:5, y=1:16, z=t(mnaseOptimisedMotifs.scaled[repres,]), xaxt="n",
       yaxt="n", xlab="", ylab="", col=topo.colors(100), main="Genomic")
#text(x=8.5, y=1:16, labels=repres, cex=1, font=1, xpd=NA)
axis(1, at=1:5, labels=c("Hs","Mm","Dr","Dm","Ce"), las=3, cex.axis=1.2)
text(x=-1, y=19.5, labels="B", font=2, cex=2, xpd=NA)
points(x=c(0,0), y=c(0.5,4.5), type="l", col="magenta", lwd=3, xpd=NA)
points(x=c(0,0), y=c(4.5,8.5), type="l", col="green", lwd=3, xpd=NA)
###
par(fig=c(0.38,0.56,0,1), new=T)
image(x=1:5, y=1:16, z=t(optimisedMotifs10bp.scaled[repres,]), xaxt="n",
       yaxt="n", xlab="", ylab="", col=topo.colors(100), main="N+1")
text(x=8.5, y=1:16, labels=repres, cex=1, font=1, xpd=NA)
axis(1, at=1:5, labels=c("Hs","Mm","Dr","Dm","Ce"), las=3, cex.axis=1.2)
#points(x=c(0,0), y=c(0.5,4.5), type="l", col="red", lwd=3, xpd=NA)
#points(x=c(0,0), y=c(4.5,8.5), type="l", col="green", lwd=3, xpd=NA)
par(mar=c(0, 4, 0, 2)+0.1, fig=c(0.5,0.65,0.1,0.18), new=T, xpd=T)
image(x=seq(0, 1, l=100), y=1, z=as.matrix(seq(1, 0, l=100)),
       col=topo.colors(100), yaxt='n', xaxt='n', ylab="", main="", xlab="")
axis(1, at=c(0,1), labels=c("High","Low"), cex=0.4, padj=-0.7)
###
par(fig=c(0.66,1,0,1), new=T, mar=margins)
plot(ce.mnaseMotifs10bp[,1] ~ optimisedMotifs10bp[,5], pch=19,
       xlab="Nucleosome +1", ylab="Genomic Nucleosomes", main="C. elegans",
       frame.plot=F, cex.lab=1.2, cex.axis=1.2, las=1)
points(ce.mnaseMotifs10bp[ssyywwrr,1] ~ optimisedMotifs10bp[ssyywwrr,5],
       pch=19, col="magenta")
points(ce.mnaseMotifs10bp[ssrrwwyy,1] ~ optimisedMotifs10bp[ssrrwwyy,5],
       pch=19, col="green")
#plot(intervals, colMeans(hs.promoters.nas, na.rm=T), type="b", pch=19,
       frame.plot=F, xlab="Distance from TSS", ylab="Average strength of
       YYWWNNNRRSS", cex.lab=1.2, cex.axis=1.2, las=1)
#text(x=-1500, y=0.622, labels="C", font=2, cex=2, xpd=NA)
text(x=-3, y=33, labels="C", font=2, cex=2, xpd=NA)
dev.off()
```

Figure 3

```
X <- -103:104

png("Figure3.png", width=84, height=200, units="mm", res=300, pointsize=7)
par(fig=c(0,1,0.62,1))
plot(average.di, all.dinucleotide.di, pch=19, xlab="Dispersion index",
       ylab="Dinucleotide Strength in N+1", frame.plot=F)
points(average.di, predict(lm(all.dinucleotide.di ~ average.di)), type="l")
arrows(x0=average.di[1], y0=all.dinucleotide.di[1]-0.002, x1=average.di[1],
       y1=all.dinucleotide.di[1]-0.0007, length=0.05, angle=30, code=2,
       lwd=2, xpd=NA)
arrows(x0=average.di[11], y0=all.dinucleotide.di[11]-0.002,
       x1=average.di[11], y1=all.dinucleotide.di[11]-0.0007, length=0.05,
       angle=30, code=2, lwd=2, xpd=NA)
points(average.di, predict(lm(all.dinucleotide.di ~ average.di),
       interval=c("confidence"), level=0.99, type="response")[,2],
       type="l", lty=2)
points(average.di, predict(lm(all.dinucleotide.di ~ average.di),
       interval=c("confidence"), level=0.99, type="response")[,3],
       type="l", lty=2)
points(average.di.tata, average.dinucleotite.tata, col="red", pch=17)
legend("topright", c("TATA-","TATA+"), col=c("black","red"), pch=c(19,17),
       bty="n")
text(x=5.6, y=0.295, labels="A", xpd=NA, cex=2, font=2)
###
par(fig=c(0,1,0.3,0.68), new=TRUE)
plot(X, predict(loess(colMeans(hs.cage.micropeaks[ordered.DI[1:2000],])~X,
       span=0.05)), t="l", lwd=2, col="skyblue", xlab="Distance from TSS",
       ylab="Frequency", main="", frame.plot=F)
points(X,predict(loess(microPeaks[,"hs.all"]~X, span=0.05)),t="l",
       col="gray", lwd=2)
points(X,
       predict(loess(colMeans(hs.cage.micropeaks[ordered.DI[20001:22000],])
       ~X, span=0.05)),t="l", col="royalblue", lwd=2)
points(X,predict(loess(microPeaks[,"hs.tata"]~X, span=0.05)),t="l",
       col="darkred", lwd=2)
legend("topright", c("All","Focused","Broad","TATA+"),
       col=c("gray","skyblue","royalblue","darkred"), lty=c(1,1,1,1),
       lwd=2, bty="n")
text(x=-140, y=0.2, labels="B", xpd=NA, cex=2, font=2)
###
par(fig=c(0,0.6,0,0.36), new=TRUE)
plot(X, microPeaks.positive[["hs.pos.micropeaks"]], type="l", lwd=2,
       col="orange", xlab="Distance from TSS", ylab="Frequency", main="",
       frame.plot=F, xlim=c(-50,50))
points(X, microPeaks.positive[["hs.neg.micropeaks"]],t="l",
       col="lightgrey", lwd=2)
legend("topright", c("positive","negative"), col=c("orange","lightgrey"),
       pch=19, bty="n", cex=0.9)
text(x=-80, y=0.28, labels="C", xpd=NA, cex=2, font=2)
###
par(fig=c(0.5,1,0.1,0.36), new=TRUE)
plot(-1069:1070, microPeaks.positive[["hs.pos.m1.freq"]], type="l", lwd=2,
```

```
       col="orange", xlab="", ylab="", main="", frame.plot=F,
       xlim=c(0,200), axes=FALSE, ylim=c(0.25,0.45))
axis(2, at=c(0.3,0.4))
text(x=-60, y=0.47, labels="D", xpd=NA, cex=2, font=2)
###
par(fig=c(0.5,1,0,0.25), new=TRUE)
plot(-1069:1070, microPeaks.positive[["hs.neg.m1.freq"]], type="l", lwd=2,
       col="lightgrey", xlab="Distance from TSS", ylab="", main="",
       frame.plot=F, xlim=c(0,200), ylim=c(0.25,0.45), axes=FALSE)
axis(2, at=c(0.3,0.4))
axis(1, at=c(0, 100, 200))
dev.off()
```

Figure 4

```
indels.density <- density(gm12878.indels[,2], bw="SJ")
snp.density <- density(gm12878.snps[,2], bw="SJ")

png("Figure4.png", width=178, height=60, units="mm", res=300, pointsize=8)
par(mfrow=c(1,3))
plot(indels.density$x, indels.density$y, xlim=c(-1000,1000), xlab="Distance
       from TSS", ylab="Frequency", frame.plot=F, lwd=1,
       ylim=c(0.00015,0.00030), main="", col="darkblue", cex.axis=1.2,
       type="l")
points(snp.density$x, snp.density$y, type="l", lwd=1, col="darkred", lty=2)
text(-400, 0.00029, labels="Indels", cex=1.2)
segments(-550,0.00029, indels.density$x[which.min(abs(indels.density$x +
       650))], indels.density$y[which.min(abs(indels.density$x + 650))])
text(-600, 0.00023, labels="SNP", cex=1.2)
segments(-480,0.00023, snp.density$x[which.min(abs(snp.density$x + 200))],
       snp.density$y[which.min(abs(snp.density$x + 200))])
abline(v=0)
text(x=-1400, y=0.00033, labels="A", xpd=NA, cex=2, font=2)
##
plot(intervals, DIvsWB.indels, type="b", lwd=1, col="#e5e5ff",
       xlab="Distance from TSS", ylab="Variation in DI", frame.plot=F,
       ylim=range(DIvsWB.indels), cex.axis=1.2, xlim=c(-1000,1000))
points(intervals, DIvsWB.indels, type="p", pch=19, col="darkblue")
points(intervals, DIvsWB.snps, type="b", pch=17, col="#ffe5e5")
points(intervals, DIvsWB.snps, type="p", pch=17, col="darkred", cex=1.2)
legend("topright", legend=c("Indels","SNSs"), col=c("darkblue","darkred"),
       pch=c(19,17), bty="n")
text(x=-1400, y=10.5, labels="B", xpd=NA, cex=2, font=2)
##
plot(deltaDI[goodProm] ~ deltaNAS[goodProm], pch=20, frame.plot=F,
       xlab="Variation in N+1 affinity", ylab="Variation in DI",
       cex.axis=1.2)
abline(a=diNasLm$coefficients[1,1], b=diNasLm$coefficients[2,1])
#abline(a=1.203, b=-32.61)
#abline(a=0, b=-4.87, lwd=1)
text(x=0.29, y=1, labels="p-value = 0.022")
#text(x=0.23, y=-10, labels=expression(paste(y == -32.61, x))) #"y = -32.61
       x")
text(x=-0.45, y=46, labels="C", xpd=NA, cex=2, font=2)
dev.off()
```

Supplementary Figure 1

```
png(file="FigureS1.png", width=178, height=230, units="mm", res=300,
       pointsize=6)
#par(mfrow=c(5,4))
# H. sapiens
par(fig=c(0,0.25,0.75,1))
image(x=intervals, y=nspectra, z=t(hs.promoters.spectra.yy),
       col=topo.colors(100), xlab="", ylab="H. sapiens", main="YY",
       yaxt="n", breaks=c(seq(min(hs.promoters.spectra.yy),
       min(hs.promoters.spectra.yy["10",]), length.out=30),
       seq(min(hs.promoters.spectra.yy["10",]),
       max(hs.promoters.spectra.yy["10",]), length.out=46),
       seq(max(hs.promoters.spectra.yy["10",]),
       max(hs.promoters.spectra.yy), length.out=25)))
axis(2, at=2:20, las=1)
par(fig=c(0.25,0.5,0.75,1), new=T)
image(x=intervals, y=nspectra, z=t(hs.promoters.spectra.rr),
       col=topo.colors(100), xlab="", ylab="", main="RR", yaxt="n",
       breaks=c(seq(min(hs.promoters.spectra.rr),
       min(hs.promoters.spectra.rr["10",]), length.out=30),
       seq(min(hs.promoters.spectra.rr["10",]),
       max(hs.promoters.spectra.rr["10",]), length.out=46),
       seq(max(hs.promoters.spectra.rr["10",]),
       max(hs.promoters.spectra.rr), length.out=25)))
axis(2, at=2:20, las=1)
par(fig=c(0.5,0.75,0.75,1), new=T)
image(x=intervals, y=nspectra, z=t(hs.promoters.spectra.ww),
       col=topo.colors(100), xlab="", ylab="", main="WW", yaxt="n",
       breaks=c(seq(min(hs.promoters.spectra.ww),
       min(hs.promoters.spectra.ww["10",]), length.out=30),
       seq(min(hs.promoters.spectra.ww["10",]),
       max(hs.promoters.spectra.ww["10",]), length.out=46),
       seq(max(hs.promoters.spectra.ww["10",]),
       max(hs.promoters.spectra.ww), length.out=25)))
axis(2, at=2:20, las=1)
par(fig=c(0.75,1,0.75,1), new=T)
image(x=intervals, y=nspectra, z=t(hs.promoters.spectra.ss),
       col=topo.colors(100), xlab="", ylab="", main="SS", yaxt="n",
       breaks=c(seq(min(hs.promoters.spectra.ss),
       min(hs.promoters.spectra.ss["10",]), length.out=30),
       seq(min(hs.promoters.spectra.ss["10",]),
       max(hs.promoters.spectra.ss["10",]), length.out=46),
       seq(max(hs.promoters.spectra.ss["10",]),
       max(hs.promoters.spectra.ss), length.out=25)))
axis(2, at=2:20, las=1)
# M. musculus
par(fig=c(0,0.25,0.57,0.81), new=T)
image(x=intervals, y=nspectra, z=t(mm.promoters.spectra.yy),
       col=topo.colors(100), xlab="", ylab="M. musculus", main="",
       yaxt="n", breaks=c(seq(min(mm.promoters.spectra.yy),
       min(mm.promoters.spectra.yy["10",]), length.out=30),
       seq(min(mm.promoters.spectra.yy["10",]),
       max(mm.promoters.spectra.yy["10",]), length.out=46),
       seq(max(mm.promoters.spectra.yy["10",]),
       max(mm.promoters.spectra.yy), length.out=25)))
axis(2, at=2:20, las=1)
```

```
par(fig=c(0.25,0.5,0.57,0.81), new=T)
image(x=intervals, y=nspectra, z=t(mm.promoters.spectra.rr),
       col=topo.colors(100), xlab="", ylab="", main="", yaxt="n",
       breaks=c(seq(min(mm.promoters.spectra.rr),
       min(mm.promoters.spectra.rr["10",]), length.out=30),
       seq(min(mm.promoters.spectra.rr["10",]),
       max(mm.promoters.spectra.rr["10",]), length.out=46),
       seq(max(mm.promoters.spectra.rr["10",]),
       max(mm.promoters.spectra.rr), length.out=25)))
axis(2, at=2:20, las=1)
par(fig=c(0.5,0.75,0.57,0.81), new=T)
image(x=intervals, y=nspectra, z=t(mm.promoters.spectra.ww),
       col=topo.colors(100), xlab="", ylab="", main="", yaxt="n",
       breaks=c(seq(min(mm.promoters.spectra.ww),
       min(mm.promoters.spectra.ww["10",]), length.out=30),
       seq(min(mm.promoters.spectra.ww["10",]),
       max(mm.promoters.spectra.ww["10",]), length.out=46),
       seq(max(mm.promoters.spectra.ww["10",]),
       max(mm.promoters.spectra.ww), length.out=25)))
axis(2, at=2:20, las=1)
par(fig=c(0.75,1,0.57,0.81), new=T)
image(x=intervals, y=nspectra, z=t(mm.promoters.spectra.ss),
       col=topo.colors(100), xlab="", ylab="", main="", yaxt="n",
       breaks=c(seq(min(mm.promoters.spectra.ss),
       min(mm.promoters.spectra.ss["10",]), length.out=30),
       seq(min(mm.promoters.spectra.ss["10",]),
       max(mm.promoters.spectra.ss["10",]), length.out=46),
       seq(max(mm.promoters.spectra.ss["10",]),
       max(mm.promoters.spectra.ss), length.out=25)))
axis(2, at=2:20, las=1)
# D. rerio
par(fig=c(0,0.25,0.38,0.62), new=T)
image(x=intervals, y=nspectra, z=t(dr.promoters.spectra.yy),
       col=topo.colors(100), xlab="", ylab="D. rerio", main="", yaxt="n",
       breaks=c(seq(min(dr.promoters.spectra.yy),
       min(dr.promoters.spectra.yy["10",]), length.out=30),
       seq(min(dr.promoters.spectra.yy["10",]),
       max(dr.promoters.spectra.yy["10",]), length.out=46),
       seq(max(dr.promoters.spectra.yy["10",]),
       max(dr.promoters.spectra.yy), length.out=25)))
axis(2, at=2:20, las=1)
par(fig=c(0.25,0.5,0.38,0.62), new=T)
image(x=intervals, y=nspectra, z=t(dr.promoters.spectra.rr),
       col=topo.colors(100), xlab="", ylab="", main="", yaxt="n",
       breaks=c(seq(min(dr.promoters.spectra.rr),
       min(dr.promoters.spectra.rr["10",]), length.out=30),
       seq(min(dr.promoters.spectra.rr["10",]),
       max(dr.promoters.spectra.rr["10",]), length.out=46),
       seq(max(dr.promoters.spectra.rr["10",]),
       max(dr.promoters.spectra.rr), length.out=25)))
axis(2, at=2:20, las=1)
par(fig=c(0.5,0.75,0.38,0.62), new=T)
image(x=intervals, y=nspectra, z=t(dr.promoters.spectra.ww),
       col=topo.colors(100), xlab="", ylab="", main="", yaxt="n",
       breaks=c(seq(min(dr.promoters.spectra.ww),
       min(dr.promoters.spectra.ww["10",]), length.out=30),
```

```
        seq(min(dr.promoters.spectra.ww["10",]),
        max(dr.promoters.spectra.ww["10",]), length.out=46),
        seq(max(dr.promoters.spectra.ww["10",]),
        max(dr.promoters.spectra.ww), length.out=25)))
axis(2, at=2:20, las=1)
par(fig=c(0.75,1,0.38,0.62), new=T)
image(x=intervals, y=nspectra, z=t(dr.promoters.spectra.ss),
        col=topo.colors(100), xlab="", ylab="", main="", yaxt="n",
        breaks=c(seq(min(dr.promoters.spectra.ss),
        min(dr.promoters.spectra.ss["10",]), length.out=30),
        seq(min(dr.promoters.spectra.ss["10",]),
        max(dr.promoters.spectra.ss["10",]), length.out=46),
        seq(max(dr.promoters.spectra.ss["10",]),
        max(dr.promoters.spectra.ss), length.out=25)))
axis(2, at=2:20, las=1)
# D. melanogaster
par(fig=c(0,0.25,0.19,0.44), new=T)
image(x=intervals, y=nspectra, z=t(dm.promoters.spectra.yy),
        col=topo.colors(100), xlab="", ylab="D. melanogaster", main="",
        yaxt="n", breaks=c(seq(min(dm.promoters.spectra.yy),
        min(dm.promoters.spectra.yy["10",]), length.out=30),
        seq(min(dm.promoters.spectra.yy["10",]),
        max(dm.promoters.spectra.yy["10",]), length.out=46),
        seq(max(dm.promoters.spectra.yy["10",]),
        max(dm.promoters.spectra.yy), length.out=25)))
axis(2, at=2:20, las=1)
par(fig=c(0.25,0.5,0.19,0.44), new=T)
image(x=intervals, y=nspectra, z=t(dm.promoters.spectra.rr),
        col=topo.colors(100), xlab="", ylab="", main="", yaxt="n",
        breaks=c(seq(min(dm.promoters.spectra.rr),
        min(dm.promoters.spectra.rr["10",]), length.out=30),
        seq(min(dm.promoters.spectra.rr["10",]),
        max(dm.promoters.spectra.rr["10",]), length.out=46),
        seq(max(dm.promoters.spectra.rr["10",]),
        max(dm.promoters.spectra.rr), length.out=25)))
axis(2, at=2:20, las=1)
par(fig=c(0.5,0.75,0.19,0.44), new=T)
image(x=intervals, y=nspectra, z=t(dm.promoters.spectra.ww),
        col=topo.colors(100), xlab="", ylab="", main="", yaxt="n",
        breaks=c(seq(min(dm.promoters.spectra.ww),
        min(dm.promoters.spectra.ww["10",]), length.out=30),
        seq(min(dm.promoters.spectra.ww["10",]),
        max(dm.promoters.spectra.ww["10",]), length.out=46),
        seq(max(dm.promoters.spectra.ww["10",]),
        max(dm.promoters.spectra.ww), length.out=25)))
axis(2, at=2:20, las=1)
par(fig=c(0.75,1,0.19,0.44), new=T)
image(x=intervals, y=nspectra, z=t(dm.promoters.spectra.ss),
        col=topo.colors(100), xlab="", ylab="", main="", yaxt="n",
        breaks=c(seq(min(dm.promoters.spectra.ss),
        min(dm.promoters.spectra.ss["10",]), length.out=30),
        seq(min(dm.promoters.spectra.ss["10",]),
        max(dm.promoters.spectra.ss["10",]), length.out=46),
        seq(max(dm.promoters.spectra.ss["10",]),
        max(dm.promoters.spectra.ss), length.out=25)))
axis(2, at=2:20, las=1)
```

```
# C. elegans
par(fig=c(0,0.25,0,0.25), new=T)
image(x=intervals, y=nspectra, z=t(ce.promoters.spectra.yy),
       col=topo.colors(100), xlab="", ylab="C. elegans", main="", yaxt="n",
       breaks=c(seq(min(ce.promoters.spectra.yy),
       min(ce.promoters.spectra.yy["10",]), length.out=30),
       seq(min(ce.promoters.spectra.yy["10",]),
       max(ce.promoters.spectra.yy["10",]), length.out=46),
       seq(max(ce.promoters.spectra.yy["10",]),
       max(ce.promoters.spectra.yy), length.out=25)))
axis(2, at=2:20, las=1)
par(fig=c(0.25,0.5,0,0.25), new=T)
image(x=intervals, y=nspectra, z=t(ce.promoters.spectra.rr),
       col=topo.colors(100), xlab="", ylab="", main="", yaxt="n",
       breaks=c(seq(min(ce.promoters.spectra.rr),
       min(ce.promoters.spectra.rr["10",]), length.out=30),
       seq(min(ce.promoters.spectra.rr["10",]),
       max(ce.promoters.spectra.rr["10",]), length.out=46),
       seq(max(ce.promoters.spectra.rr["10",]),
       max(ce.promoters.spectra.rr), length.out=25)))
axis(2, at=2:20, las=1)
par(fig=c(0.5,0.75,0,0.25), new=T)
image(x=intervals, y=nspectra, z=t(ce.promoters.spectra.ww),
       col=topo.colors(100), xlab="", ylab="", main="", yaxt="n",
       breaks=c(seq(min(ce.promoters.spectra.ww),
       min(ce.promoters.spectra.ww["10",]), length.out=30),
       seq(min(ce.promoters.spectra.ww["10",]),
       max(ce.promoters.spectra.ww["10",]), length.out=46),
       seq(max(ce.promoters.spectra.ww["10",]),
       max(ce.promoters.spectra.ww), length.out=25)))
axis(2, at=2:20, las=1)
par(fig=c(0.75,1,0,0.25), new=T)
image(x=intervals, y=nspectra, z=t(ce.promoters.spectra.ss),
       col=topo.colors(100), xlab="", ylab="", main="", yaxt="n",
       breaks=c(seq(min(ce.promoters.spectra.ss),
       min(ce.promoters.spectra.ss["10",]), length.out=30),
       seq(min(ce.promoters.spectra.ss["10",]),
       max(ce.promoters.spectra.ss["10",]), length.out=46),
       seq(max(ce.promoters.spectra.ss["10",]),
       max(ce.promoters.spectra.ss), length.out=25)))
axis(2, at=2:20, las=1)
dev.off()
```

Supplementary Figure 2

```
hs.n1.spectra <- hs.promoters.spectra.yy[,"120"]
mm.n1.spectra <- mm.promoters.spectra.yy[,"120"]
dr.n1.spectra <- dr.promoters.spectra.ww[,"120"]
dm.n1.spectra <- dm.promoters.spectra.ww[,"120"]
ce.n1.spectra <- ce.promoters.spectra.ww[,"120"]

png(file="FigureS2.png", width=178, height=230, units="mm", res=300,
       pointsize=6)
par(mfrow=c(3,2))
plot(nspectra, hs.n1.spectra, pch=19, xlab="Spectrum Frequency",
       ylab="Spectrum Intensity", main="H. sapiens +1 nucleosome")
points(nspectra, predict(loess(hs.n1.spectra~nspectra, span=0.1)),
       type="l")
#
plot(nspectra, mm.n1.spectra, pch=19, xlab="Spectrum Frequency",
       ylab="Spectrum Intensity", main="M. musculus +1 nucleosome")
points(nspectra, predict(loess(mm.n1.spectra~nspectra, span=0.1)),
       type="l")
#
plot(nspectra, dr.n1.spectra, pch=19, xlab="Spectrum Frequency",
       ylab="Spectrum Intensity", main="D. rerio +1 nucleosome")
points(nspectra, predict(loess(dr.n1.spectra~nspectra, span=0.1)),
       type="l")
#
plot(nspectra, dm.n1.spectra, pch=19, xlab="Spectrum Frequency",
       ylab="Spectrum Intensity", main="D. melanogaster +1 nucleosome")
points(nspectra, predict(loess(dm.n1.spectra~nspectra, span=0.1)),
       type="l")
#
plot(nspectra, ce.n1.spectra, pch=19, xlab="Spectrum Frequency",
       ylab="Spectrum Intensity", main="C. elegans +1 nucleosome")
points(nspectra, predict(loess(ce.n1.spectra~nspectra, span=0.1)),
       type="l")
dev.off()
```

Supplementary Figure 3

```
hs.nas <- hs.promoters.spectra.rr["10.3",]
mm.nas <- mm.promoters.spectra.rr["10.3",]
dr.nas <- dr.promoters.spectra.ww["10.3",]
dm.nas <- dm.promoters.spectra.ww["10.3",]
ce.nas <- ce.promoters.spectra.ww["10.3",]

png(file="FigureS3.png", width=178, height=230, units="mm", res=300,
      pointsize=6)
par(mfrow=c(3,2))
plot(intervals, hs.nas, pch=19, xlab="Distance from TSS", ylab="NAS",
      main="H. sapiens - RR 10.3bp periodicity")
points(intervals, predict(loess(hs.nas~intervals, span=0.1)), type="l")
#
plot(intervals, mm.nas, pch=19, xlab="Distance from TSS", ylab="NAS",
      main="M. musculus - RR 10.3bp periodicity")
points(intervals, predict(loess(mm.nas~intervals, span=0.1)), type="l")
#
plot(intervals, dr.nas, pch=19, xlab="Distance from TSS", ylab="NAS",
      main="D. rerio - WW 10.3bp periodicity")
points(intervals, predict(loess(dr.nas~intervals, span=0.1)), type="l")
#
plot(intervals, dm.nas, pch=19, xlab="Distance from TSS", ylab="NAS",
      main="D. melanogaster - WW 10.3bp periodicity")
points(intervals, predict(loess(dm.nas~intervals, span=0.1)), type="l")
#
plot(intervals, ce.nas, pch=19, xlab="Distance from TSS", ylab="NAS",
      main="C. elegans - WW 10.3bp periodicity")
points(intervals, predict(loess(ce.nas~intervals, span=0.1)), type="l")
dev.off()
```

Supplementary Figure 4

```
png("FigureS4.png", width=100, height=200, units="mm", res=400,
       pointsize=6)
## M. musculus
par(fig=c(0,0.3,0.5,1))
image(x=intervals, y=1:dim(mm.ww.mean)[1], t(as.matrix(mm.ww.mean)),
       col=topo.colors(100), yaxt='n', ylab="", main="", xlab="Distance
       from TSS")
text(x=-1500, y=1180, labels="A", cex=2, font=2, xpd=NA)
text(x=1300, y=1150, labels="M. musculus", cex=1.3, font=2, xpd=NA)
text(x=0, y=1100, labels="WW periodicity", cex=1, font=2, xpd=NA)
par(fig=c(0.2,0.5,0.5,1), new=T)
image(x=intervals, y=1:dim(mm.ww.mean)[1], z=t(as.matrix(mm.nuc.mean)),
       col=topo.colors(100), yaxt='n', ylab="", main="", xlab="Distance
       from TSS")
text(x=0, y=1100, labels="In-vivo", cex=1, font=2, xpd=NA)
## D. rerio
par(fig=c(0.5,0.8,0.5,1), new=T)
image(x=intervals, y=1:dim(dr.ww.mean)[1], t(as.matrix(dr.ww.mean)),
       col=topo.colors(100), yaxt='n', ylab="", main="", xlab="Distance
       from TSS")
text(x=-1500, y=1190, labels="B", cex=2, font=2, xpd=NA)
text(x=1300, y=1170, labels="D. rerio", cex=1.3, font=2, xpd=NA)
text(x=0, y=1110, labels="WW periodicity", cex=1, font=2, xpd=NA)
par(fig=c(0.7,1,0.5,1), new=T)
image(x=intervals, y=1:dim(dr.ww.mean)[1], z=t(as.matrix(dr.nuc.mean)),
       col=topo.colors(100), yaxt='n', ylab="", main="", xlab="Distance
       from TSS")
text(x=0, y=1110, labels="In-vivo", cex=1, font=2, xpd=NA)
## D. melanogaster
par(fig=c(0,0.3,0,0.5), new=T)
image(x=intervals, y=1:dim(dm.ss.mean)[1], t(as.matrix(dm.ss.mean)),
       col=topo.colors(100), yaxt='n', ylab="", main="", xlab="Distance
       from TSS")
text(x=-1500, y=1690, labels="C", cex=2, font=2, xpd=NA)
text(x=1300, y=1630, labels="D. melanogaster", cex=1.3, font=2, xpd=NA)
text(x=0, y=1550, labels="SS periodicity", cex=1, font=2, xpd=NA)
par(fig=c(0.2,0.5,0,0.5), new=T)
image(x=intervals, y=1:dim(dm.ss.mean)[1], z=t(as.matrix(dm.nuc.mean)),
       col=topo.colors(100), yaxt='n', ylab="", main="", xlab="Distance
       from TSS")
text(x=0, y=1550, labels="In-vivo", cex=1, font=2, xpd=NA)
## C. elegans
par(fig=c(0.5,0.8,0,0.5), new=T)
image(x=intervals, y=1:dim(ce.ww.mean)[1], t(as.matrix(ce.ww.mean)),
       col=topo.colors(100), yaxt='n', ylab="", main="", xlab="Distance
       from TSS")
text(x=-1500, y=1590, labels="D", cex=2, font=2, xpd=NA)
text(x=1300, y=1530, labels="C. elegans", cex=1.3, font=2, xpd=NA)
text(x=0, y=1460, labels="WW periodicity", cex=1, font=2, xpd=NA)
par(fig=c(0.7,1,0,0.5), new=T)
```

```
image(x=intervals, y=1:dim(ce.ww.mean)[1], z=t(as.matrix(ce.nuc.mean)),
       col=topo.colors(100), yaxt='n', ylab="", main="", xlab="Distance
       from TSS")
text(x=0, y=1460, labels="In-vivo", cex=1, font=2, xpd=NA)
dev.off()
```

Supplementary Figure 5

```
intervals <- seq(-900, 900, 10)

png("FigureS5_MNase-N1_motifs.png", width=178, height=45, units="mm",
       res=300, pointsize=5)
par(mfrow=c(1,4))
plot(intervals, colMeans(hs.promoters.nas, na.rm=T), type="b", pch=19,
       frame.plot=F, xlab="Distance from TSS", ylab="Average strength of
       YYWWNNRRSS", cex.lab=1.2, cex.axis=1.2, las=1)
text(x=-1200, y=0.618, labels="A", font=2, cex=3, xpd=NA)
###
plot(mm.mnaseMotifs10bp[,1] ~ optimisedMotifs10bp[,2], pch=19,
       xlab="Nucleosome +1", ylab="Genomic Nucleosomes", main="M.
       musculus", frame.plot=F, cex.lab=1.5, cex.axis=1.5, cex.main=1.5,
       las=1)
legend("topleft", legend=c("SS-YY-WW-RR","SS-RR-WW-YY"),
       col=c("magenta","green"), pch=19, bty="n", cex=1.5)
points(mm.mnaseMotifs10bp[ssyywwrr,1] ~ optimisedMotifs10bp[ssyywwrr,2],
       pch=19, col="magenta")
points(mm.mnaseMotifs10bp[ssrrwwyy,1] ~ optimisedMotifs10bp[ssrrwwyy,2],
       pch=19, col="green")
text(x=-8, y=66, labels="B", font=2, cex=3, xpd=NA)
###
plot(dr.mnaseMotifs10bp[,1] ~ optimisedMotifs10bp[,3], pch=19,
       xlab="Nucleosome +1", ylab="Genomic Nucleosomes", main="D. rerio",
       frame.plot=F, cex.lab=1.5, cex.axis=1.5, las=1, cex.main=1.5)
points(dr.mnaseMotifs10bp[ssyywwrr,1] ~ optimisedMotifs10bp[ssyywwrr,3],
       pch=19, col="magenta")
points(dr.mnaseMotifs10bp[ssrrwwyy,1] ~ optimisedMotifs10bp[ssrrwwyy,3],
       pch=19, col="green")
text(x=-3, y=100, labels="C", font=2, cex=3, xpd=NA)
###
plot(mnaseMotifs10bp[,2] ~ optimisedMotifs10bp[,4], pch=19,
       xlab="Nucleosome +1", ylab="Genomic Nucleosomes", main="D.
       melanogaster", frame.plot=F, cex.lab=1.5, cex.axis=1.5, las=1,
       cex.main=1.5)
points(mnaseMotifs10bp[ssyywwrr,2] ~ optimisedMotifs10bp[ssyywwrr,4],
       pch=19, col="magenta")
points(mnaseMotifs10bp[ssrrwwyy,2] ~ optimisedMotifs10bp[ssrrwwyy,4],
       pch=19, col="green")
text(x=-8, y=31, labels="D", font=2, cex=3, xpd=NA)
dev.off()
```

Supplementary Figure 6

```
png("FigureS6.png",width=30, height=230, units="mm", res=300, pointsize=5)
heatmap.2(as.matrix(mnaseOptimisedMotifs),
          # dendogram control
          Rowv = TRUE,
          Colv = FALSE,
          dendrogram = "none",
          # scaling:
          scale = "column",
          # colors
          col = topo.colors(100),
          # level trace
          trace = "none",
          # color key
          key = FALSE,
          keysize = 0.1,
          mar = c(13,7)
          #
          )
dev.off()
```

Supplementary Figure 7

```
png("FigureS7.png",width=30, height=230, units="mm", res=300, pointsize=5)
heatmap.2(as.matrix(optimisedMotifs10bp),
          # dendogram control
          Rowv = TRUE,
          Colv = FALSE,
          dendrogram = "none",
          # scaling:
          scale = "column",
          # colors
          col = topo.colors(100),
          # level trace
          trace = "none",
          # color key
          key = FALSE,
          keysize = 0.1,
          mar = c(13,7)
          #
          )
dev.off()
```

Supplementary Figure 8

```
ce.h3k4me3 <- read.table("data/Ce_EPDnew_vs_GSM1208362.dat", row.names=1)
ce.nucleosomes <- read.table("data/Ce_EPDnew_vs_valouev08.out",
       row.names=1)


png("FigureS8_ceMotifsInPromoters.png", width=160, height=40, units="mm",
       res=300, pointsize=5)
par(mfrow=c(1,4))
plot(intervals, colMeans(ce.promoters.mce1.3, na.rm=T), type="l", lty=1,
       lwd=1, ylim=c(0, 0.9), frame.plot=F, xlab="Distance to TSS",
       ylab="Average intensity", col="darkblue")
points(intervals, colMeans(ce.promoters.mce2.3, na.rm=T), type="l", lty=3,
       lwd=1, col="darkblue")
points(intervals, colMeans(ce.promoters.mce1.2, na.rm=T), type="l", lty=1,
       lwd=1, col="royalblue")
points(intervals, colMeans(ce.promoters.mce2.2, na.rm=T), type="l", lty=3,
       lwd=1, col="royalblue")
points(intervals, colMeans(ce.promoters.mce1.1, na.rm=T), type="l", lty=1,
       lwd=1, col="skyblue")
points(intervals, colMeans(ce.promoters.mce2.1, na.rm=T), type="l", lty=3,
       lwd=1, col="skyblue")
text(550, 0.86, "SS-RR-WW-YY")
text(550, 0.68, "SS-YY-WW-RR")
text(x=-1200, y=1.1, labels="A", font=2, cex=2, xpd=NA)
legend("topleft", legend=c("3 MM", "2 MM", "1 MM"),
       col=c("darkblue","royalblue","skyblue"), bty="n", pt.cex=2, horiz=T,
       pch=15)
###
plot(rowMeans(ce.promoters.mce1.3[,as.character(seq(100,150,10))]),
       rowMeans(ce.promoters.mce2.3[,as.character(seq(100,150,10))]),
       type="p", pch=19, frame.plot=F, xlab="SS-RR-WW-YY intensity",
       ylab="SS-YY-WW-RR intensity")
points(rowMeans(ce.promoters.mce1.3[ce.promoters.with.mce1,as.character(seq
       (100,150,10))]),
       rowMeans(ce.promoters.mce2.3[ce.promoters.with.mce1,as.character(seq
       (100,150,10))]), type="p", pch=19, col="lightgreen")
points(rowMeans(ce.promoters.mce1.3[ce.promoters.with.mce2,as.character(seq
       (100,150,10))]),
       rowMeans(ce.promoters.mce2.3[ce.promoters.with.mce2,as.character(seq
       (100,150,10))]), type="p", pch=19, col="salmon")
text(x=-2, y=8.1, labels="B", font=2, cex=2, xpd=NA)
###
plot(seq(-990,990,10),
       colMeans(ce.nucleosomes[names(ce.promoters.with.mce1),]), type="p",
       ylim=c(0.6,1.3), xlim=c(-500,500), main="Nucleosomes",
       xlab="Distance from TSS", ylab="Average occupancy", frame.plot=F,
       pch=19, col="lightgreen")
points(seq(-990,990,10),
       colMeans(ce.nucleosomes[names(ce.promoters.with.mce2),]), type="p",
       col="salmon", pch=19)
points(seq(-990,990,10),
       predict(loess(colMeans(ce.nucleosomes[names(ce.promoters.with.mce1),
       ]) ~ seq(-990,990,10), span=0.05)), type="l", col="green")
points(seq(-990,990,10),
```

```
       predict(loess(colMeans(ce.nucleosomes[names(ce.promoters.with.mce2),
       ]) ~ seq(-990,990,10), span=0.05)), type="l", col="red")
legend("topleft", legend=c("SS-RR-WW-YY","SS-YY-WW-RR"),
       col=c("lightgreen","salmon"), pch=19, bty="n")
text(x=-800, y=1.45, labels="C", font=2, cex=2, xpd=NA)
###
plot(seq(-1000,1000,1),
       colMeans(ce.h3k4me3[names(ce.promoters.with.mce1),]), type="p",
       xlim=c(-500,500), main="H3K4me3", xlab="Distance from TSS",
       ylab="Average occupancy", frame.plot=F, pch=".", col="lightgreen")
points(seq(-1000,1000,1),
       colMeans(ce.h3k4me3[names(ce.promoters.with.mce2),]), type="p",
       col="salmon", pch=".")
points(seq(-1000,1000,1),
       predict(loess(colMeans(ce.h3k4me3[names(ce.promoters.with.mce1),]) ~
       seq(-1000,1000,1), span=0.1)), type="l", col="green")
points(seq(-1000,1000,1),
       predict(loess(colMeans(ce.h3k4me3[names(ce.promoters.with.mce2),]) ~
       seq(-1000,1000,1), span=0.1)), type="l", col="red")
text(x=-800, y=0.38, labels="D", font=2, cex=2, xpd=NA)
dev.off()
```

Supplementary Figure 9

```
png("FigureS9_correlationDiNasFigure3.png", width=100, height=100,
       units="mm", res=300, pointsize=6)
par(mfrow=c(2,2))
plot(mm.all.dinucleotide.di ~ mm.average.di, pch=19, frame.plot=F,
       ylim=c(0.25,0.30), main="M. musculus", xlab="Dispersion Index",
       ylab="Dinucleotide Strength in N+1")
points(mm.average.di, predict(lm(mm.all.dinucleotide.di ~ mm.average.di),
       interval=c("confidence"), level=0.99, type="response")[,2],
       type="l", lty=2)
points(mm.average.di, predict(lm(mm.all.dinucleotide.di ~ mm.average.di),
       interval=c("confidence"), level=0.99, type="response")[,3],
       type="l", lty=2)
points(mm.average.dinucleotite.tata ~ mm.average.di.tata, type="p", pch=17,
       col="red")
points(mm.average.di, predict(lm(mm.all.dinucleotide.di ~ mm.average.di)),
       type="l")
legend("topright", c("TATA-","TATA+"), col=c("black","red"), pch=c(19,17))
###
plot(dr.all.dinucleotide.di ~ dr.average.di, pch=19, frame.plot=F, main="D.
       rerio", xlab="Dispersion Index", ylab="Dinucleotide Strength in
       N+1")
points(dr.average.dinucleotite.tata ~ dr.average.di.tata, type="p", pch=17,
       col="red")
points(dr.average.di[-2], predict(lm(dr.all.dinucleotide.di[-2] ~
       dr.average.di[-2])), type="l")
points(dr.average.di[-2], predict(lm(dr.all.dinucleotide.di[-2] ~
       dr.average.di[-2]), interval=c("confidence"), level=0.99,
       type="response")[,2], type="l", lty=2)
points(dr.average.di[-2], predict(lm(dr.all.dinucleotide.di[-2] ~
       dr.average.di[-2]), interval=c("confidence"), level=0.99,
       type="response")[,3], type="l", lty=2)
legend(x=20, y=0.422, c("TATA-","TATA+"), col=c("black","red"),
       pch=c(19,17), xpd=NA)
###
plot(dm.all.dinucleotide.di ~ dm.average.di, pch=19, frame.plot=F, main="D.
       melanogaster", xlab="Dispersion Index", ylab="Dinucleotide Strength
       in N+1", xlim=c(5,15), ylim=c(0.28,0.32))
points(dm.average.dinucleotite.tata ~ dm.average.di.tata, type="p", pch=17,
       col="red")
points(dm.average.dinucleotite.inrdpe ~ dm.average.di.inrdpe, type="p",
       pch=15, col="blue")
points(dm.average.di, predict(lm(dm.all.dinucleotide.di ~ dm.average.di)),
       type="l")
points(dm.average.di, predict(lm(dm.all.dinucleotide.di ~ dm.average.di),
       interval=c("confidence"), level=0.99, type="response")[,2],
       type="l", lty=2)
points(dm.average.di, predict(lm(dm.all.dinucleotide.di ~ dm.average.di),
       interval=c("confidence"), level=0.99, type="response")[,3],
       type="l", lty=2)
legend("bottomright", c("TATA-","InrDPE+","TATA+"),
       col=c("black","blue","red"), pch=c(19,15,17))
###
plot(ce.all.dinucleotide.di ~ ce.average.di, ylim=c(0.34,0.45), pch=19,
```

```
       frame.plot=F, main="C. elegans", xlab="Dispersion Index",
       ylab="Dinucleotide Strength in N+1")
points(ce.average.dinucleotite.tata ~ ce.average.di.tata, type="p", pch=17,
       col="red")
points(ce.average.di, predict(lm(ce.all.dinucleotide.di ~ ce.average.di)),
       type="l")
points(ce.average.di, predict(lm(ce.all.dinucleotide.di ~ ce.average.di),
       interval=c("confidence"), level=0.99, type="response")[,2],
       type="l", lty=2)
points(ce.average.di, predict(lm(ce.all.dinucleotide.di ~ ce.average.di),
       interval=c("confidence"), level=0.99, type="response")[,3],
       type="l", lty=2)
legend("bottomright", c("TATA-","TATA+"), col=c("black","red"),
       pch=c(19,17))
dev.off()
```

Supplementary Figure 10

```
d.freq <- read.table("data/DinucleotideFrequencies.txt")
region <- as.character(seq(50, 200, 1))

mm.spec.nocpe <- spec.pgram(d.freq[region,"mm.nocpe.rr"], span=2,
       log="yes")
mm.spec.tata <- spec.pgram(d.freq[region,"mm.tata.rr"], span=2, log="yes")
mm.spec.inr <- spec.pgram(d.freq[region,"mm.inr.rr"], span=2, log="yes")
#
dr.spec.nocpe <- spec.pgram(d.freq[region,"dr.nocpe.ww"], span=2,
       log="yes")
dr.spec.tata <- spec.pgram(d.freq[region,"dr.tata.ww"], span=2, log="yes")
dr.spec.inr <- spec.pgram(d.freq[region,"dr.inr.ww"], span=2, log="yes")
#
dm.spec.nocpe <- spec.pgram(d.freq[region,"dm.nocpe.ww"], span=2,
       log="yes")
dm.spec.tata <- spec.pgram(d.freq[region,"dm.tata.ww"], span=2, log="yes")
dm.spec.inr <- spec.pgram(d.freq[region,"dm.inr.ww"], span=2, log="yes")
#
ce.spec.nocpe <- spec.pgram(d.freq[region,"ce.nocpe.ww"], span=2,
       log="yes")
ce.spec.tata <- spec.pgram(d.freq[region,"ce.tata.ww"], span=2, log="yes")
ce.spec.inr <- spec.pgram(d.freq[region,"ce.inr.ww"], span=2, log="yes")

## Mouse:
png("FigureS10.png", width = 2000, height = 1000, quality = 100,
       pointsize=28)
par(fig=c(0,0.5,0.45,1))
plot(-1074:1074, mm.nocpe.rr.freq, type="l", frame.plot=F, xlab="",
       ylab="", lwd=5, ylim=c(0.2,0.35), xlim=c(0,250), main="Dinucleotide
       pattern (M. musculus)", axes=FALSE, cex.axis=1.2, cex.main=1.5,
       cex.lab=1.2)
axis(2, at=c(0.25,0.3), lwd = 1, las=1, cex.axis=1.2)
text(x=200, y=0.33, labels="TATA- / Inr-", xpd=NA, cex=1.2)
text(x=-45, y=0.42, labels="a", font=2, xpd=NA, cex=3)
par(fig=c(0,0.5,0.22,0.75), new=TRUE)
plot(-1074:1074, mm.inr.rr.freq, type="l", frame.plot=F, ylim=c(0.2,0.35),
       xlab="", ylab="RR Frequences", main="", lwd=5, xlim=c(0,250),
       col="blue", axes=FALSE, cex.axis=1.2, cex.main=1.5, cex.lab=1.2)
axis(2, at=c(0.25,0.30), lwd = 1, las=1, cex.axis=1.2)
text(x=200, y=0.33, labels="TATA- / Inr+", xpd=NA, cex=1.2, col="blue")
par(fig=c(0,0.5,0,0.52), new=TRUE)
plot(-1074:1074, mm.tata.rr.freq, type="l", frame.plot=F, ylim=c(0.2,0.35),
       xlab="Distance from TSS", ylab="", lwd=5, xlim=c(0,250),
       col="darkred", yaxt="n", cex.axis=1.2, cex.main=1.5, cex.lab=1.2)
axis(2, at=c(0.25,0.3), lwd = 1, las=1, cex.axis=1.2)
text(x=200, y=0.33, labels="TATA+", xpd=NA, cex=1.2, col="darkred")
##
par(fig=c(0.5,1,0,1), new=TRUE)
plot(1/mm.spec.nocpe$freq,mm.spec.nocpe$spec, type="l", xlim=c(0,25),
```

```
       frame.plot="F", xlab="Period (nt)", ylab="Power spectrum", lwd=6,
       main="Spectrum decomposition (M. musculus)", cex.axis=1.2,
       cex.main=1.5, cex.lab=1.2)
points(1/mm.spec.tata$freq,mm.spec.tata$spec, type="l", lty=2, lwd=6,
       col="darkred")
points(1/mm.spec.inr$freq,mm.spec.inr$spec, type="l", lty=3, lwd=6,
       col="blue")
legend("topright",legend=c("TATA+","TATA- / Inr+","TATA- / Inr-"),
       col=c("darkred","blue","black"), lty=c(2,3,1), bty="n",
       lwd=c(4,4,4), cex=1.2)
text(x=-3.5, y=0.0023, labels="b", font=2, xpd=NA, cex=3)
dev.off()

## Zebrafish:
jpeg("SupplementaryFigure04_WWsignal_zebrafish.jpg", width = 2000, height =
       1000, quality = 100, pointsize=28)
par(fig=c(0,0.5,0.45,1))
plot(-1074:1074, dr.nocpe.ww.freq, type="l", frame.plot=F, xlab="",
       ylab="", lwd=5, ylim=c(0.29,0.43), xlim=c(0,250), main="Dinucleotide
       pattern (D. rerio)", axes=FALSE, cex.axis=1.2, cex.main=1.5,
       cex.lab=1.2)
axis(2, at=c(0.3,0.4), lwd = 1, las=1, cex.axis=1.2)
text(x=200, y=0.38, labels="TATA- / Inr-", xpd=NA, cex=1.2)
text(x=-45, y=0.5, labels="c", font=2, xpd=NA, cex=3)
par(fig=c(0,0.5,0.22,0.75), new=TRUE)
plot(-1074:1074, dr.inr.ww.freq, type="l", frame.plot=F, ylim=c(0.29,0.43),
       xlab="", ylab="WW Frequences", main="", lwd=5, xlim=c(0,250),
       col="blue", axes=FALSE, cex.axis=1.2, cex.main=1.5, cex.lab=1.2)
axis(2, at=c(0.3,0.4), lwd = 1, las=1, cex.axis=1.2)
text(x=200, y=0.37, labels="TATA- / Inr+", xpd=NA, cex=1.2, col="blue")
par(fig=c(0,0.5,0,0.52), new=TRUE)
plot(-1074:1074, dr.tata.ww.freq, type="l", frame.plot=F, ylim=c(0.29,0.4),
       xlab="Distance from TSS", ylab="", lwd=5, xlim=c(0,250),
       col="darkred", yaxt="n", cex.axis=1.2, cex.main=1.5, cex.lab=1.2)
axis(2, at=c(0.3,0.4), lwd = 1, las=1, cex.axis=1.2)
text(x=200, y=0.36, labels="TATA+", xpd=NA, cex=1.2, col="darkred")
##
par(fig=c(0.5,1,0,1), new=TRUE)
plot(1/dr.spec.nocpe$freq,dr.spec.nocpe$spec, type="l", xlim=c(0,25),
       frame.plot="F", xlab="Period (nt)", ylab="Power spectrum", lwd=6,
       main="Spectrum decomposition (D. rerio)", cex.axis=1.2,
       cex.main=1.5, cex.lab=1.2)
points(1/dr.spec.tata$freq,dr.spec.tata$spec, type="l", lty=2, lwd=6,
       col="darkred")
points(1/dr.spec.inr$freq,dr.spec.inr$spec, type="l", lty=3, lwd=6,
       col="blue")
legend("topright",legend=c("TATA+","TATA- / Inr+","TATA- / Inr-"),
       col=c("darkred","blue","black"), lty=c(2,3,1), bty="n",
       lwd=c(4,4,4), cex=1.2)
text(x=-3.5, y=0.0016, labels="d", font=2, xpd=NA, cex=3)
dev.off()

## fruitfly:
```

```
jpeg("SupplementaryFigure04_WWsignal_fruitfly.jpg", width = 2000, height =
       1000, quality = 100, pointsize=28)
par(fig=c(0,0.5,0.45,1))
plot(-1074:1074, dm.nocpe.ww.freq, type="l", frame.plot=F, xlab="",
       ylab="", lwd=5, ylim=c(0.2,0.5), xlim=c(0,250), main="Dinucleotide
       pattern (D. melanogaster)", axes=FALSE, cex.axis=1.2, cex.main=1.5,
       cex.lab=1.2)
axis(2, at=c(0.3,0.5), lwd = 1, las=1, cex.axis=1.2)
text(x=200, y=0.4, labels="TATA- / Inr-", xpd=NA, cex=1.2)
text(x=-45, y=0.65, labels="e", font=2, xpd=NA, cex=3)
par(fig=c(0,0.5,0.22,0.75), new=TRUE)
plot(-1074:1074, dm.inr.ww.freq, type="l", frame.plot=F, ylim=c(0.2,0.5),
       xlab="", ylab="WW Frequences", main="", lwd=5, xlim=c(0,250),
       col="blue", axes=FALSE, cex.axis=1.2, cex.main=1.5, cex.lab=1.2)
axis(2, at=c(0.3,0.5), lwd = 1, las=1, cex.axis=1.2)
text(x=200, y=0.4, labels="TATA- / Inr+", xpd=NA, cex=1.2, col="blue")
par(fig=c(0,0.5,0,0.52), new=TRUE)
plot(-1074:1074, dm.tata.ww.freq, type="l", frame.plot=F, ylim=c(0.22,0.5),
       xlab="Distance from TSS", ylab="", lwd=5, xlim=c(0,250),
       col="darkred", yaxt="n", cex.axis=1.2, cex.main=1.5, cex.lab=1.2)
axis(2, at=c(0.3,0.5), lwd = 1, las=1, cex.axis=1.2)
text(x=200, y=0.4, labels="TATA+", xpd=NA, cex=1.2, col="darkred")
##
par(fig=c(0.5,1,0,1), new=TRUE)
plot(1/dm.spec.nocpe$freq,dm.spec.nocpe$spec, type="l", xlim=c(0,25),
       frame.plot="F", xlab="Period (nt)", ylab="Power spectrum", lwd=6,
       main="Spectrum decomposition (D. melanogaster)", cex.axis=1.2,
       cex.main=1.5, cex.lab=1.2, ylim=c(0,0.004))
points(1/dm.spec.tata$freq,dm.spec.tata$spec, type="l", lty=2, lwd=6,
       col="darkred")
points(1/dm.spec.inr$freq,dm.spec.inr$spec, type="l", lty=3, lwd=6,
       col="blue")
legend("topright",legend=c("TATA+","TATA- / Inr+","TATA- / Inr-"),
       col=c("darkred","blue","black"), lty=c(2,3,1), bty="n",
       lwd=c(4,4,4), cex=1.2)
text(x=-3.5, y=0.0048, labels="f", font=2, xpd=NA, cex=3)
dev.off()


## worm:
jpeg("SupplementaryFigure04_WWsignal_worm.jpg", width = 2000, height =
       1000, quality = 100, pointsize=28)
par(fig=c(0,0.5,0.45,1))
plot(-1074:1074, ce.nocpe.ww.freq, type="l", frame.plot=F, xlab="",
       ylab="", lwd=5, ylim=c(0.3,0.55), xlim=c(0,250), main="Dinucleotide
       pattern (C. elegans)", axes=FALSE, cex.axis=1.2, cex.main=1.5,
       cex.lab=1.2)
axis(2, at=c(0.3,0.5), lwd = 1, las=1, cex.axis=1.2)
text(x=200, y=0.5, labels="TATA- / Inr-", xpd=NA, cex=1.2)
text(x=-45, y=0.66, labels="g", font=2, xpd=NA, cex=3)
par(fig=c(0,0.5,0.22,0.75), new=TRUE)
plot(-1074:1074, ce.inr.ww.freq, type="l", frame.plot=F, ylim=c(0.3,0.55),
       xlab="", ylab="WW Frequences", main="", lwd=5, xlim=c(0,250),
       col="blue", axes=FALSE, cex.axis=1.2, cex.main=1.5, cex.lab=1.2)
```

```
axis(2, at=c(0.3,0.5), lwd = 1, las=1, cex.axis=1.2)
text(x=200, y=0.5, labels="TATA- / Inr+", xpd=NA, cex=1.2, col="blue")
par(fig=c(0,0.5,0,0.52), new=TRUE)
plot(-1074:1074, ce.tata.ww.freq, type="l", frame.plot=F, ylim=c(0.3,0.55),
       xlab="Distance from TSS", ylab="", lwd=5, xlim=c(0,250),
       col="darkred", yaxt="n", cex.axis=1.2, cex.main=1.5, cex.lab=1.2)
axis(2, at=c(0.3,0.5), lwd = 1, las=1, cex.axis=1.2)
text(x=200, y=0.5, labels="TATA+", xpd=NA, cex=1.2, col="darkred")
##
par(fig=c(0.5,1,0,1), new=TRUE)
plot(1/ce.spec.nocpe$freq,ce.spec.nocpe$spec, type="l", xlim=c(0,25),
       frame.plot="F", xlab="Period (nt)", ylab="Power spectrum", lwd=6,
       main="Spectrum decomposition (C. elegans)", cex.axis=1.2,
       cex.main=1.5, cex.lab=1.2, ylim=c(0,0.0015))
points(1/ce.spec.tata$freq,ce.spec.tata$spec, type="l", lty=2, lwd=6,
       col="darkred")
points(1/ce.spec.inr$freq,ce.spec.inr$spec, type="l", lty=3, lwd=6,
       col="blue")
legend("topright",legend=c("TATA+","TATA- / Inr+","TATA- / Inr-"),
       col=c("darkred","blue","black"), lty=c(2,3,1), bty="n",
       lwd=c(4,4,4), cex=1.2)
text(x=-3.5, y=0.0018, labels="f", font=2, xpd=NA, cex=3)
dev.off()
```

Supplementary Figure 11

```
png("FigureS11_correlationNasAverageExpression.png", width=84, height=84,
       units="mm", res=300, pointsize=7)
plot(average.exprs, all.dinucleotide.exprs,pch=19, xlab="Average
       Expression", ylab="Dinucleotide Strength", frame.plot=F)
#points(DI.promoters.median[ordered.DI], y_sym, type="l")
points(average.exprs, predict(lm(all.dinucleotide.exprs ~ average.exprs)),
       type="l")
points(average.exprs.tata, average.dinucleotite.tata, col="red", pch=17)
dev.off()
```

Supplementary Figure 12

```
hs.cage <- as.matrix(read.table("data/Hs_EPDnew_002a_hg19_CAGEtags.dat",
      row.names=1, header=F))
X <- -103:104

hs.cage.cutoff10 <- hs.cage
hs.cage.cutoff10[hs.cage.cutoff10 > 10] <- 10

hs.cage.smooth <- predict(loess(colMeans(hs.cage)~X, span=0.05))
hs.cage.cutoff10.smooth <- predict(loess(colMeans(hs.cage.cutoff10)~X,
      span=0.05))
microPeaks.smooth <- predict(loess(microPeaks[,"hs.all"]~X, span=0.05))

png("FigureS12_MicroPeaksNorm.png", width = 2500, height = 1000, quality =
      100, pointsize=40)
par(mfrow=c(1,3))
plot(x=c(-30,-30), y=c(-1000,hs.cage.smooth[X==-30]), type="l", col="grey",
      lwd=3, frame.plot=F, main="RawData", ylab="Absolute counts",
      xlab="Distance from TSS", xlim=c(-100,100),
      ylim=range(hs.cage.smooth))
points(x=c(-20,-20), y=c(-1000,hs.cage.smooth[X==-20]), type="l",
      col="grey", lwd=3)
points(x=c(-10,-10), y=c(-1000,hs.cage.smooth[X==-10]), type="l",
      col="grey", lwd=3)
points(x=c(0,0), y=c(-1000,hs.cage.smooth[X==0]), type="l", col="grey",
      lwd=3)
points(x=c(30,30), y=c(-1000,hs.cage.smooth[X==30]), type="l", col="grey",
      lwd=3)
points(x=c(20,20), y=c(-1000,hs.cage.smooth[X==20]), type="l", col="grey",
      lwd=3)
points(x=c(10,10), y=c(-1000,hs.cage.smooth[X==10]), type="l", col="grey",
      lwd=3)
points(X, hs.cage.smooth, type="l", lwd=6)
plot(x=c(-30,-30), y=c(-1,hs.cage.cutoff10.smooth[X==-30]), type="l",
      col="grey", lwd=3, frame.plot=F, main="Count Cut-Off = 10",
      ylab="Absolute counts", xlab="Distance from TSS", xlim=c(-100,100),
      ylim=range(hs.cage.cutoff10.smooth))
points(x=c(-20,-20), y=c(-1,hs.cage.cutoff10.smooth[X==-20]), type="l",
      col="grey", lwd=3)
points(x=c(-10,-10), y=c(-1,hs.cage.cutoff10.smooth[X==-10]), type="l",
      col="grey", lwd=3)
points(x=c(0,0), y=c(-1,hs.cage.cutoff10.smooth[X==0]), type="l",
      col="grey", lwd=3)
points(x=c(30,30), y=c(-1,hs.cage.cutoff10.smooth[X==30]), type="l",
      col="grey", lwd=3)
points(x=c(20,20), y=c(-1,hs.cage.cutoff10.smooth[X==20]), type="l",
      col="grey", lwd=3)
points(x=c(10,10), y=c(-1,hs.cage.cutoff10.smooth[X==10]), type="l",
      col="grey", lwd=3)
points(X, hs.cage.cutoff10.smooth, type="l", lwd=6)
plot(x=c(-30,-30), y=c(-1,microPeaks.smooth[X==-30]), type="l", col="grey",
      lwd=3, frame.plot=F, main="Micro Peaks", ylab="Count Density",
```

```
      xlab="Distance from TSS", xlim=c(-100,100),
      ylim=range(microPeaks.smooth))
points(x=c(-20,-20), y=c(-1,microPeaks.smooth[X==-20]), type="l",
      col="grey", lwd=3)
points(x=c(-10,-10), y=c(-1,microPeaks.smooth[X==-10]), type="l",
      col="grey", lwd=3)
points(x=c(0,0), y=c(-1,microPeaks.smooth[X==0]), type="l", col="grey",
      lwd=3)
points(x=c(30,30), y=c(-1,microPeaks.smooth[X==30]), type="l", col="grey",
      lwd=3)
points(x=c(20,20), y=c(-1,microPeaks.smooth[X==20]), type="l", col="grey",
      lwd=3)
points(x=c(10,10), y=c(-1,microPeaks.smooth[X==10]), type="l", col="grey",
      lwd=3)
points(X, predict(loess(colMeans(hs.cage.micropeaks) ~ X, span=0.05)),
      t="l", lwd=6)
dev.off()
```

Supplementary Figure 13

```
X <- -103:104

png("FigureS13_CageMicroPeaks.png", width = 150, height = 150, units="mm",
       res=300, pointsize=8)
par(mfrow=c(2,2))
plot(X,
       predict(loess(colMeans(mm.cage.micropeaks[mm.ordered.DI[1:2000],])~X
       , span=0.05)), t="l", lwd=1, col="skyblue", xlab="Distance from
       TSS", ylab="Frequency", main="M. musculus - CAGE microPeaks",
       frame.plot=F)
points(X, predict(loess(microPeaks[,"mm.all"]~X, span=0.05)),t="l",
       col="gray", lwd=1)
points(X,
       predict(loess(colMeans(mm.cage.micropeaks[mm.ordered.DI[16001:18000]
       ,])~X, span=0.05)), t="l", col="royalblue")
points(X, predict(loess(microPeaks[,"mm.tata"]~X, span=0.05)),t="l",
       col="darkred", lwd=1)
#points(X,predict(loess(microPeaks[,"mm.inr"]~X, span=0.05)),t="l",
       col="blue", lwd=1)
legend("topright", c("All","Focused","Broad","TATA+"),
       col=c("gray","skyblue","royalblue","darkred"), lwd=1, bty="n")
#
plot(X,
       predict(loess(colMeans(dr.cage.micropeaks[dr.ordered.DI[1:2000],])~X
       , span=0.05)), t="l", lwd=1, col="skyblue", xlab="Distance from
       TSS", ylab="Frequency", main="D. rerio - CAGE microPeaks",
       frame.plot=F)
points(X, predict(loess(microPeaks[,"dr.all"]~X, span=0.05)),t="l",
       col="gray", lwd=1)
points(X, predict(loess(microPeaks[,"dr.tata"]~X, span=0.05)),t="l",
       col="darkred", lwd=1)
points(X,
       predict(loess(colMeans(dr.cage.micropeaks[dr.ordered.DI[6001:8000],]
       )~X, span=0.05)), t="l", col="ROYALblue", lwd=1)
legend("topright", c("All","Focused","Broad","TATA+"),
       col=c("gray","skyblue","royalblue","darkred"), lwd=1, bty="n")
#
plot(X,
       predict(loess(colMeans(dm.cage.micropeaks[dm.ordered.DI[1:2000],])~X
       , span=0.05)), t="l", lwd=1, col="skyblue", xlab="Distance from
       TSS", ylab="Frequency", main="D. melanogaster - CAGE microPeaks",
       frame.plot=F)
points(X, predict(loess(colMeans(dm.cage.micropeaks[dm.ordered.DI,])~X,
       span=0.05)), t="l", col="gray", lwd=1)
points(X,
       predict(loess(colMeans(dm.cage.micropeaks[dm.ordered.DI[4001:6000],]
       )~X, span=0.05)), t="l", col="royalblue", lwd=1)
points(X, predict(loess(microPeaks[,"dm.tata"]~X, span=0.05)),t="l",
       col="darkred", lwd=1)
points(X, predict(loess(microPeaks[,"dm.inrdpe"]~X, span=0.05)),t="l",
       col="darkgreen", lwd=1)
legend("topright", c("All","Focused","Broad","TATA+","InrDPE"),
       col=c("gray","skyblue","royalblue","darkred","darkgreen"), lwd=1,
```

```
       bty="n")
#
plot(X, predict(loess(colMeans(ce.cage.micropeaks[ce.ordered.DI[1:2000],])
       ~ X, span=0.05)), t="l", lwd=1, col="skyblue", xlab="Distance from
       TSS", ylab="Frequency", main="C. elegans - CAGE microPeaks",
       frame.plot=F)
points(X, predict(loess(microPeaks[,"ce.all"]~X, span=0.05)),t="l",
       col="gray", lwd=1)
points(X,
       predict(loess(colMeans(ce.cage.micropeaks[ce.ordered.DI[4001:6000],]
       ) ~ X, span=0.05)), t="l", col="royalblue", lwd=1)
points(X, predict(loess(microPeaks[,"ce.tata"]~X, span=0.05)),t="l",
       col="darkred", lwd=1)
legend("topright", c("All","Focused","Broad","TATA+"),
       col=c("gray","skyblue","royalblue","darkred"), lwd=1, bty="n")
dev.off()
```

Supplementary Figure 14

```
png("FigureS14.png", width=178, height=60, units="mm", res=300,
       pointsize=6)
par(mfrow=c(1,1))
plot(inter, coeffVal, type="b", pch=19, xlab="Distance from TSS",
       ylab="Angular Coefficient", main="", frame.plot=F, xaxt="n")
axis(1, at=seq(-900,900,100))
dev.off()
```

Functions definition

seq.trans

Description

Generic function for transforming a DNA sequence into integer values with the following rules: a=1; c=2; g=3; t=4; n=0.

Usage

```
seq.trans(x)
```

Arguments

x	A vector or matrix in which each element is a single base in a DNA sequence. For example a 10 bp sequence would required a 10 elements vector. Accepted values are a,c,g,t,n.

Details

The function returns an object with the same length and structure as x.

Code

```
seq.trans <- function(x){
  x[x == "a"] <- 1
  x[x == "c"] <- 2
  x[x == "g"] <- 3
  x[x == "t"] <- 4
  x[x == "n"] <- 0
  class(x) <- "numeric"
  return(x)
}
```

all.spectra

Description

Function to evaluate the frequencies strength from length 2 to 20 bp present in sequence of 150 bp length presented as a vector. It accepts 4 dinucleotides.

Usage

`all.spectra(v, type="WW")`

Arguments

`v`	Integer DNA sequence vector of length 150
`type`	Character vector specifying the dinucleotide type to be evaluated

Details

The vector `v` can be generated with the function `seq.trans` or alternatively as follow: substitute nucleotide bases with integers using the following schema: a=1; c=2; g=3; t=4; n=0. Each element of the vector corresponds to a base in the sequence.

Accepted values for `type` are: WW (A or T); SS (C or G); YY (T or C) and RR (A or G).

Code

```
all.spectra <- function(v, type="WW"){
  intervals <- seq(2,20,0.1)
  x <- v
  if (type == "WW"){
    x[which(v == 1)] <- 1 #A
    x[which(v == 4)] <- 1 #T
    x[which(v == 2)] <- 0 #C
    x[which(v == 3)] <- 0 #G
  } else if (type == "SS"){
    x[which(v == 1)] <- 0 #A
    x[which(v == 4)] <- 0 #T
    x[which(v == 2)] <- 1 #C
    x[which(v == 3)] <- 1 #G
  } else if (type == "YY"){
    x[which(v == 1)] <- 0 #A
    x[which(v == 4)] <- 1 #T
    x[which(v == 2)] <- 1 #C
    x[which(v == 3)] <- 0 #G
  } else if (type == "RR"){
    x[which(v == 1)] <- 1 #A
    x[which(v == 4)] <- 0 #T
    x[which(v == 2)] <- 0 #C
    x[which(v == 3)] <- 1 #G
  }
  # If there are no hits in the sequence then result is 0
  if (sum(which(is.na(x)) > 0) > 0) {
    result <- 0
```

```
  } else {
    # Evaluate dinucleotides
    x.dn <- x[1:(length(x)-1)] + x[2:(length(x))]
    x.dn[which(x.dn != 2)] <- 0
    x.dn[which(x.dn == 2)] <- 1
    x.s <- spec.pgram(x.dn, log="yes", plot = FALSE)
    # Extract value corresponding to all frequencies (intervals)
    freq <- 1/x.s$freq
    spec.all <- predict(loess(x.s$spec ~ freq, span=0.07),
newdata=data.frame(freq=intervals))
    result <- spec.all
  }
  return(result)
}
```

raw.spectra

Description

General function used to evaluate the spectrum intensity for a given frequency in sequences of 150 bp in length. It accepts 4 dinucleotide types.

Usage

`spec.wrap(v, type=”WW”, freq=10)`

Arguments

`v`	Integer DNA sequence vector of length 150
`type`	Character vector specifying the dinucleotide type to be evaluated
`freq`	Numeric vector with the frequency value to be evaluated.

Details

The vector `v` can be generated with the function `seq.trans` or alternatively as follow: substitute nucleotide bases with integers using the following schema: a=1; c=2; g=3; t=4; n=0. Each element of the vector corresponds to a base in the sequence.

Accepted values for `type` are: WW (A or T); SS (C or G); YY (T or C) and RR (A or G)

The `freq` vector should a positive integer that is equivalent to `1/p` where `p` is the period calculated by the Fourier transform.

Code

```
raw.spectra <- function(v, type="WW", freq=10){
  x <- v
  if (type == "WW"){
    x[which(v == 1)] <- 1 #A
    x[which(v == 4)] <- 1 #T
    x[which(v == 2)] <- 0 #C
    x[which(v == 3)] <- 0 #G
  } else if (type == "SS"){
    x[which(v == 1)] <- 0 #A
    x[which(v == 4)] <- 0 #T
    x[which(v == 2)] <- 1 #C
    x[which(v == 3)] <- 1 #G
  } else if (type == "YY"){
    x[which(v == 1)] <- 0 #A
    x[which(v == 4)] <- 1 #T
    x[which(v == 2)] <- 1 #C
    x[which(v == 3)] <- 0 #G
  } else if (type == "RR"){
    x[which(v == 1)] <- 1 #A
    x[which(v == 4)] <- 0 #T
    x[which(v == 2)] <- 0 #C
    x[which(v == 3)] <- 1 #G
```

```
  }
  # If there are no hits in the sequence then result is 0
  if (sum(which(is.na(x)) > 0) > 0) {
    result <- 0
  } else {
    # Evaluate dinucleostides
    x.dn <- x[1:(length(x)-1)] + x[2:(length(x))]
    x.dn[which(x.dn != 2)] <- 0
    x.dn[which(x.dn == 2)] <- 1
    # Extract simulated bg values corresponding to the same dinucleot. freq
    if (sum(x) == 0 | sum(x) == 150){
      bg = 1
    }else{
      bg <- 1
    }
    p1 <- 1/freq
    spec.all <- spec.pgram(x.dn, log="yes", plot = FALSE)
    result <- spec.all$spec[ which.min(abs(p1 - spec.all$freq))] / bg
  }
  return(result)
}
```

find.freq

Description

Function that evaluates average dinucleotide frequencies in a group of sequences of the same length.

Usage

```
find.freq(mat, type)
```

Arguments

`mat`	Integer DNA sequence matrix
`type`	Character vector specifying the dinucleotide type to be evaluated

Details

The matrix `mat` can be generated with the function `seq.trans` or alternatively as follow: substitute nucleotide bases with integers using the following schema: a=1; c=2; g=3; t=4; n=0. Each element of the matrix corresponds to a base in the sequence.

Accepted values for `type` are: WW (A or T); SS (C or G); YY (T or C) and RR (A or G)

Code

```
find.freq <- function(mat, type="WW"){
      x <- mat
      if (type == "WW"){
            x[which(mat == 1)] <- 1 #A
            x[which(mat == 4)] <- 1 #T
            x[which(mat == 2)] <- 0 #C
            x[which(mat == 3)] <- 0 #G
      } else if (type == "SS"){
            x[which(mat == 1)] <- 0 #A
            x[which(mat == 4)] <- 0 #T
            x[which(mat == 2)] <- 1 #C
            x[which(mat == 3)] <- 1 #G
      } else if (type == "YY"){
            x[which(mat == 1)] <- 0 #A
            x[which(mat == 4)] <- 1 #T
            x[which(mat == 2)] <- 1 #C
            x[which(mat == 3)] <- 0 #G
      } else if (type == "RR"){
            x[which(mat == 1)] <- 1 #A
            x[which(mat == 4)] <- 0 #T
            x[which(mat == 2)] <- 0 #C
            x[which(mat == 3)] <- 1 #G
      }
      x.dn <- x[,1:(dim(x)[2]-1)] + x[,2:(dim(x)[2])]
      x.dn[which(x.dn != 2)] <- 0
```

```
      x.dn[which(x.dn == 2)] <- 1
      return(colMeans(x.dn))
}
```

dispersion

Description

General function used to evaluate the Dispersion Index (DI) of promoters using CAGE tag distributions around promoters as input.

Usage

```
dispersion(x)
```

Arguments

`x`	An integer vector representing tag count mapped around transcription start sites.

Details

Each vector element of `x` is the number of tags (5' end) that map to the specific location. The steps between elements (1 base) should remain constant. The transcription start site must be positioned at the mid-element in the vector.

The function scans vector `x` and calculate the spread of CAGE tags around the mid-element of the vector (for example if vector length is 201, the function uses the 101 element as central) using the following formula:

$$DI_k = \sqrt{\frac{\sum_{i=1}^{N}(x_i - \overline{x})^2}{N}}$$

Where *N* is the total number of tag starts in the window around promoter *k*, and x_i is the mapped position of the 5' end of tag *i*.

Code

```
dispersion <- function(x){
  if(sum(x) <= 2) {
    m <- 0
    s <- NA
  }
  else {
    m <- sum(x*(1:length(x)))/sum(x)
    s <- (sum((x*(1:length(x)-m)**2))/sum(x))**0.5
  }
  return(s)
}
```

rawSpectraMappedSeq

Description

General function used to find the spectral intensity in a Boolean vector for a specified frequency.

Usage

```
spec.wrap(v, freq=10)
```

Arguments

`v`	Integer DNA sequence vector of length 150
`freq`	Numeric vector with the frequency value to be evaluated.

Details

This function is similar to `raw.spectra` since it evaluates the spectral intensity for a given frequency in a vector of 150 bp. The only difference is that it acceps a Boolean vector as input that derive from the mapping of a motif (any motif) in the sequence

Code

```
rawSpectraMappedSeq <- function(v, freq=10){
  x <- v
  # If there are no hits in the sequence then result is 0
  if (sum(which(is.na(x)) > 0) > 0) {
    result <- 0
  } else {
    # Extract simulated bg values corresponding to the same dinucleotide
freq
    if (sum(x) == 0 | sum(x) == 150){
      bg <- 1
    }else{
      bg <- 1
    }
    p1 <- 1/freq
    spec.all <- spec.pgram(x, log="yes", plot = FALSE)
    result <- spec.all$spec[ which.min(abs(p1 - spec.all$freq))] / bg
  }
  return(result)
}
```

condense.matrix

Description

General function used calculate the column average in a group of rows in a matrix

Usage

`condense.matrix(mat, rows=20)`

Arguments

`mat`	An integer matrix
`rows`	Integer specifying the number of rows that have to be averaged

Details

The function takes groups of rows of length `rows` in the matrix `mat` and evaluates the column averages for each group. It returns a matrix with rows numbers reduced by a factor equal to `rows`.

Code

```
condense.matrix <- function(mat, rows=20){
  groups <- sort(rep(1:ceiling(dim(mat)[1]/rows), rows))[1:dim(mat)[1]]
  c.mat <- matrix(ncol=dim(mat)[2], nrow=max(groups))
  for (I in 1:dim(mat)[2]){
    c.mat[,I] <- tapply(mat[,I], groups, mean, na.rm=T)
  }
  return(c.mat)
}
```

find.peaks

Description

General function used to find peaks in CAGE data

Usage

`find.peaks(x, win, th)`

Arguments

`x`	An integer vector representing tag count mapped to a genomic location.
`win`	Integer with the window used for peak finding.
`th`	Integer with threshold to be apply for peak finding

Details

Each vector element of `x` is the number of tags (5' end) that map to the specific location. The steps between elements (e.g. 1 base) should remain constant.

The window parameter `win` specify the number of consecutive elements of `x` that are used for peak finding. Please be aware that `win` represent actual bases distance only if the step used in `x` is 1.

The threshold `th` is the minimum number of tags that a segment of length `win` must have in order for it to be considered as a peak

The function scans vector `x` for non-overlapping tag enriched regions of length `win` with tag count higher than `th`. It returns an integer vector in which only peak center positions (defined as the position with the higher tag count in the region of length `win`) have values grater that 0 and shows the total number of tags mapped in the region. All other vector elements are equal to 0.

Code

```
find.peaks <- function(x, win, th) {
      L <- length(x)
      y <- rep(0,L)
      b <- floor((win-1)/2)
      e <- floor(win/2)
      for(i in (1+b):(L-e)) {
            if(x[i] > 0) {
                  y[i] <- sum(x[(i-b):(i+e)])
            }
      }
      z <- rep(0,L)
      for(i in (b+win):(L-win-e+1)) {
            if(y[i] >= th & y[i] >  max(y[(i-win+1):(i-1)]) & y[i] >=
            max(y[(i+1):(i+win-1)])) {
                  z[i] <- y[i]
            } else {
                  z[i] <- 0
            }
```

```
	}
	y <- z
	z <- rep(0,L)
	for(i in which(y >= 1)) {
		range <- (i-win+1):(i+win-1)
		range <- (i-b):(i+e)
		j <- round(sum(x[range]*range)/sum(x[range]))
		z[j] <- y[i]
	}
	return(z)
}
```

References:

1. Dreos, R., Ambrosini, G., Cavin Perier, R. and Bucher, P. (2013) EPD and EPDnew, high-quality promoter resources in the next-generation sequencing era. *Nucleic acids research*, **41**, D157-164.

2. Karolchik, D., Hinrichs, A.S., Furey, T.S., Roskin, K.M., Sugnet, C.W., Haussler, D. and Kent, W.J. (2004) The UCSC Table Browser data retrieval tool. *Nucleic acids research*, **32**, D493-496.

3. Djebali, S., Davis, C.A., Merkel, A., Dobin, A., Lassmann, T., Mortazavi, A., Tanzer, A., Lagarde, J., Lin, W. and Schlesinger, F. (2012) Landscape of transcription in human cells. *Nature*, **489**, 101-108.

4. Forrest, A.R., Kawaji, H., Rehli, M., Baillie, J.K., de Hoon, M.J., Haberle, V., Lassman, T., Kulakovskiy, I.V., Lizio, M., Itoh, M. *et al.* (2014) A promoter-level mammalian expression atlas. *Nature*, **507**, 462-470.

5. Nepal, C., Hadzhiev, Y., Previti, C., Haberle, V., Li, N., Takahashi, H., Suzuki, A.M., Sheng, Y., Abdelhamid, R.F., Anand, S. *et al.* (2013) Dynamic regulation of the transcription initiation landscape at single nucleotide resolution during vertebrate embryogenesis. *Genome research*, **23**, 1938-1950.

6. Kruesi, W.S., Core, L.J., Waters, C.T., Lis, J.T. and Meyer, B.J. (2013) Condensin controls recruitment of RNA polymerase II to achieve nematode X-chromosome dosage compensation. *eLife*, **2**, e00808.

7. Gaffney, D.J., McVicker, G., Pai, A.A., Fondufe-Mittendorf, Y.N., Lewellen, N., Michelini, K., Widom, J., Gilad, Y. and Pritchard, J.K. (2012) Controls of nucleosome positioning in the human genome. *PLoS Genet*, **8**, e1003036.

8. van Oevelen, C., Collombet, S., Vicent, G., Hoogenkamp, M., Lepoivre, C., Badeaux, A., Bussmann, L., Sardina, J.L., Thieffry, D., Beato, M. *et al.* (2015) C/EBPalpha Activates Pre-existing and De Novo Macrophage Enhancers during Induced Pre-B Cell Transdifferentiation and Myelopoiesis. *Stem cell reports*, **5**, 232-247.

9. Zhang, Y., Vastenhouw, N.L., Feng, J., Fu, K., Wang, C., Ge, Y., Pauli, A., van Hummelen, P., Schier, A.F. and Liu, X.S. (2014) Canonical nucleosome organization at promoters forms during genome activation. *Genome research*, **24**, 260-266.

10. Bohla, D., Herold, M., Panzer, I., Buxa, M.K., Ali, T., Demmers, J., Kruger, M., Scharfe, M., Jarek, M., Bartkuhn, M. *et al.* (2014) A functional insulator screen identifies NURF and dREAM components to be required for enhancer-blocking. *PloS one*, **9**, e107765.

11. Valouev, A., Ichikawa, J., Tonthat, T., Stuart, J., Ranade, S., Peckham, H., Zeng, K., Malek, J.A., Costa, G., McKernan, K. *et al.* (2008) A high-resolution, nucleosome position map of C. elegans reveals a lack of universal sequence-dictated positioning. *Genome research*, **18**, 1051-1063.

12. Bucher, P. (1990) Weight matrix descriptions of four eukaryotic RNA polymerase II promoter elements derived from 502 unrelated promoter sequences. *Journal of molecular biology*, **212**, 563-578.

13. Charif, D. and Lobry, J.R. (2007), *Structural approaches to sequence evolution: Molecules, networks, populations*. Springer Verlag, New York, pp. 207-232.
