# Supplementary figures and images for: Influence of Rotational Nucleosome Positioning on Transcription Start Site Selection in Animal Promoters
Source: PLoS Comput Biol. 2016 Oct 7;12(10):e1005144. doi: 10.1371/journal.pcbi.1005144 (PMC5055345; doi:10.1371/journal.pcbi.1005144)

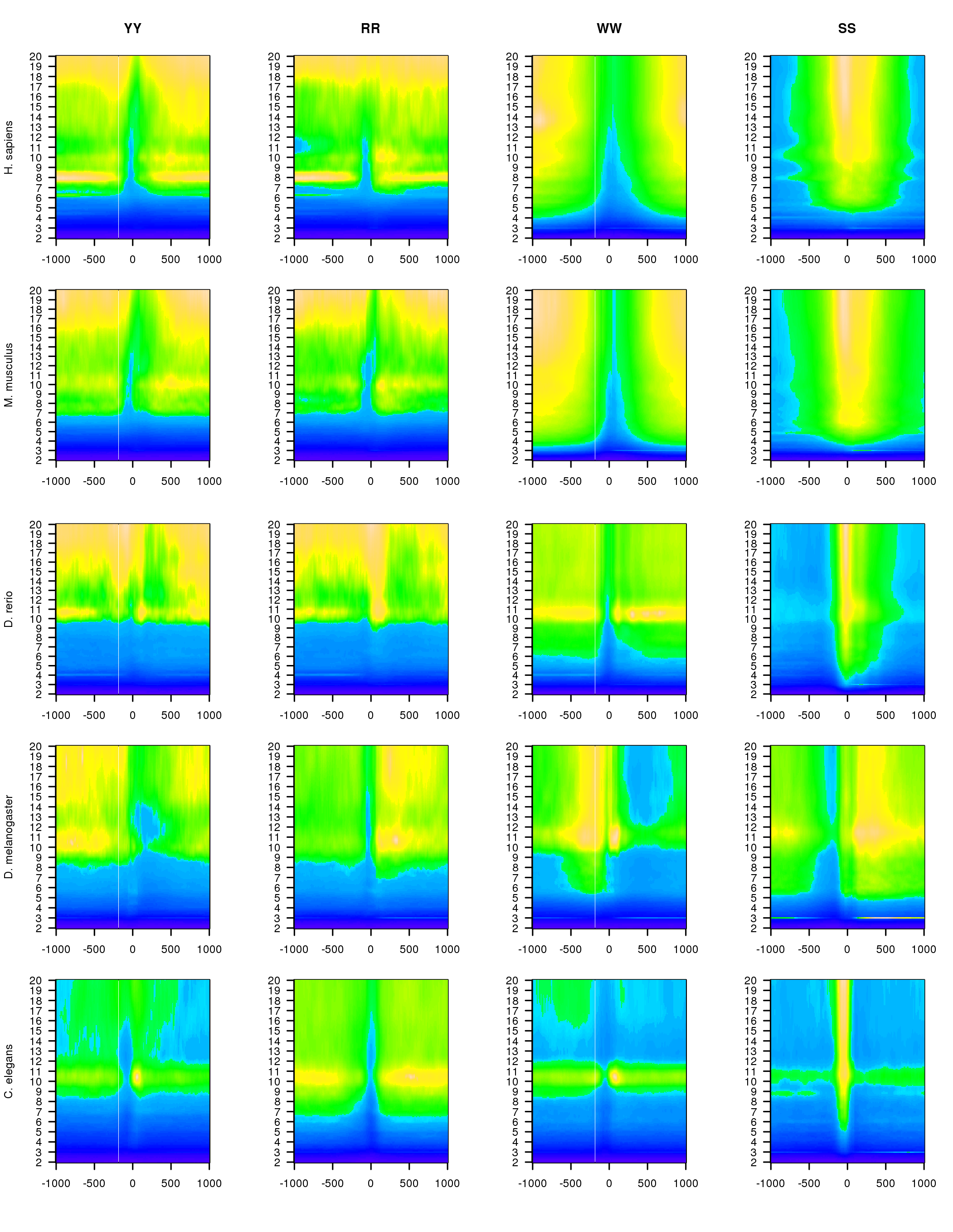

Supplement: S1 Fig — 2 kb region around promoters were scanned with a sliding window of 150 bp and 10 bp shift for the intensity of dinucleotide frequencies of period 2 to 20 bp. For each region, the average frequency intensities across all promoters were plotted against the distance of the region to the TSS. All organisms have a peak of signal intensity in correspondence to a period of 10–11 bp in agreement with the notion that this frequency helps the DNA wrapping around the histone octamer. (PNG) [file pcbi.1005144.s002.png]

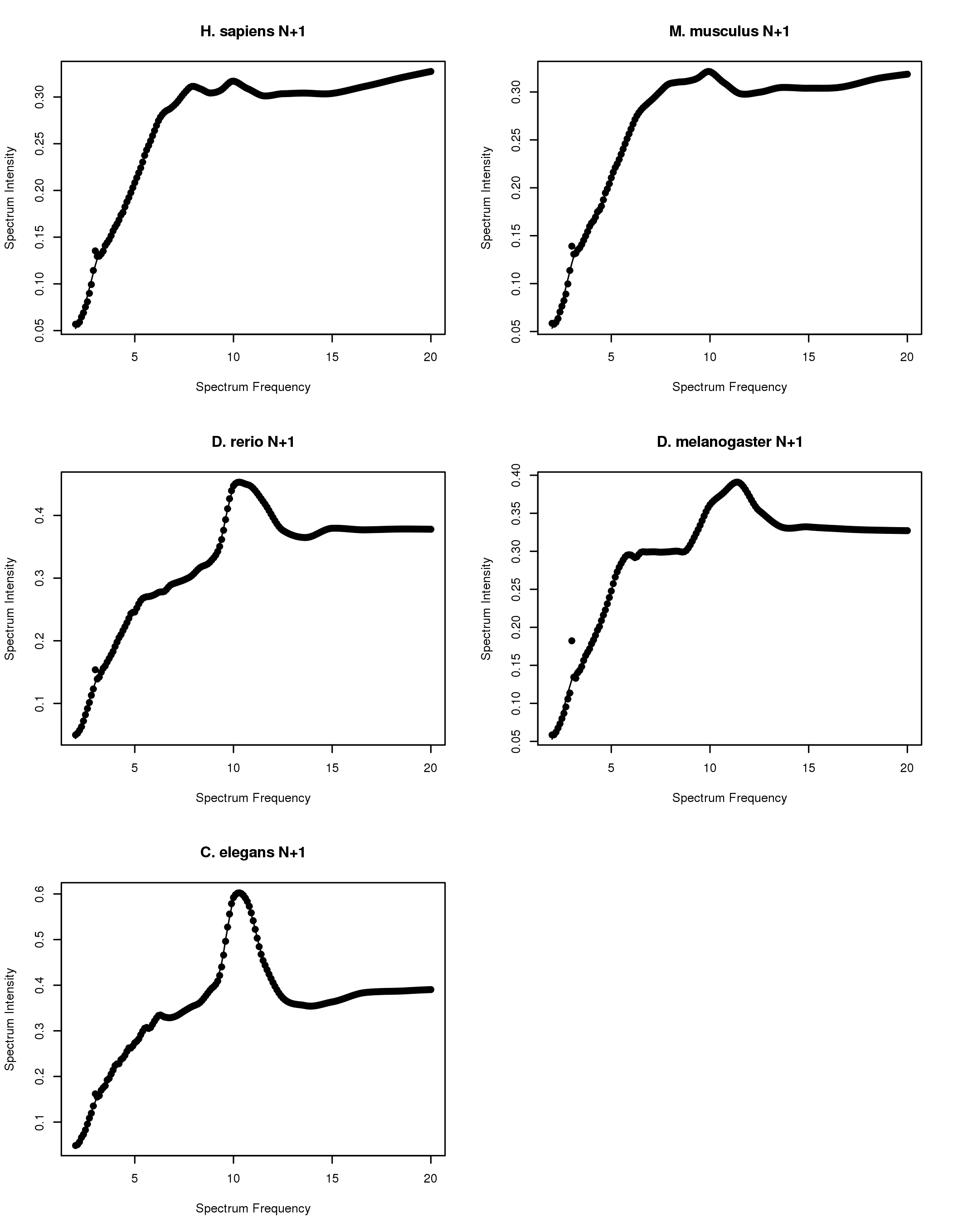

Supplement: S2 Fig — Average spectrum intensities for selected dinucleotides evaluated at position +120 bp from the TSS. Each organism shows a peak in correspondence of 10–11 bp frequency. Dinucleotide selected: H. sapiens and M. musculus: RR; D. rerio, D. melanogaster and C. elegans: WW (PNG) [file pcbi.1005144.s003.png]

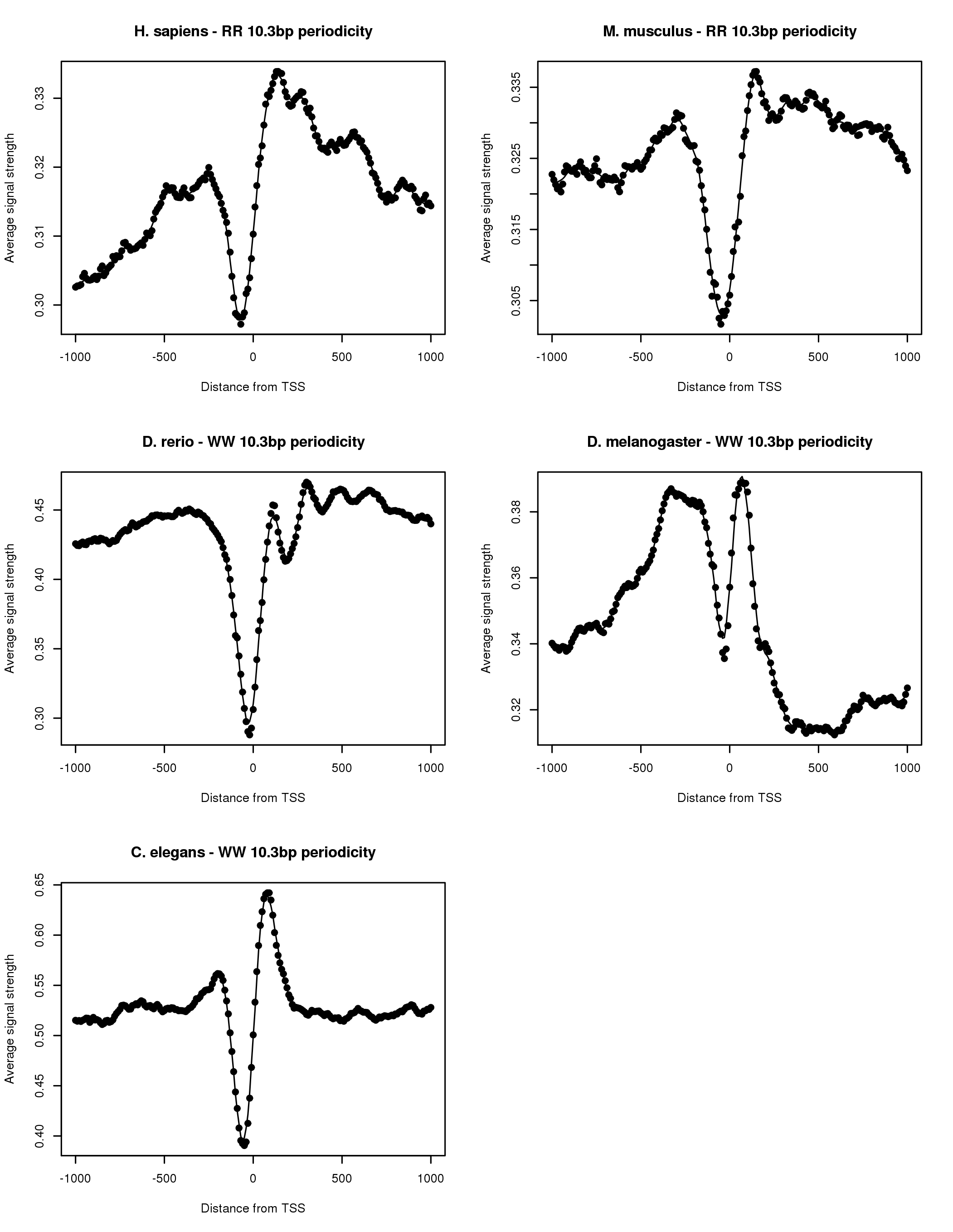

Supplement: S3 Fig — The average intensity of 10 bp frequency for selected dinucleotides in a 2 Kb region around animal promoters. All organisms show signal depletion immediately upstream the TSS followed by a peak downstream. (PNG) [file pcbi.1005144.s004.png]

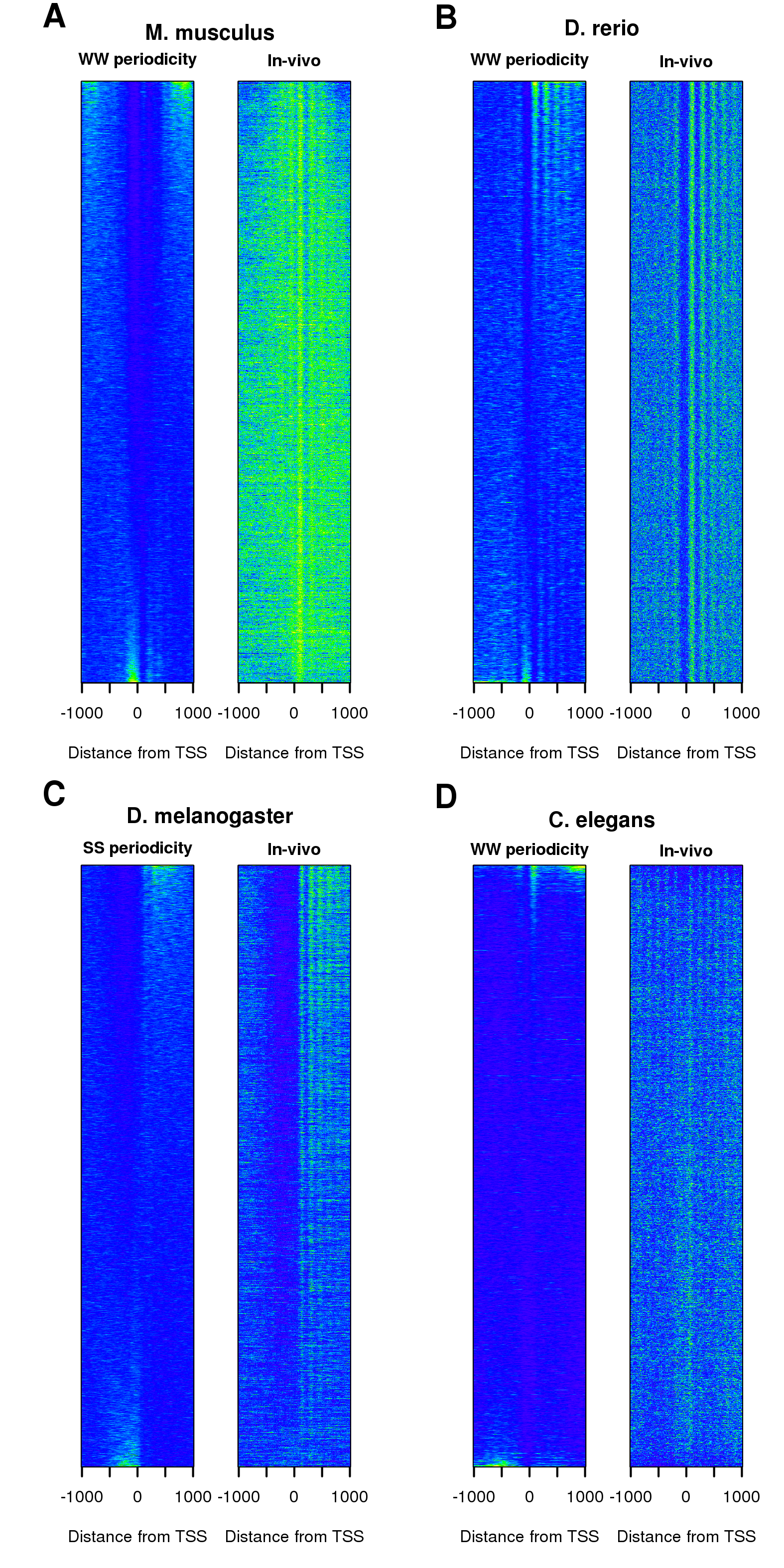

Supplement: S4 Fig — Intensity of a 10 bp dinucleotide periodicity calculated using a Fourier transform in a sliding window of 150 bp on a 2 kb region around animal promoters compared to the in-vivo nucleosome occupancy profiles derived from MNase-seq reads counts in the same region. Promoters were ordered according to their correlation between the intensity of the 10 bp dinucleotide signal and the average in-vitro nucleosome distribution in the same region. (PNG) [file pcbi.1005144.s005.png]

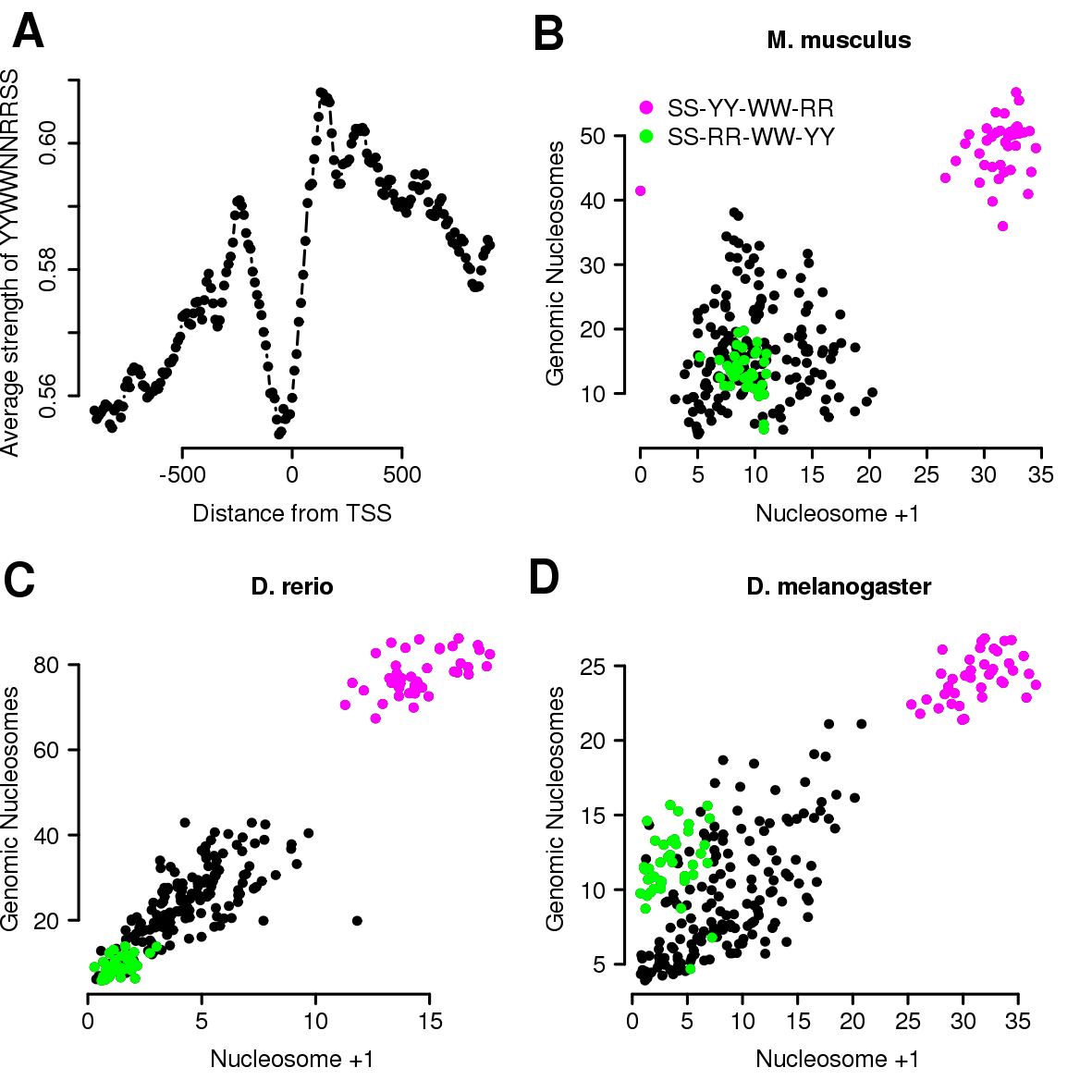

Supplement: S5 Fig — (A) Average 10 bp frequency intensity of the consensus sequence YYWWNNRRSS (3 mismatches allowed) around H. sapiens promoters. (B) Correlation between 10 bp long dinucleotide patterns composed of one copy of each SS, WW, YY and RR dinucleotides and 2 Ns evaluated on M. musculus genomic nucleosomes and the N+1 nucleosome; each dot represents the strength of the 10 bp frequency of a consensus sequence in the N+1 region or in genomic nucleosome regions (defined by MNase-seq data); green and red dots mark two classes of patterns (circular permutations of SS-RR-WW-YY and SS-YY-WW-RR, respectively) that are known to have high nucleosomes affinities. (C) As B) but for D. rerio promoters and genomic nucleosomes. (D) As for (B) but for D. melanogaster promoters and genomic nucleosomes. (PNG) [file pcbi.1005144.s006.png]

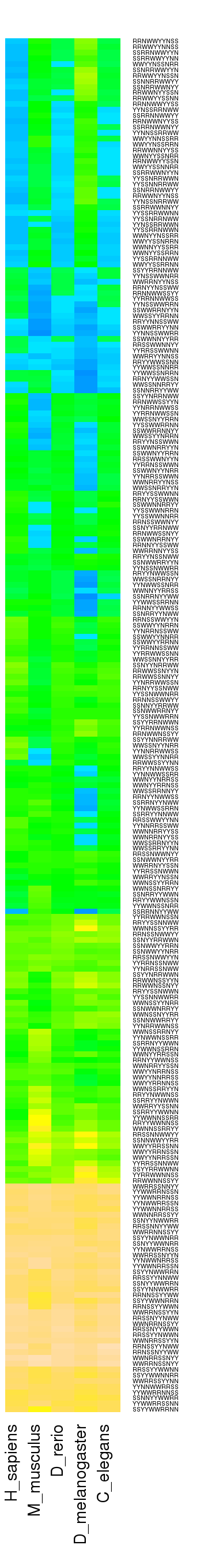

Supplement: S6 Fig — 10 bp frequency intensity for the 240 randomly generated sequences in MNase-seq defined genomic nucleosomes for the 5 organisms under study. (PNG) [file pcbi.1005144.s007.png]

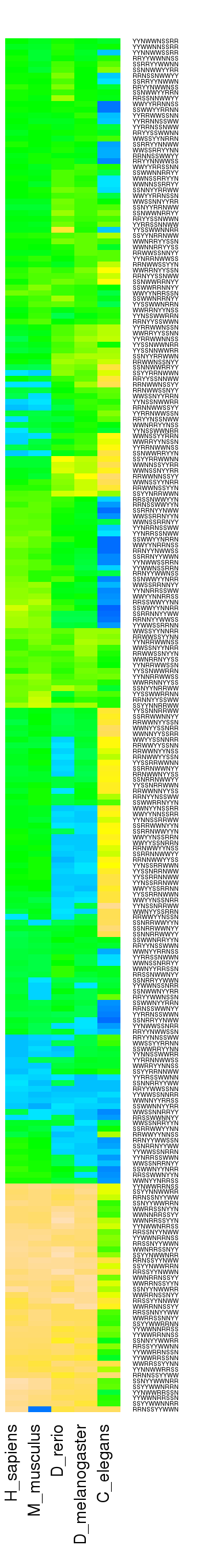

Supplement: S7 Fig — 10 bp frequency intensity for the 240 randomly generated sequences in the region +50 to +200 from the TSS of the organisms tested. (PNG) [file pcbi.1005144.s008.png]

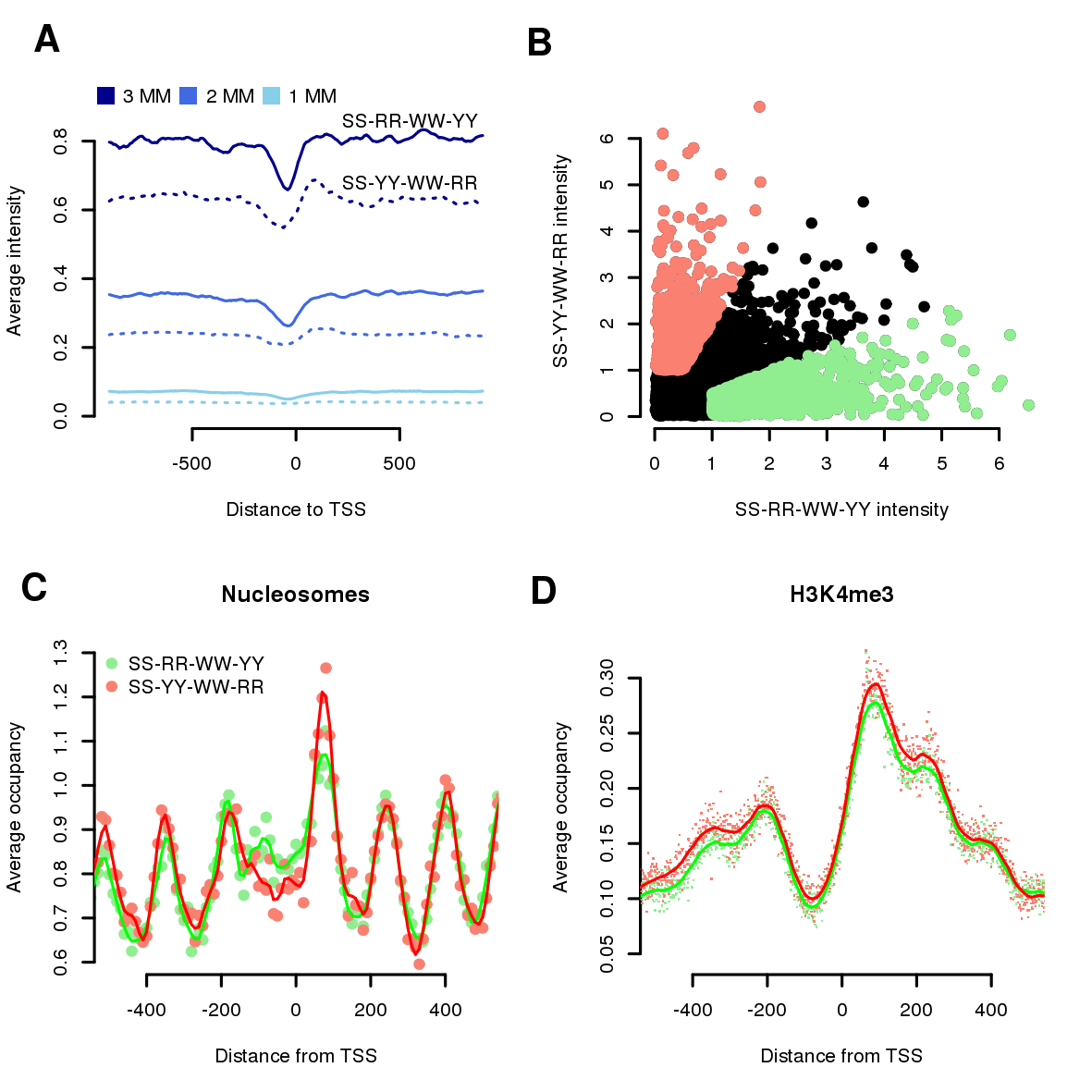

Supplement: S8 Fig — (A) Average 10 bp frequency intensities of two consensus classes (represented by the RRWWNNYYSS and YYWWNNRRSS consensuses for the SS-RR-WW-YY and SS-YY-WW-RR classes) around C. elegans promoters. Different shades of blue represent the total number of mismatches allowed in the mapping. (B) Intensity of the 10 bp frequency of the two consensuses on the N+1 region for each C. elegans promoters. Red and green dots highlight promoters characterized for a strong signal of the YYWWNNRRSS consensus (double the signal) compared to the RRWWNNYYSS consensus respectively. (C) Nucleosome distribution around promoters characterized for a strong signal in one consensus as defined by (B). Each dot represents the average tag count in a widow of 10 bp. Continuous lines are the local polynomial regression fit. (D) Same as (C) but with H3K4me3 and window of 1 bp. (PNG) [file pcbi.1005144.s009.png]

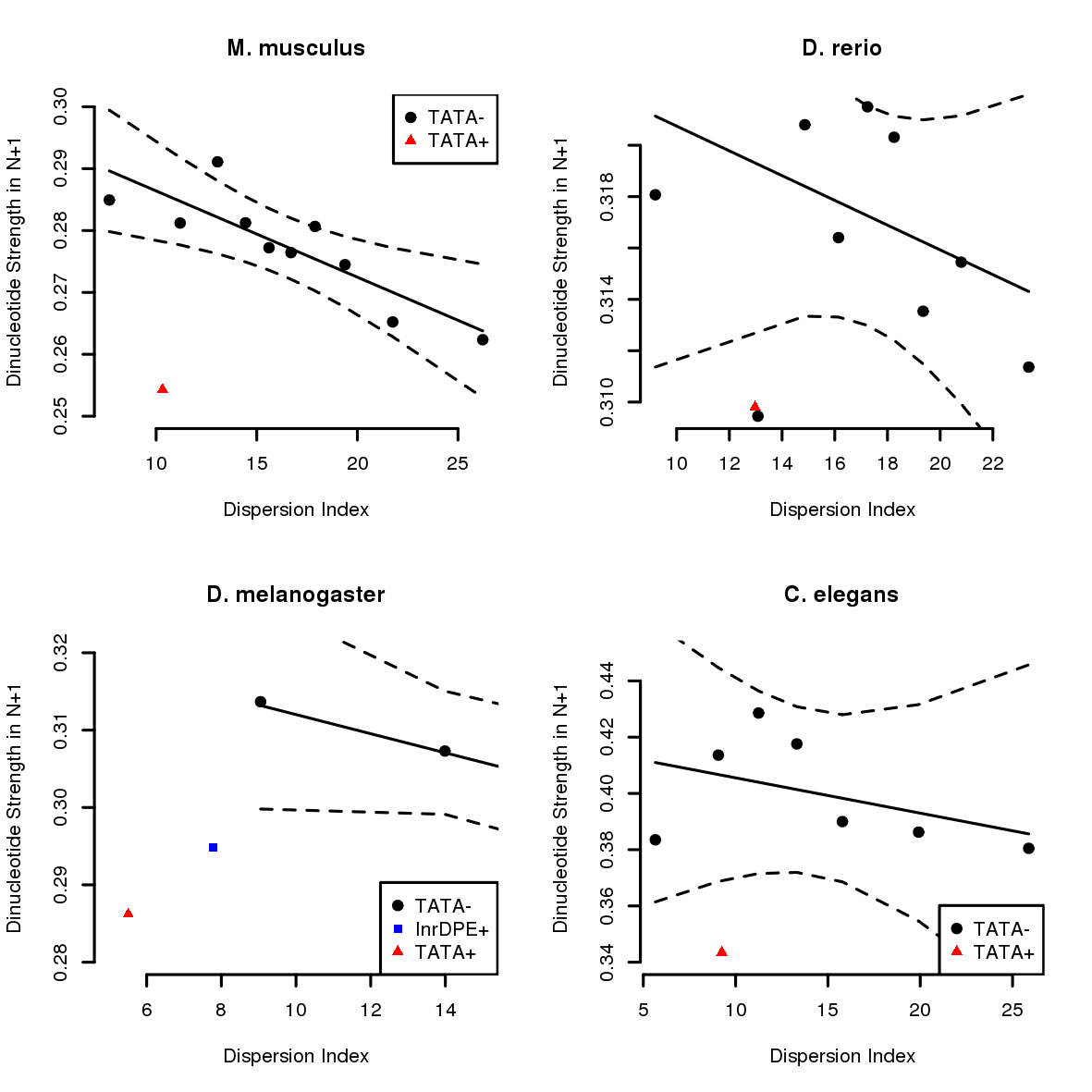

Supplement: S9 Fig — For each organism promoters were grouped according to their CPE status (TATA-box and Inr-DPE presence in the expected position). CPE-less promoters were also grouped according to their DI value in groups of 2000 promotes (a similar number as the promoters with the TATA-box). Each dot represents the average value of the intensity of the WW, SS, RR and YY dinucleotide for groups with similar DI and CPE status. Solid line represents the predicted values (evaluated by a linear model) whereas dotted line the 99% confidence intervals as evaluated by the linear model. (PNG) [file pcbi.1005144.s010.png]

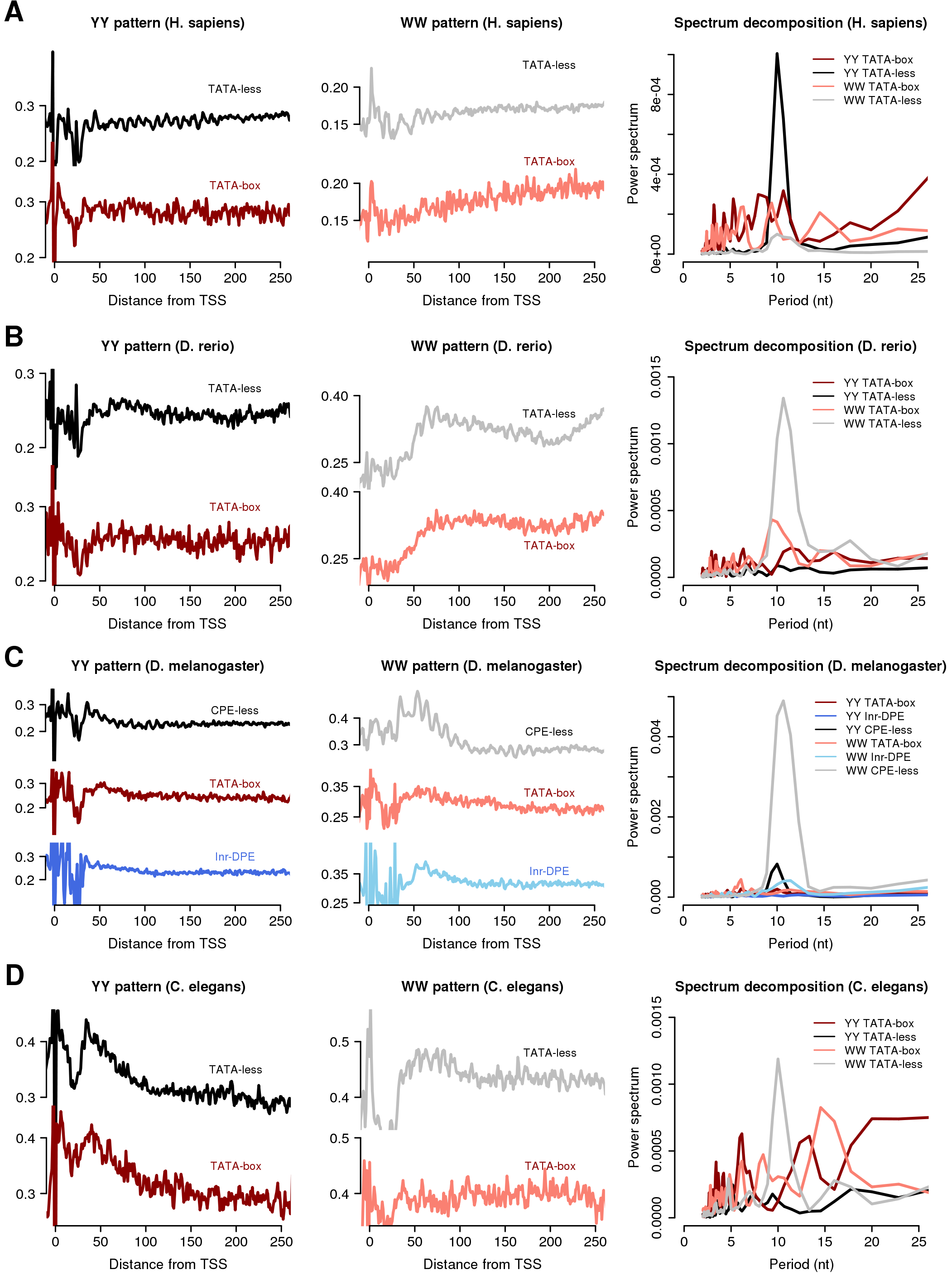

Supplement: S10 Fig — (A) YY dinucleotide frequencies for H. sapiens promoters with TATA-box (TATA-box) and without (TATA-less) (left panel); WW dinucleotide frequencies for the same promoters groups (central panel); and spectrum decomposition in the region +50 to +200 (right panel) for the 4 signals. (B) Same as A but for D. rerio promoters. (C) Similar to A but with D. melanogaster promoters stratified for the presence of the TATA-box (TATA-box), the absence of the TATA-box but the presence of Inr-DPE motif (Inr-DPE) and for the absence of both (CPE-less). (D) Same as (A) but with C. elegans promoters. (PNG) [file pcbi.1005144.s011.png]

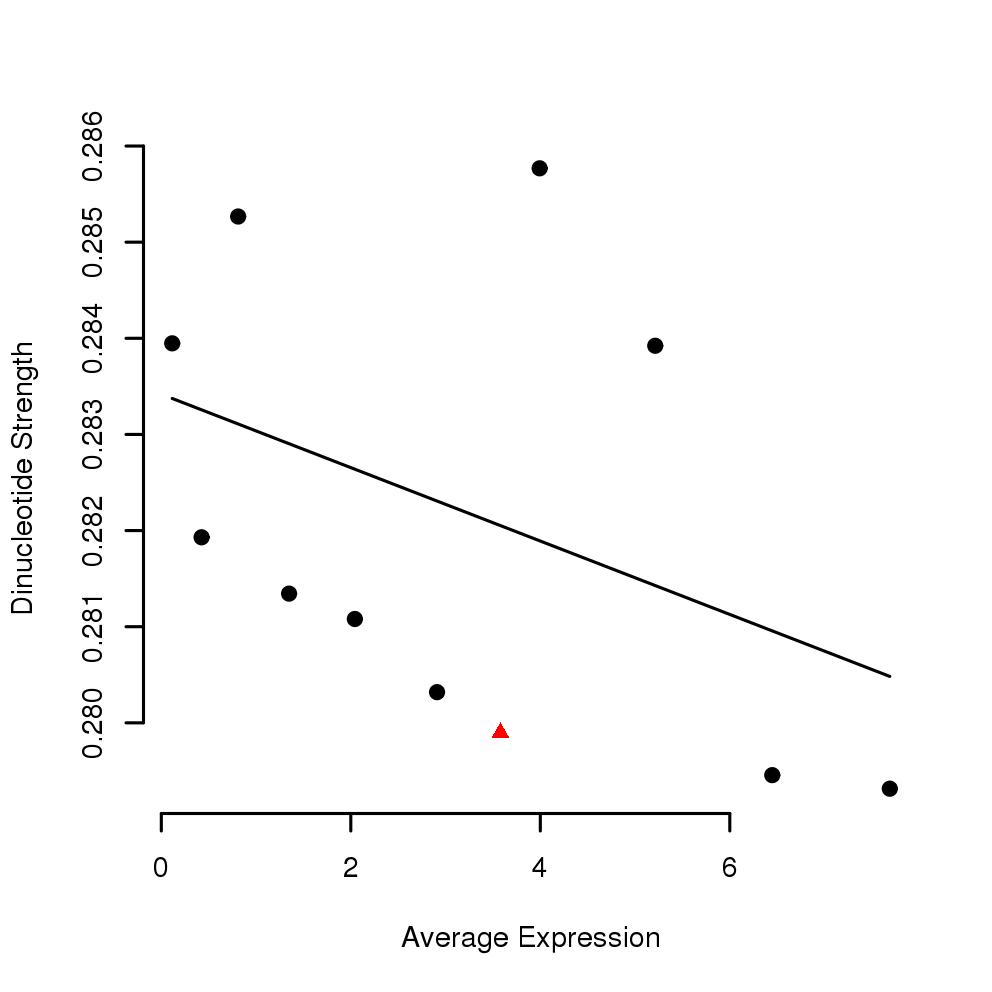

Supplement: S11 Fig — H. sapiens promoters were grouped following the same rules as in S9 Fig but using the average promoter expression instead of Dispersion Index. In this case, no correlation is seen between expression and average dinucleotide 10 bp frequency strength in the N+1 region. (JPG) [file pcbi.1005144.s012.jpg]

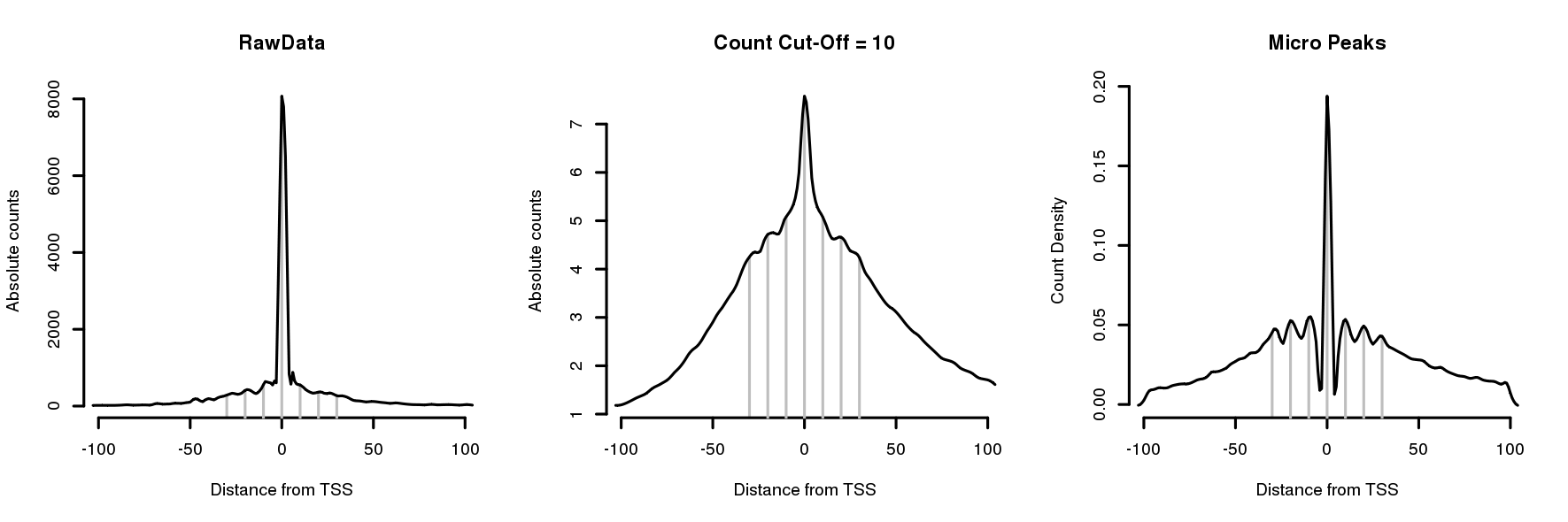

Supplement: S12 Fig — Vertical grey lines are separated by 10 bp from position -30 to 30 relative to the dominant TSS. Left panel: raw global CAGE distribution, periodic initiation is not clearly visible. Middle panel: CAGE distribution after a 10 tags count cut-off was applied to each position around each promoter, a 10 bp periodicity is starting to emerge from the data. Right panel: micro peak distribution, 10 bp periodic distribution is evident. (PNG) [file pcbi.1005144.s013.png]

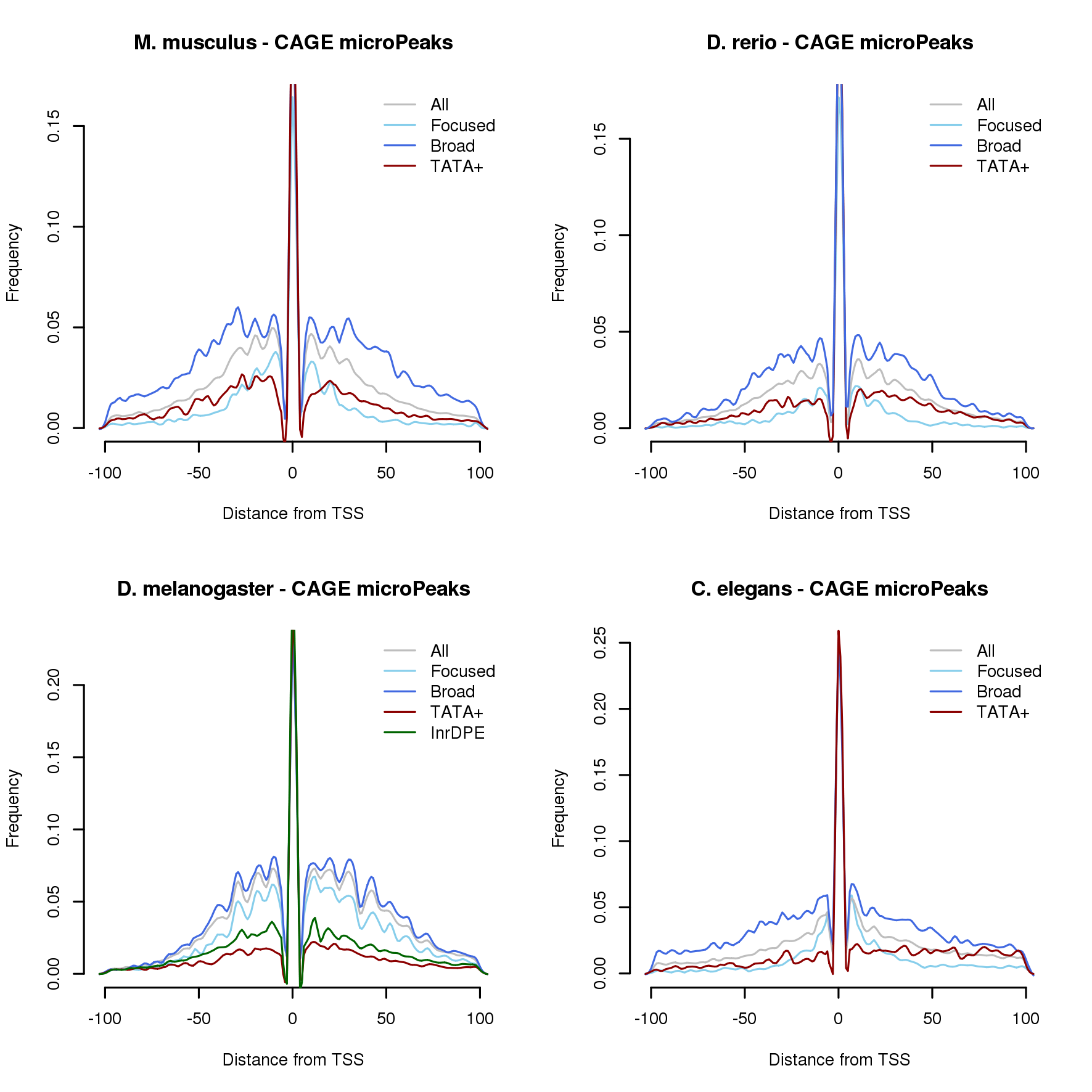

Supplement: S13 Fig — A strong 10 bp periodicity in Pol-II initiation is visible only in CPE-less promoters, reflecting the presence in the N+1 region of a DNA-encoded nucleosome signal. (PNG) [file pcbi.1005144.s014.png]

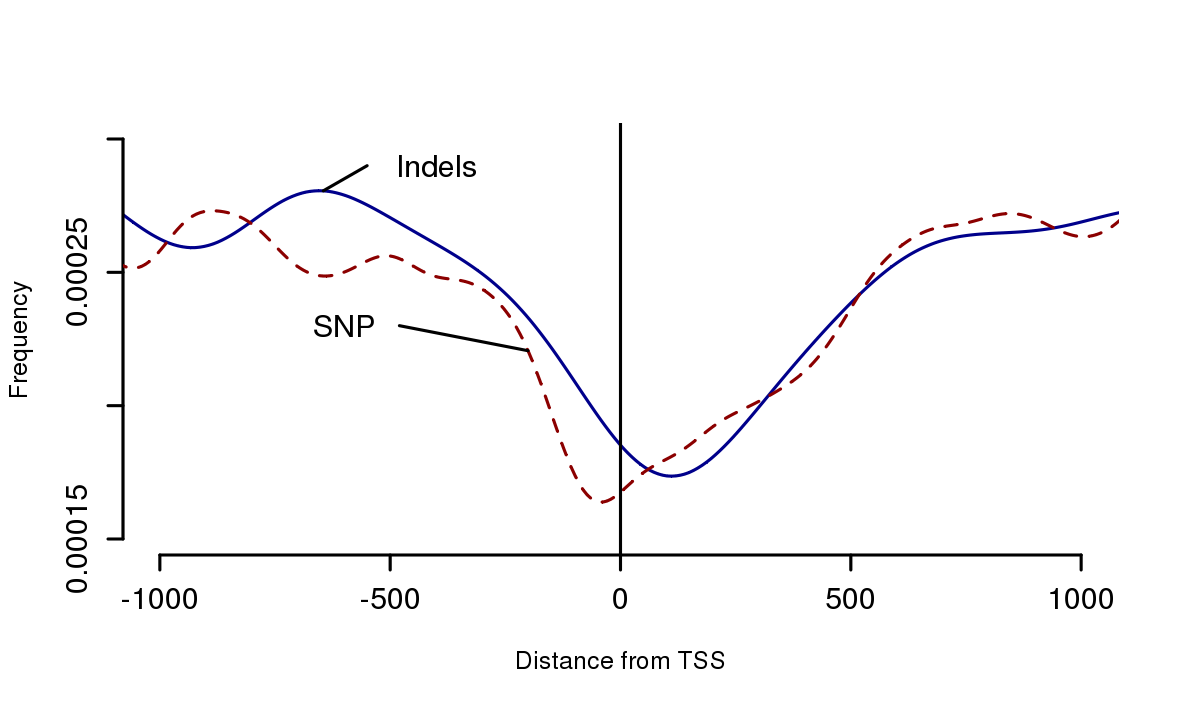

Supplement: S14 Fig — (PNG) [file pcbi.1005144.s015.png]
